# Supplementary material for: Unveiling the Taxonomic Diversity and Unprecedented Biosynthetic Treasure of the Phylum Myxococcota
Source: Adv Sci (Weinh). 2026 Aug 6;13(60):e76919. doi: 10.1002/advs.76919 (PMC13445942; doi:10.1002/advs.76919)
Supplement: Supplementary file 1 [file ADVS-13-e76919-s001.pdf]

Unveiling the taxonomic diversity and unprecedented biosynthetic treasure of the phylum *Myxococcota*

Supplementary Information

List of Supplementary Notes

Supplementary Note 1 Reclassification suggestions.………………………………………… 11-13
Supplementary Note 2 New *Myxococcota* Family Proposal ..…………………………… 26-27
Supplementary Note 3 Structure elucidation of myxolutamid A and identification of the putative *mxl* BGC ……………………………………………………. 29
Supplementary Note 4 Structure elucidation of sorangicin X and Y …………………………. 41
Supplementary Note 5 Analysis of the *srx* BGC and comparison to the *sor* BGC………. 59-60
Supplementary Note 6 Analysis of the expanded NCBI-derived *Myxococcota* dataset…. 66-67
Supplementary Note 7 Selecting the clustering thresholds for BiG-SCAPE And BiG-SLiCE ……………………………………………………… 71-72
Supplementary Note 8 Selection of *Actinomycetota* strains comparable to the in-house *Myxococcota* based on 16S DNA sequence………….……74
Supplementary Note 9 Structure elucidation of myxopentacin A.…………………………..….78
Supplementary Note 10 Biosynthesis of myxopentacin……………………………………..87

List of Supplementary Tables

Supplementary Table 1 Genome assembly statistics. (see separate file)
Supplementary Table 2 Type strain list (see separate file)
Supplementary Table 3 Classifications for the 154 new strains. (see separate file)
Supplementary Table 4 Reclassified *Mycoccocota* Taxonomy (see separate file)
Supplementary Table 5 NMR spectroscopic data of myxolutamid A 31
Supplementary Table 6 Open reading frames in the putative *mxl* BGC from *Pendulispora* MSr11367 with proposed function and closest homologues.. 40
Supplementary Table 7 NMR spectroscopic data of sorangicin X... 42
Supplementary Table 8 ^{1}H NMR comparison of sorangicin X and sorangicin A...................... 44
Supplementary Table 9 ^{13}C NMR comparison of sorangicin X and sorangicin A. 46
Supplementary Table 10 NMR spectroscopic data of sorangicin Y in methanol-d_4.................. 48
Supplementary Table 11 Comparison of the cluster architecture of the *srx* BGC. 61
Supplementary Table 12 Biological activities of sorangicin X and Y. 64
Supplementary Table 13 Summary of 16S and ANI-based matches for NCBI sequence entries with newly sequenced *Myxococcota* strains and type strains (see separate file)
Supplementary Table 14 Comparison of clusterings produced at different threshold values for BiG-SCAPE with the ground truth based on compound similarity for data from MIBiG. 71
Supplementary Table 15 Comparison of clusterings produced at different threshold values for BiG-SLICE with the ground truth based on BiG-SCAPE clustering at threshold 0.5 for the same dataset. 72
Supplementary Table 16 NMR spectroscopic data of myxopentacin A. 79
Supplementary Table 17 Genes detected in the *mxp*BGC from *Myxococcus* MCy9003 with their closest protein homologues. 89
Supplementary Table 18 Plasmids and oligonucleotides used in this study............................ 91
Supplementary Table 19 BioProject, BioSample and Assembly accessions of strains submitted at NCBI.. 93

List of Supplementary Figures

Supplementary Figure 1 The averaged pairwise POCP values of families and genera. 5
Supplementary Figure 2 Distribution of average inter- and intra-species POCP values on genus and family levels. 6
Supplementary Figure 3 The pairwise POCP values of 109 Myxococcota type strains at a genus cut-off of 76%.. 7
Supplementary Figure 4 The pairwise POCP values of 109 *Myxococcota* type strains at a genus cut-off from 71 to 79%. 8
Supplementary Figure 5 Phylogenomic reconstruction by the 109 *Myxococcota* type strains ... 9
Supplementary Figure 6 Phylogenomic reconstruction of 154 *Myxococcota* strains and type strains. 10
Supplementary Figure 7 The reclassified family *Myxococcaceae*. 14
Supplementary Figure 8 The reclassified family *Plesiocystaceae*. 15
Supplementary Figure 9 The reclassified family *Labilitrichaceae*, *Phaselicystaceae* and *Polyangiaceae*. 16
Supplementary Figure 10 The reclassified genus *Anaeromyxobacter.* 17
Supplementary Figure 11 The reclassified genus *Haliangium.* 18
Supplementary Figure 12 The reclassified genus *Myxococcus.* 19
Supplementary Figure 13 The reclassified genus *Nannocystis.* 20
Supplementary Figure 14 The reclassified genus *Pendulispora.* 21
Supplementary Figure 15 The reclassified genus *Polyangium.* 22
Supplementary Figure 16 The reclassified genus *Sorangium.* 23
Supplementary Figure 17 The reclassified genera *Archangium* and *Vitiosangium.* 24
Supplementary Figure 18 The reclassified genera *Cystobacter* and *Melittangium*. 25
Supplementary Figure 19 Distribution of COG categories among core genes and family-specific genes. 28
Supplementary Figure 20 Chemical structure of myxolutamid A. 30
Supplementary Figure 21 ^{1}H-spectrum of myxolutamid. 32
Supplementary Figure 22 COSY-spectrum of myxolutamid A. 33
Supplementary Figure 23 HSQC-spectrum of myxolutamid A. 34
Supplementary Figure 24 HMBC-spectrum of myxolutamid A. 35
Supplementary Figure 25 MS spectrum for myxolutamid A. 36
Supplementary Figure 26 MS^2 fragment spectrum for myxolutamid A. 36
Supplementary Figure 27 Myxolutamid molecular network generated in GNPS. 37
Supplementary Figure 28 Marfey's derivatization of reference L-alanine and myxolutamid A. 38
Supplementary Figure 29 Marfey's derivatization of reference L-leucine and myxolutamid A. 38
Supplementary Figure 30 Putative *mxl*BGC from *Pendulispora* MSr11367. 39
Supplementary Figure 31 Chemical structure of sorangicin X 42
Supplementary Figure 32 Structural comparison of sorangicins X and Y with sorangicin A. .. 44
Supplementary Figure 33 Chemical structure of sorangicin Y. 48
Supplementary Figure 34 ^{1}H-spectrum of sorangicin X. 50
Supplementary Figure 35 ^{13}C-spectrum of sorangicin X. 50
Supplementary Figure 36 COSY spectrum of sorangicin X. 51
Supplementary Figure 37 HSQC spectrum of sorangicin X. 52

Supplementary Figure 38 HMBC spectrum of sorangicin X. 53
Supplementary Figure 39 ^{1}H-spectrum of sorangicin Y. 54
Supplementary Figure 40 ^{13}C-spectrum of sorangicin Y. 54
Supplementary Figure 41 COSY spectrum of sorangicin Y. 55
Supplementary Figure 42 HSQC spectrum of sorangicin Y. 56
Supplementary Figure 43 HMBC spectrum of sorangicin Y. 57
Supplementary Figure 44 MS spectrum of sorangicin X. 58
Supplementary Figure 45 MS spectrum of sorangicin Y. 58
Supplementary Figure 46 The *srx* BGC and the biosynthesis of sorangicins X and Y compared to the sor BGC and the biosynthesis of sorangicin A. 60
Supplementary Figure 47 Distribution of characterized *Myxococcota* biosynthetic gene clusters across taxonomic groups. 65
Supplementary Figure 48 Histogram showing the size distribution of genomic fragments in NCBI sequencing entries. 67
Supplementary Figure 49 Composition of MAGs and isolates among NCBI sequencing entries from *Myxococcota* phylum 68
Supplementary Figure 50 Distribution of BGCs into major biosynthetic classes across four *Myxococcota* datasets. 69
Supplementary Figure 51 Upset plot showing the number of unique and shared GCFs between different *Myxococcota* datasets. 70
Supplementary Figure 52 Upset plot showing the number of unique and shared GCFs between different taxa and databases considered in this study. 73
Supplementary Figure 53 Ribbon plot showing the overlap of GCFs between different genera in *Myxococcota* and public *Myxococcota* genomes. 75
Supplementary Figure 54 Swarm plot showing the number of new GCFs added by individual strains in each genus. 76
Supplementary Figure 55 Inactivation mutant of *mxpA*. 77
Supplementary Figure 56 Chemical structure of myxopentacin A. 79
Supplementary Figure 57 ^{1}H-spectrum of myxopentacin A. 81
Supplementary Figure 58 COSY-spectrum of myxopentacin A. 82
Supplementary Figure 59 HSQC-spectrum of myxopentacin A. 83
Supplementary Figure 60 HMBC-spectrum of myxopentacin A. 84
Supplementary Figure 61 MS spectrum for myxopentacin A. 85
Supplementary Figure 62 MS^2 fragment spectrum for myxopentacin A. 85
Supplementary Figure 63 Marfey's derivatization of reference ACPC (cispentacin) and myxopentacin A. 86
Supplementary Figure 64 Marfey's derivatization of reference L-valine and myxopentacin A. 86
Supplementary Figure 65 *mxp* BGC from *Myxococcus* MCy9003. 88
Supplementary Figure 66 Inactivation of mxpA. 90
Supplementary Figure 67 Upset plot showing the number of unique and shared GCFs between different sub-orders belonging to *Myxococcota* phylum. 92

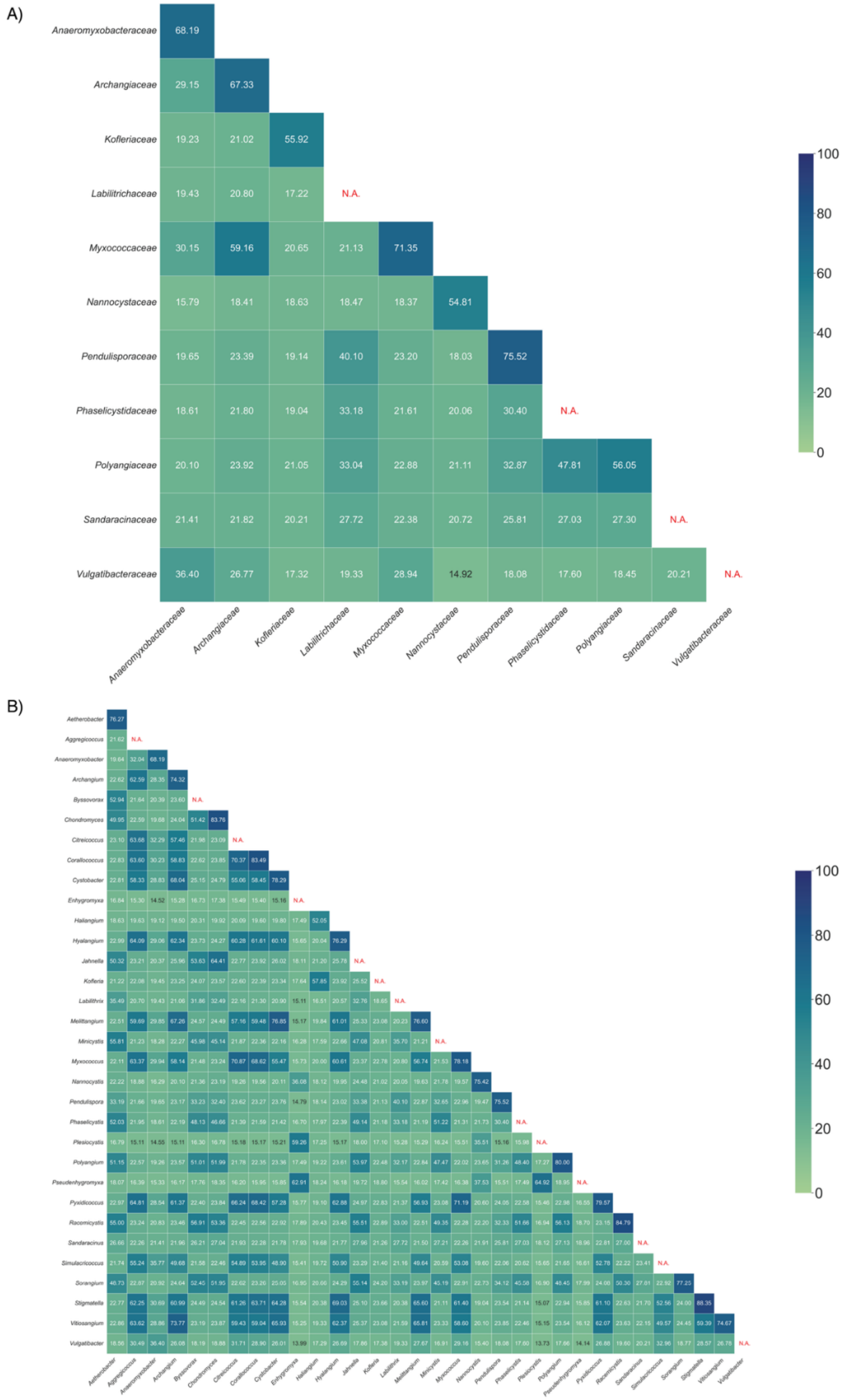

Supplementary Figure 1 The averaged pairwise POCP values of (A) families and (B) genera calculated by *Myxococcota* type strains. Single-member taxa, for which intra-taxon POCP cannot be calculated, are indicated as 'N.A.'.

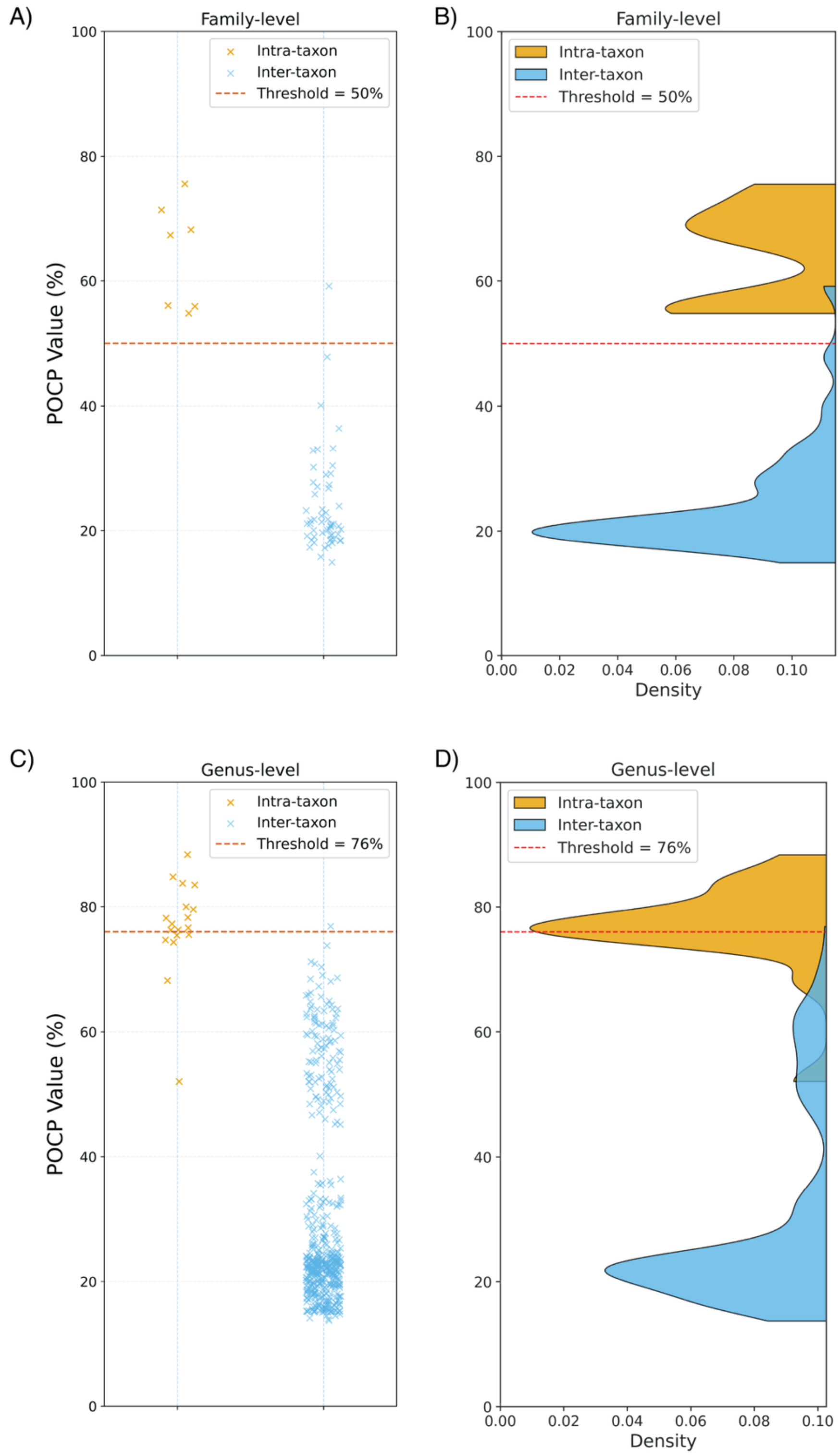

Supplementary Figure 2 Distribution of average inter- and intra-species POCP values on genus and family levels of *Myxococcota* type-strains. (A) Scatter plot of average inter- and intra-species POCP values on family level; (B) Kernal density plot of average inter- and intra-species POCP values on family level; (C) Scatter plot of average inter- and intra-species POCP values on genus level; (D) Kernal density plot of average inter- and intra-species POCP values on genus level.

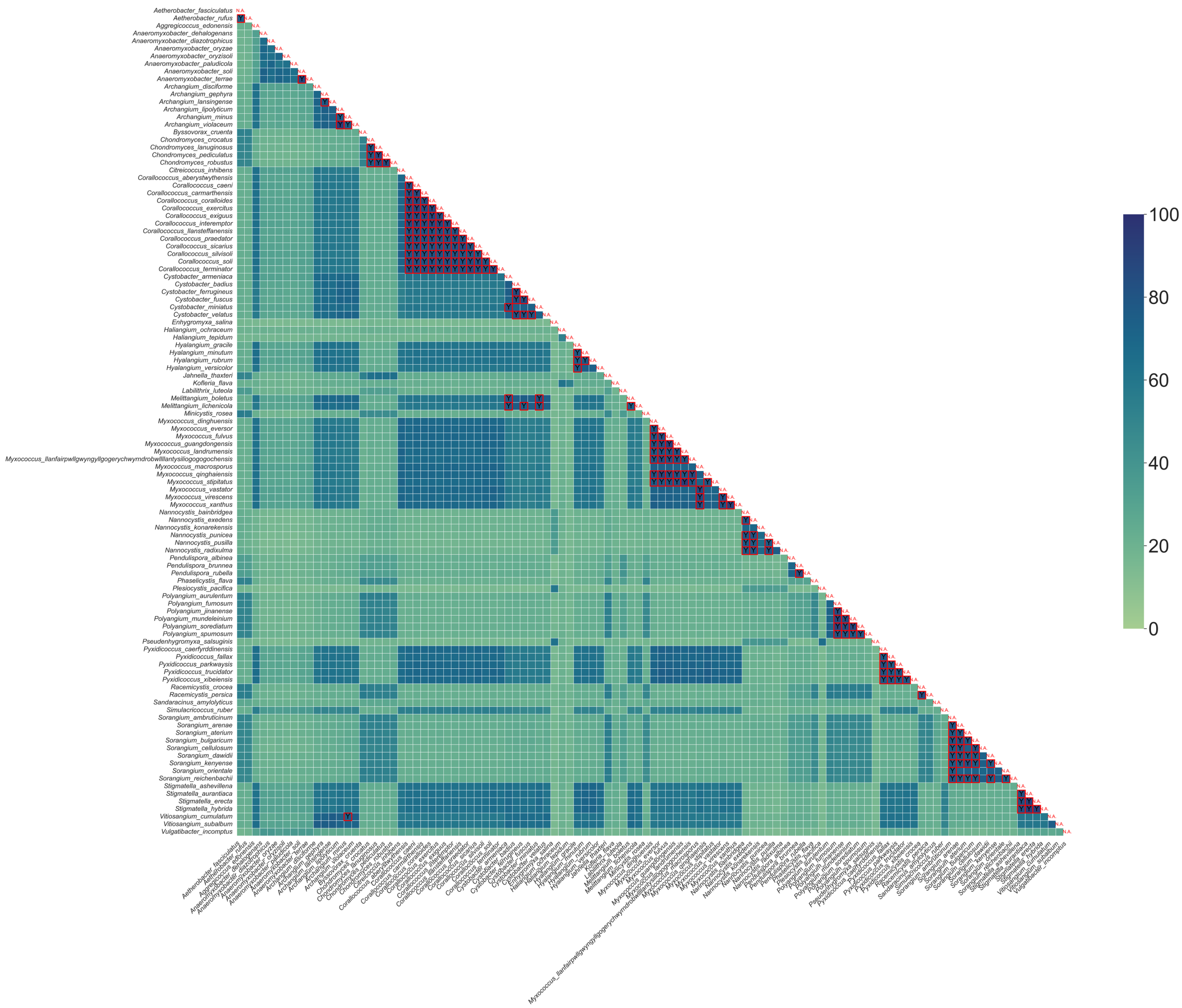

Supplementary Figure 3 The pairwise POCP values of 109 representative *Myxococcota* type strains. Cells marked with red boxes and 'Y' represent the POCP value equal or over the threshold of 76%. Self comparisons, for which intra-taxon POCP cannot be calculated, are indicated as 'N.A.'.

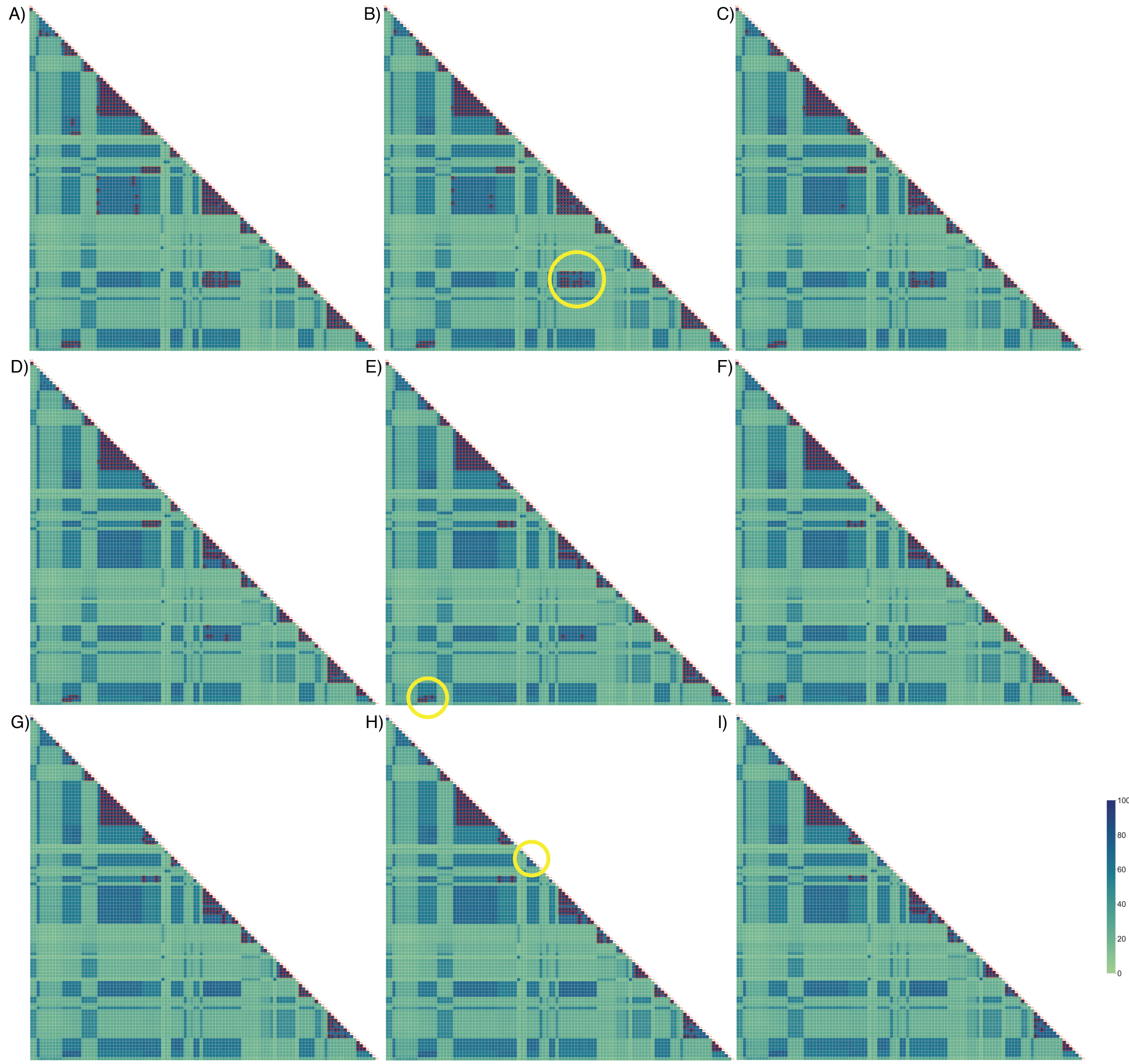

Supplementary Figure 4 The pairwise POCP values of 109 representative *Myxococcota* type strains. **(A-I)** represents pairwise POCP matrix for different genus level POCP thresholds (71% to 79%, with 1% increment). Cells marked with red boxes and 'Y' represent the POCP value equal or over the threshold. Self comparisons, for which intra-taxon POCP cannot be calculated, are indicated as 'N.A.'. Yellow circles represent the overrepresentation (B: 72% and E: 75%) and underrepresentation (H: 78%) of the corresponding POCP thresholds. In general, threshold 76% (F) shows the best intra-genus and inter-genus separation.

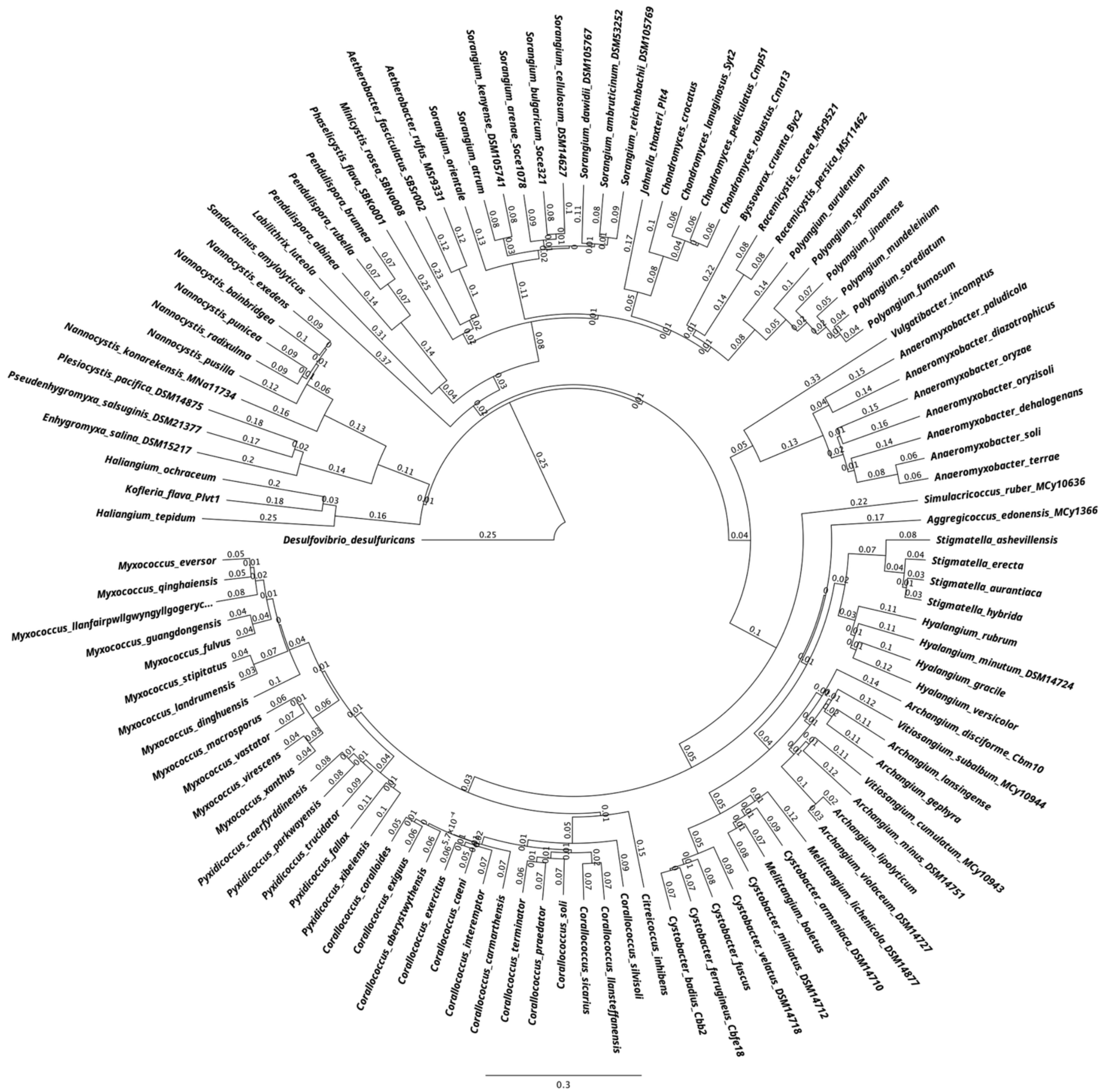

Supplementary Figure 5 Phylogenomic reconstruction by the 109 *Myxococcota* type strains using BIONJ from the pairwise POCP distances. *Desulfovibrio desulfuricans* serves as the outgroup. Leaves are annotated with the established taxonomy at species level.

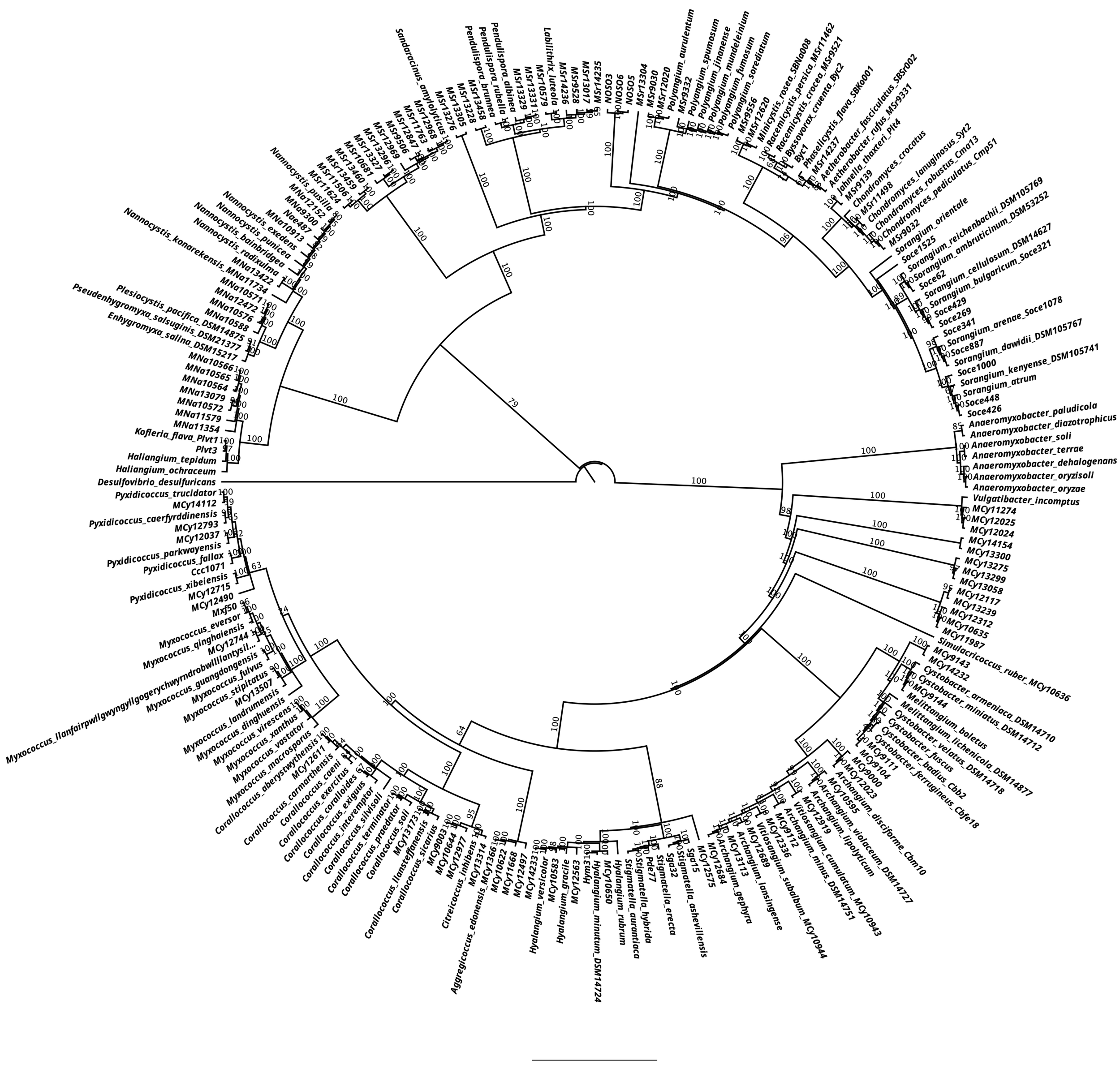

Supplementary Figure 6 Phylogenomic reconstruction of 154 *Myxococcota* strains and type strains based on single-copy orthologs using a maximum-likelihood approach. *Desulfovibrio desulfuricans* serves as the outgroup. Leaves annotated with both species names and strain identifiers denote novel genomes proposed as type strain representatives.

Supplementary Note 1 Reclassification suggestions.

Reclassification suggestions on the Family level

- *Myxococcaceae* and *Archangiaceae* united as one family *Myxococcaceae* (Supplementary Figure 7)
- *Enhygromyxa*, *Plesiocystis*, and *Pseudenhygromyxa* belong a new family *Plesiocystaceae*, separated from *Nannocystaceae* (Supplementary Figure 8)
- *Labilitrichaceae*, *Phaselicystaceae,* and *Polyangiaceae* are three separate families. *Aetherobacter* and *Minicystis* belong to *Phaselicystaceae* (Supplementary Figure 9)

Reclassification suggestions on the Genus level

- ***Anaeromyxobacter* split into 6 genera (Supplementary Figure 10):**

  (Original) *Anaeromyxobacter*:
    Species: *Anaeromyxobacter dehalogenans* (Type Species)
  Anaeromyxobacter-derived New Genus 1:
    Species: *Anaeromyxobacter diazotrophicus*
  Anaeromyxobacter-derived New Genus 2:
    Species: *Anaeromyxobacter oryzae*
  Anaeromyxobacter-derived New Genus 3:
    Species: *Anaeromyxobacter oryzisoli*
  Anaeromyxobacter-derived New Genus 4:
    Species: *Anaeromyxobacter paludicola*
  Anaeromyxobacter-derived New Genus 5:
    Species: *Anaeromyxobacter soli, Anaeromyxobacter terrae*

- ***Haliangium* split into 2 genera (Supplementary Figure 11):**
  (Original) *Haliangium*:
    Species: *Haliangium ochraceum*
  Haliangium-derived New Genus 1:
    Species: *Haliangium tepidum*

- ***Myxococcus* split into 2 genera (Supplementary Figure 12):**
  (Original) *Myxococcus*:
    Species: *Myxococcus stipitatus*, *Myxococcus landrumensis*, *Myxococcus guangdongensis*, *Myxococcus fulvus* (Type Species), *Myxococcus llanfairpwllgwyngyllgogerychwyrndrobwllllantysiliogogogochensis, Myxococcus qinghaiensis, Myxococcus eversor, Myxococcus dinghuensis*
  Myxococcus-derived New Genus 1: Proposed *Pseudomyxococcus*[1]
    Species: *Myxococcus xanthus*, *Myxococcus virescens*, *Myxococcus vastator*, *Myxococcus macrosporus*

- ***Nannocystis* split into 2 genera (Supplementary Figure 13):**
  (Original) *Nannocystis*:

Species: *Nannocystis bainbridgea*, *Nannocystis exedens* (Type Species), *Nannocystis punicea*, *Nannocystis pusilla*, *Nannocystis radixulma*

Nannocystis-derived New Genus 1:

Species: *Nannocystis konarekensis*

- ***Pendulispora* split into 2 genera (Supplementary Figure 14):**

(Original) *Pendulispora*:

Species: *Pendulispora albinea*

Pendulispora-derived New Genus 1:

Species: *Pendulispora brunnea*, *Pendulispora rubella*

- ***Polyangium* split into 2 genera (Supplementary Figure 15):**

(Original) *Polyangium*:

Species: *Polyangium fumosum*, *Polyangium jinanense*, *Polyangium mundeleinium*, *Polyangium sorediatum*, *Polyangium spumosum* (Type Species), *Polyangium vitellinum*'s genome is not available. We refer it as *Polyangium* based on previous publication[2].

Polyangium-derived New Genus 1:

Species: *Polyangium aurulentum*

- ***Sorangium* split into 4 genera (Supplementary Figure 16):**

(Original) *Sorangium:*

Species: *Sorangium arenae, Sorangium bulgaricum, Sorangium cellulosum* (Type Species)

Sorangium-derived New Genus 1:

Species: *Sorangium aterium*, *Sorangium kenyense*

Sorangium-derived New Genus 2:

Species: *Sorangium dawidii*, *Sorangium ambruticinum*, *Sorangium reichenbachii*

Sorangium-derived New Genus 3:

Species: *Sorangium orientale*

- ***Archangium-Vitiosangium* complex (Supplementary Figure 17):**

Reclassification back of *Archangium disciforme* to the original genus *Angiococcus*

Genus *Archangium* includes the following species:

Archangium gephyra (Type species)
Archangium lansingense
Vitiosangium subalbum, reclassify to *Archangium subalbum*

Genus *Vitiosangium* includes the following species:

Vitiosangium cumulatum (Type species)
Archangium minus, reclassify to *Vitiosangium minus*
Archangium violaceum, reclassify to *Vitiosangium violaceum*
Archangium. lipolyticum, reclassify to *Vitiosangium lipolyticum*

- ***Cystobacter-Melittangium* complex (Supplementary Figure 18):**

  Genus *Cystobacter* includes the following species:
    - *Cystobacter fuscus* (Type species)
    - *Cystobacter ferrugineus*
    - *Cystobacter badius*
    - *Cystobacter velatus*

  Genus *Melittangium* includes the following species:
    - *Melittangium lichenicola*
    - *Melittangium boletus* (Type species)
    - *Cystobacter miniatus*, reclassify to *Melittangium miniatus*
    - *Cystobacter armeniaca*, reclassify to *Melittangium armeniaca*
    - *Melittangium alboraceum* (Type strain genome not available) We refer it as *Melittangium* based on previous publication[3]

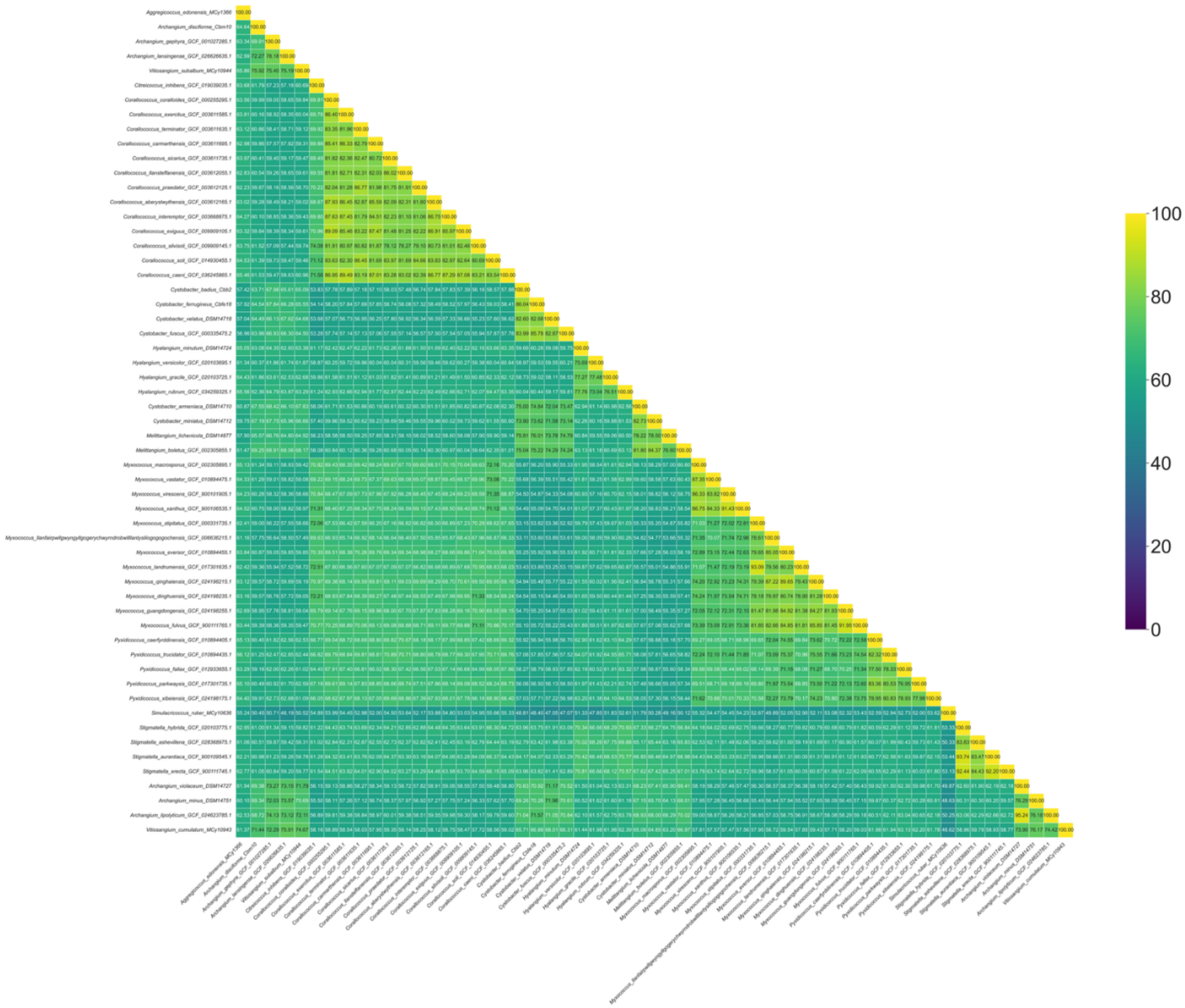

Supplementary Figure 7 The reclassified family *Myxococcaceae*. The pairwise POCP values of species within the family *Myxococcaceae* and *Archangiaceae* leading to a suggested merge to one family *Myxococcaceae.* All species reclassified as *Myxococcaceae* shown here.

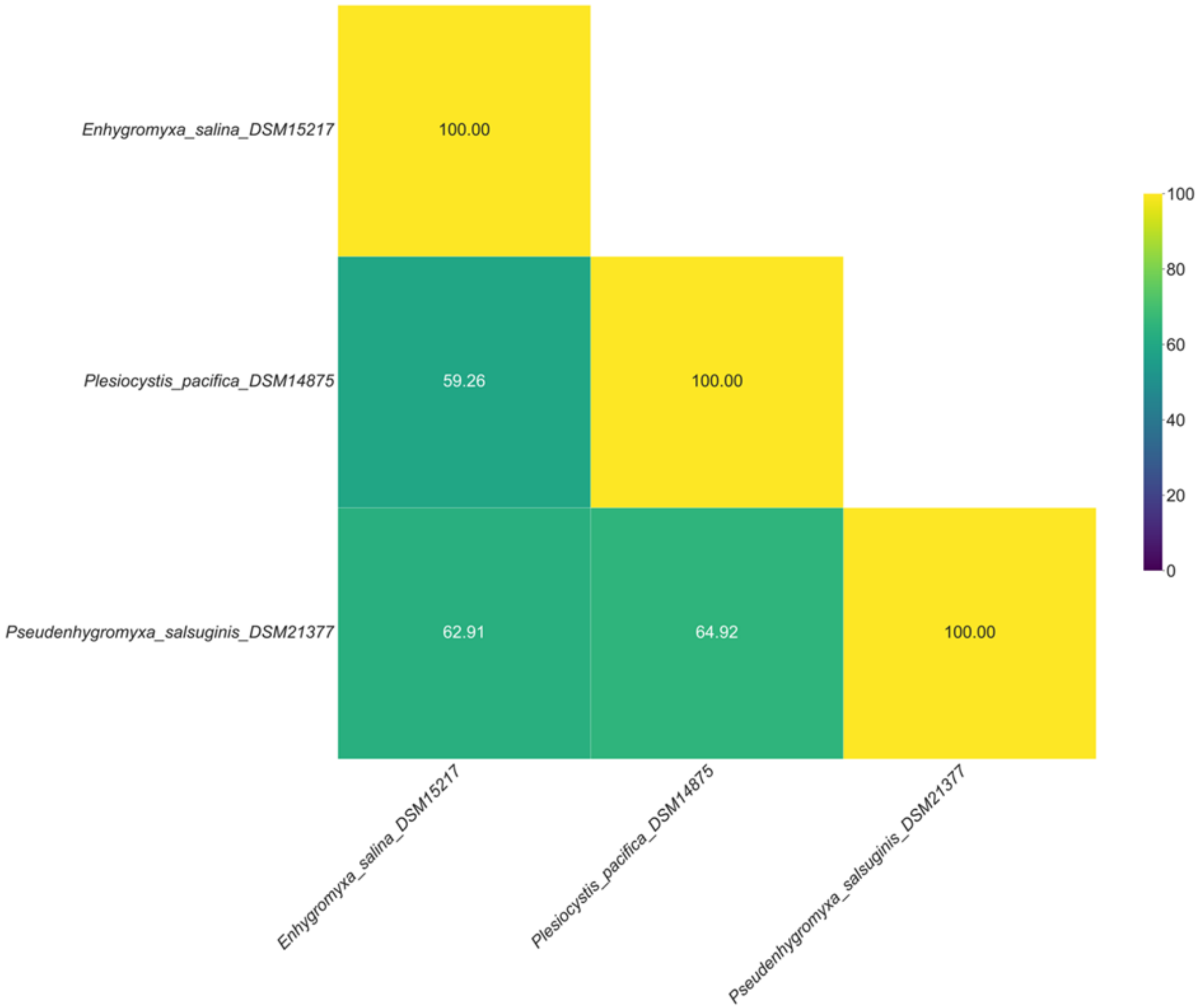

Supplementary Figure 8 The *Plesiocystaceae*. The pairwise POCP values of species within *Enhygromyxa, Plesiocystis, and Pseudenhygromyxa* suggest they form a new family (*Plesiocystaceae*) separated from *Nannocystaceae.* All species reclassified as *Plesiocystaceae* are shown here.

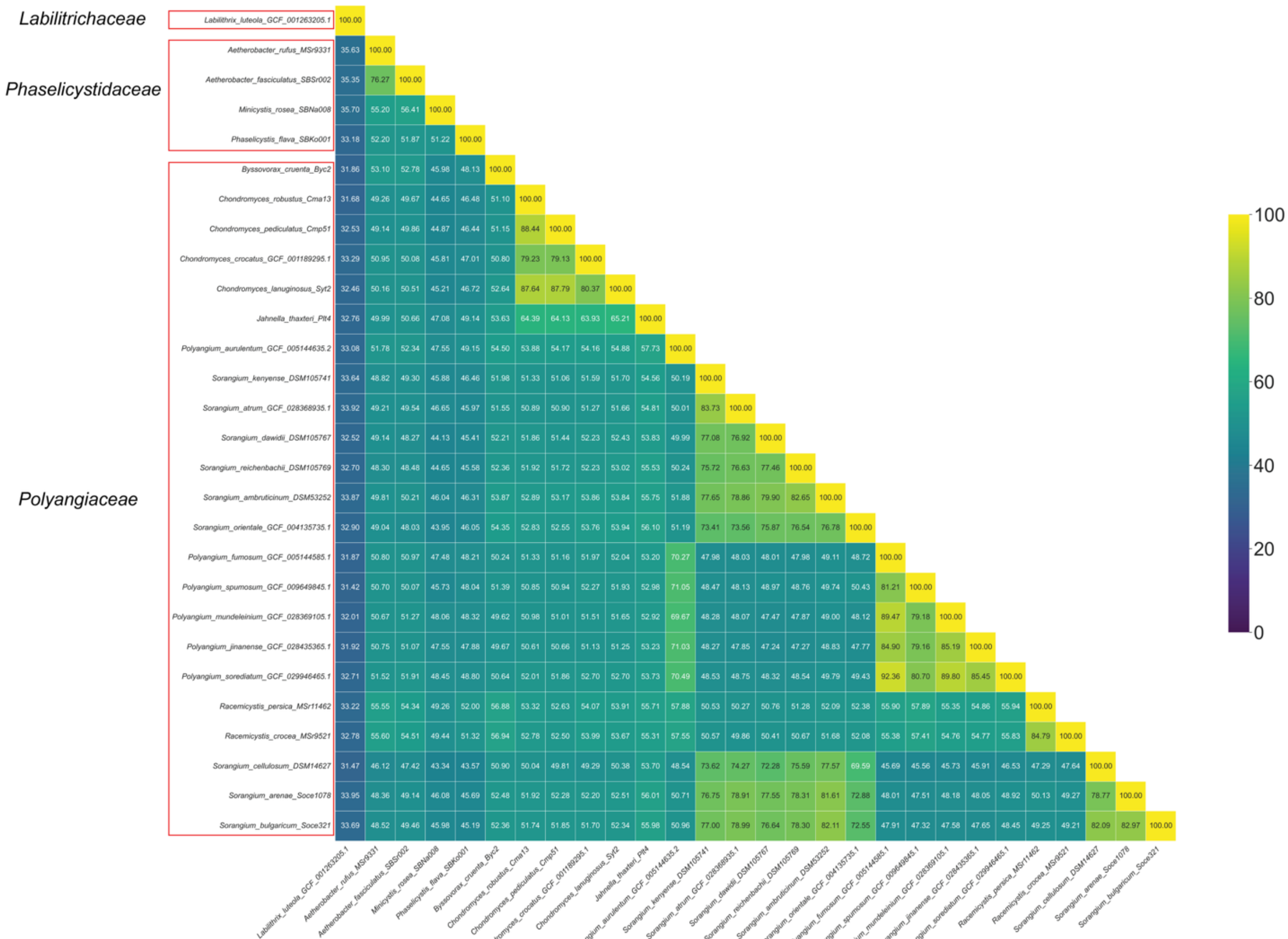

Supplementary Figure 9 The reclassified family *Labilitrichaceae*, *Phaselicystaceae* and *Polyangiaceae.* The pairwise POCP values of species within *Labilitrichaceae, Phaselicystaceae, and Polyangiaceae* suggest they are three separate families. *Aetherobacter* and *Minicystis* belong to *Phaselicystaceae.* All species reclassified as *Labilitrichaceae*, *Phaselicystaceae* and *Polyangiaceae* shown here.

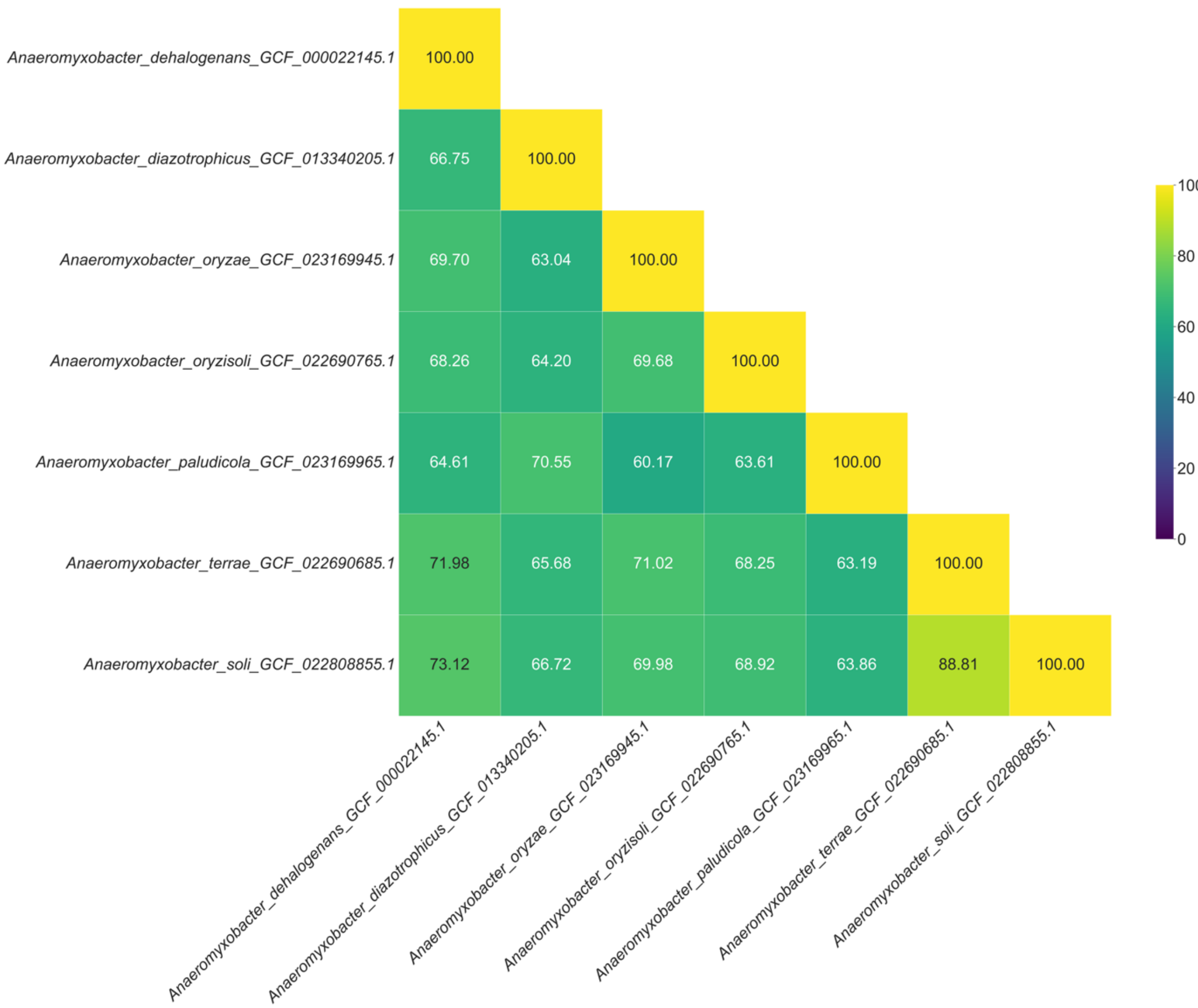

Supplementary Figure 10 The reclassified genus *Anaeromyxobacter.* The pairwise POCP values of species within *Anaeromyxobacter* suggest they are six separate genera: *Anaeromyxobacter* (represented by *Anaeromyxobacter dehalogenans* (Type species)), *New Genus 1* (represented by *Anaeromyxobacter diazotrophicus*), *New Gebnus 2* (represented by *Anaeromyxobacter oryzae*), *New Genus 3* (represented by *Anaeromyxobacter oryzisoli*), *New Genus 4* (represented by *Anaeromyxobacter paludicola*), *New Genus 5* (represented by *Anaeromyxobacter soli and Anaeromyxobacter terrae*).

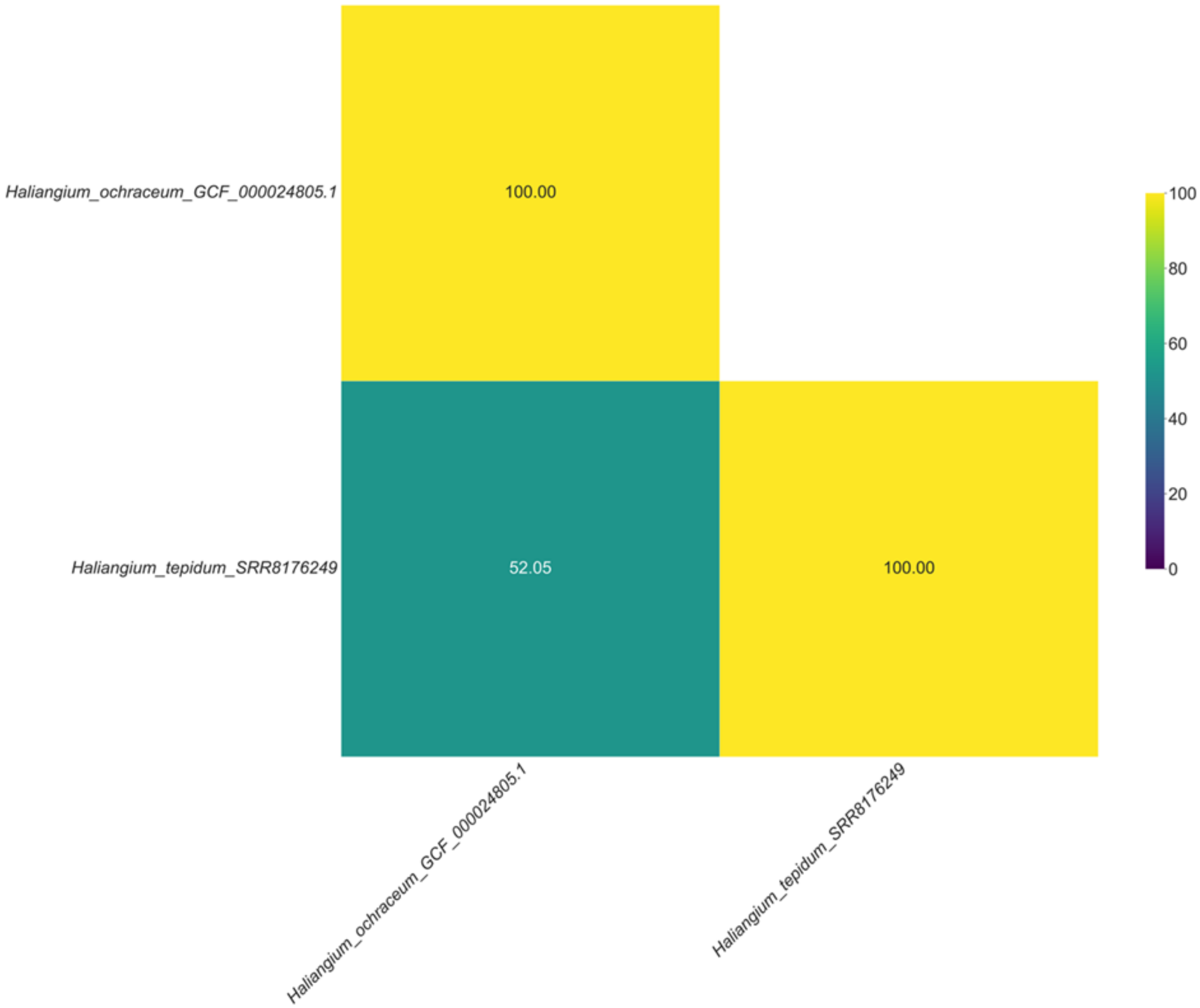

Supplementary Figure 11 The reclassified genus *Haliangium.* The pairwise POCP values of species within *Haliangium* suggest splitting the genus in two genera: *Haliangium* (represented by *Haliangium ochraceum)* and New Genus 1 (represented by *Haliangium tepidum)*.

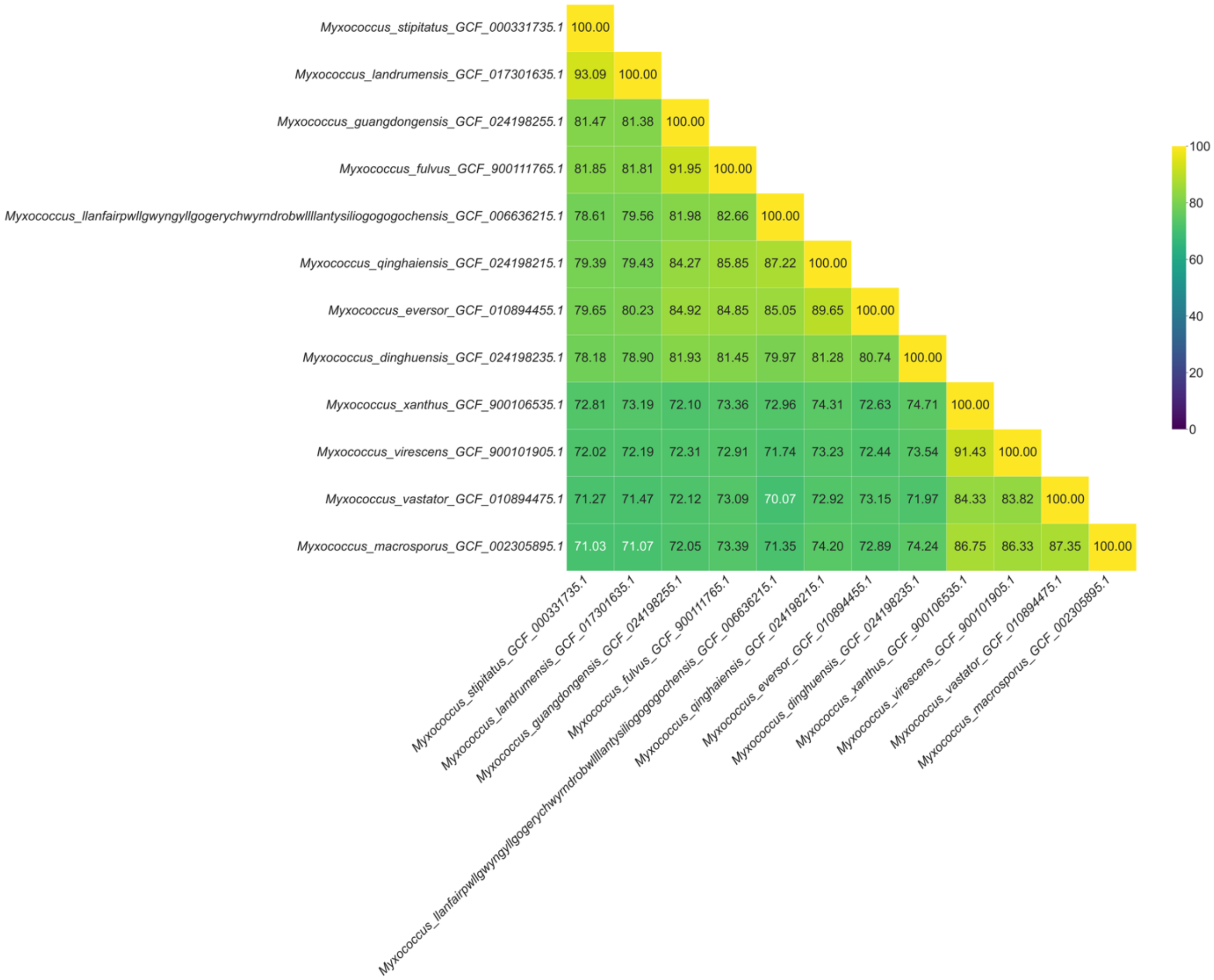

Supplementary Figure 12 The reclassified genus *Myxococcus.* The pairwise POCP values of species within *Myxococcus* suggest splitting the genus in two genera: *Myxococcus* (represented by *Myxococcus stipitatus*, *Myxococcus landrumensis*, *Myxococcus guangdongensis*, *Myxococcus fulvus* (Type species)*, Myxococcus llanfairpwllgwyngyllgogerychwyrndrobwllllantysiliogogogochensis, Myxococcus qinghaiensis, Myxococcus eversor,* and *Myxococcus dinghuensis)* and New Genus 1 (*Pseudomyxococcus;* represented by *Myxococcus xanthus*, *Myxococcus virescens*, *Myxococcus vastator*, *Myxococcus macrosporus)*

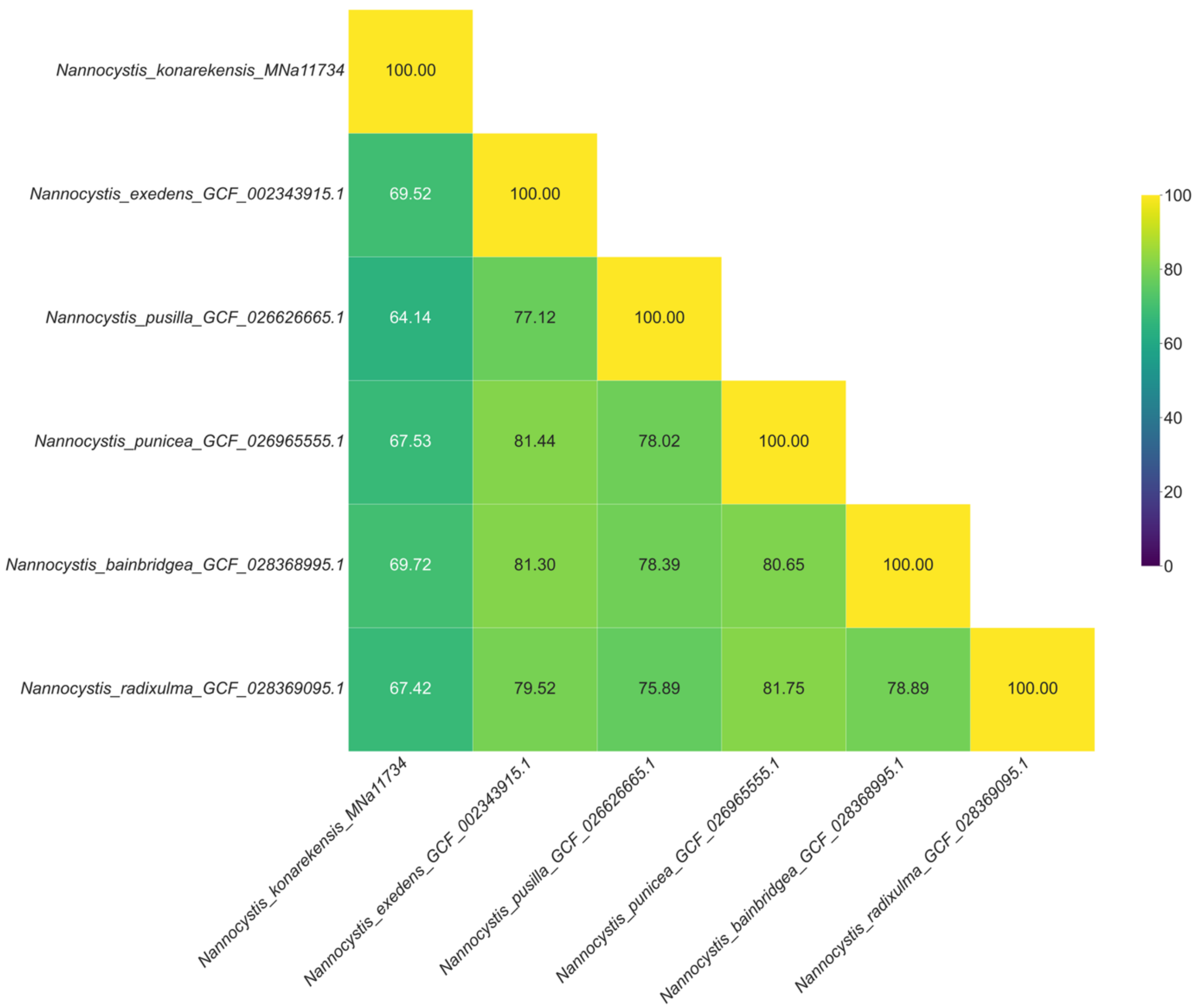

Supplementary Figure 13 The reclassified genus *Nannocystis.* The pairwise POCP values of species within *Nannocystis* suggest splitting the genus in two genera: *Nannocystis* (represented by *Nannocystis bainbridgea*, *Nannocystis exedens* (Type Species), *Nannocystis punicea*, *Nannocystis pusilla* and *Nannocystis radixulma)* and New Genus 1 (represented by *Nannocystis konarekensis).*

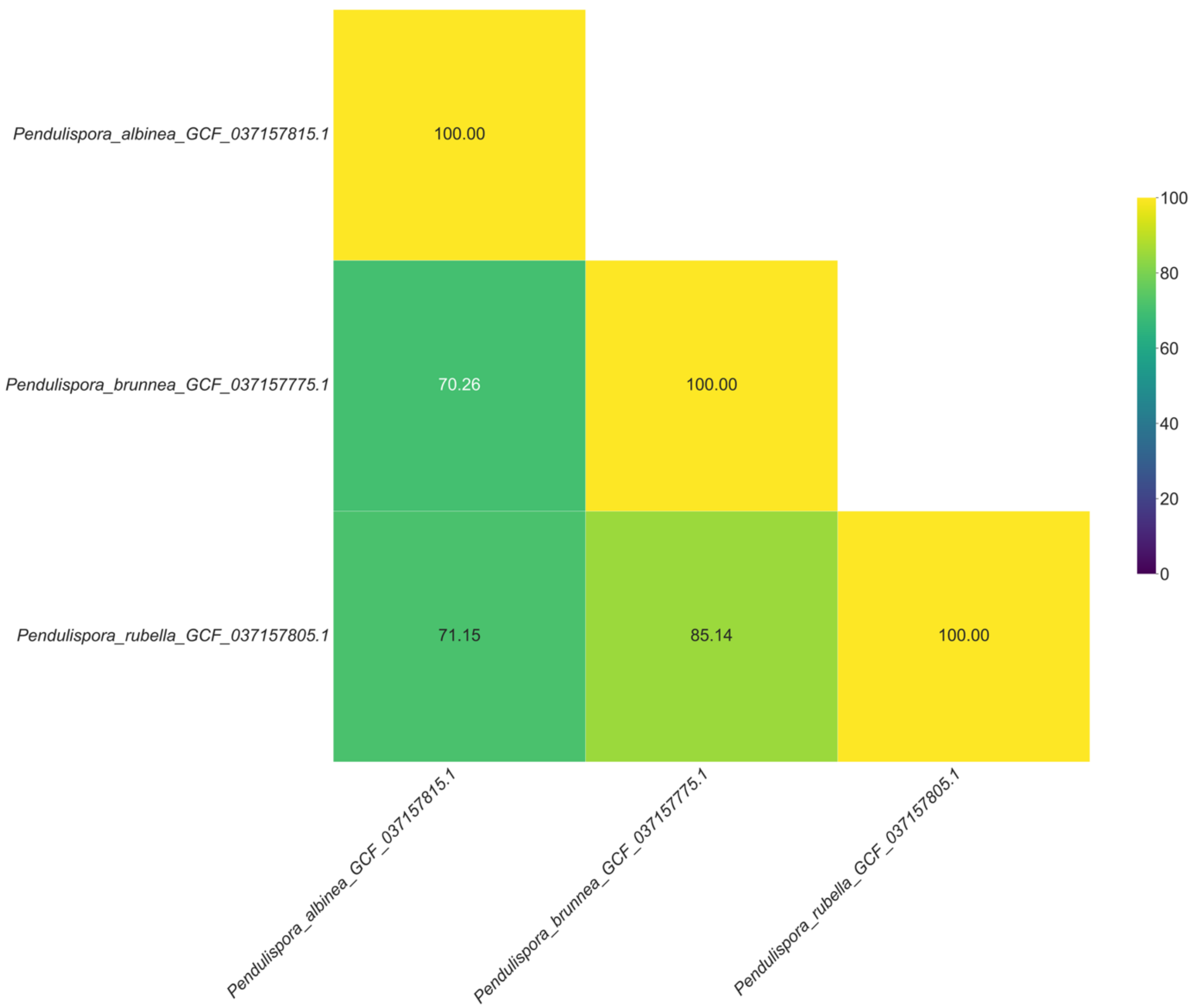

Supplementary Figure 14 The reclassified genus *Pendulispora.* The pairwise POCP values of species within *Pendulispora* suggest splitting the genus in two genera: *Pendulispora* (represented by *Pendulispora albinea* and New Genus 1 (represented by *Pendulispora brunnea* and *Pendulispora rubella).*

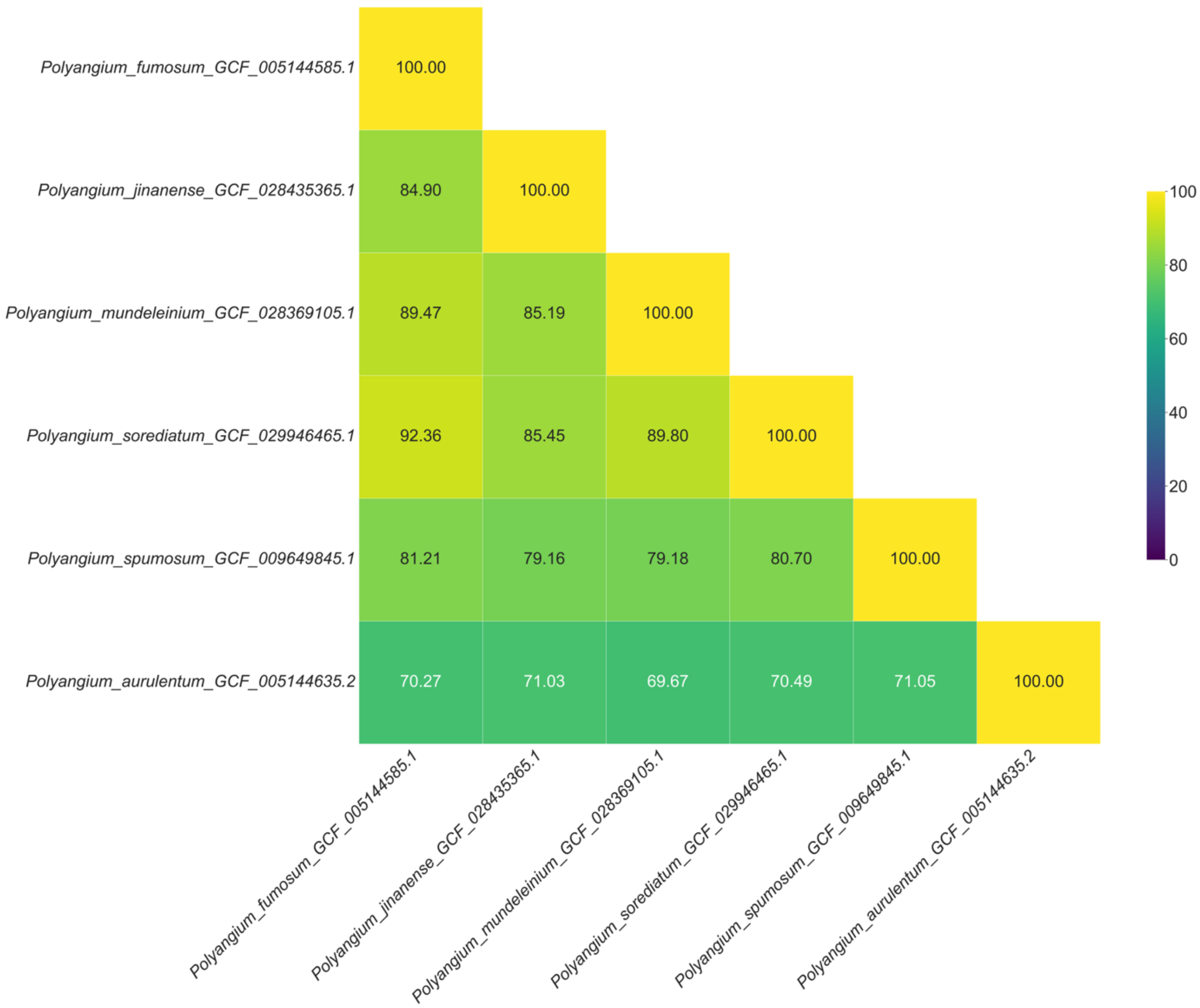

Supplementary Figure 15 The reclassified genus *Polyangium.* The pairwise POCP values of species within *Polyangium* suggest splitting the genus in two genera: *Polyangium* (represented by *Polyangium fumosum, Polyangium jinanense, Polyangium mundeleinium, Polyangium sorediatum, Polyangium spumosum* (Type species)) and New Genus 1 (represented by *Polyangium aurulentum).*

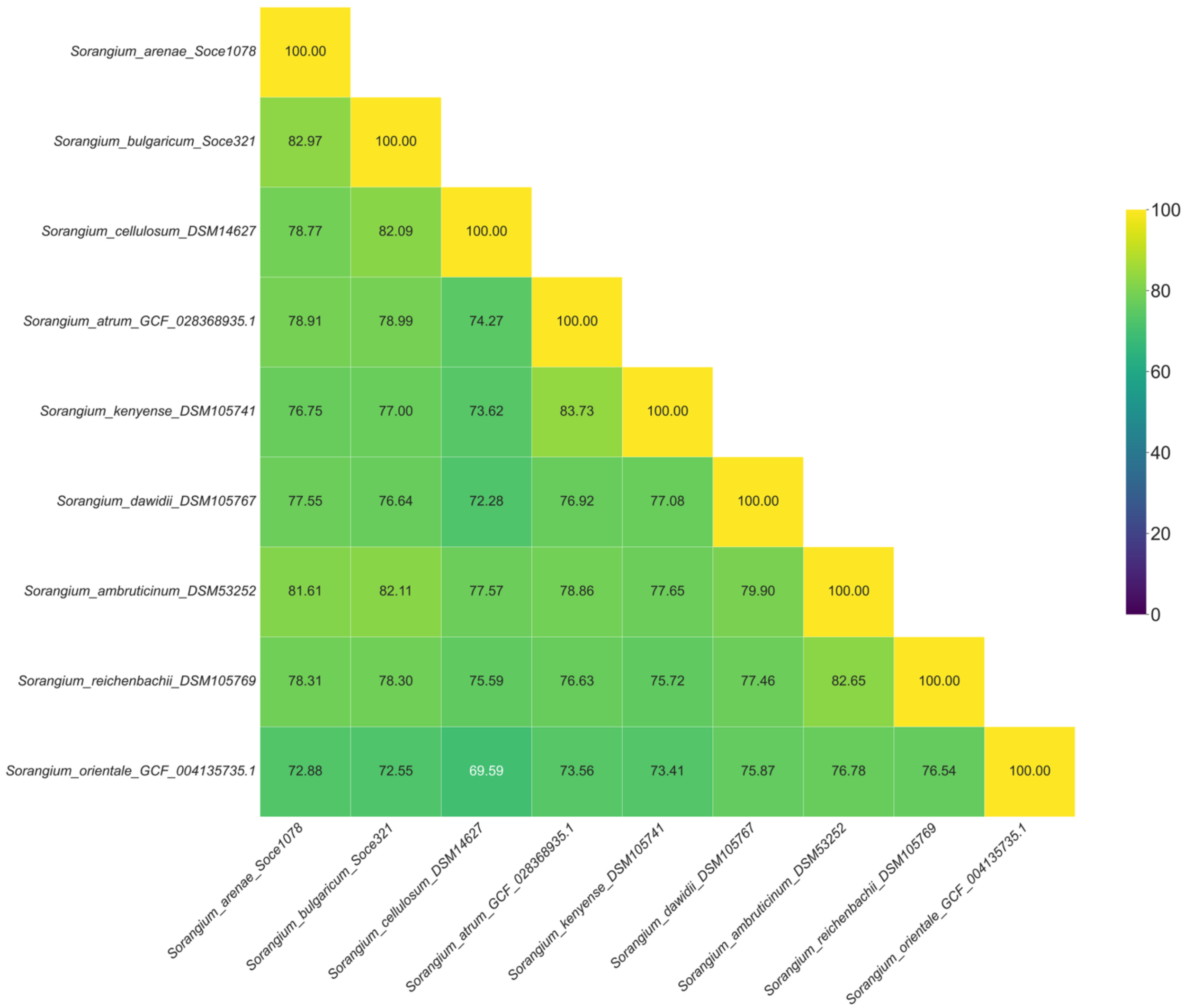

Supplementary Figure 16 The reclassified genus *Sorangium.* The pairwise POCP values of species within *Sorangium* suggest splitting the genus in four genera: *Sorangium* (represented by *Sorangium arenae, Sorangium bulgaricum, Sorangium cellulosum* (Type species)), New Genus 1 (represented by *Sorangium atrum, Sorangium kenyense),* New Genus 2 (represented by *Sorangium dawidii, Sorangium ambruticinum, Sorangium reichenbachii),* New Genus 3 (represented by *Sorangium orientale).*

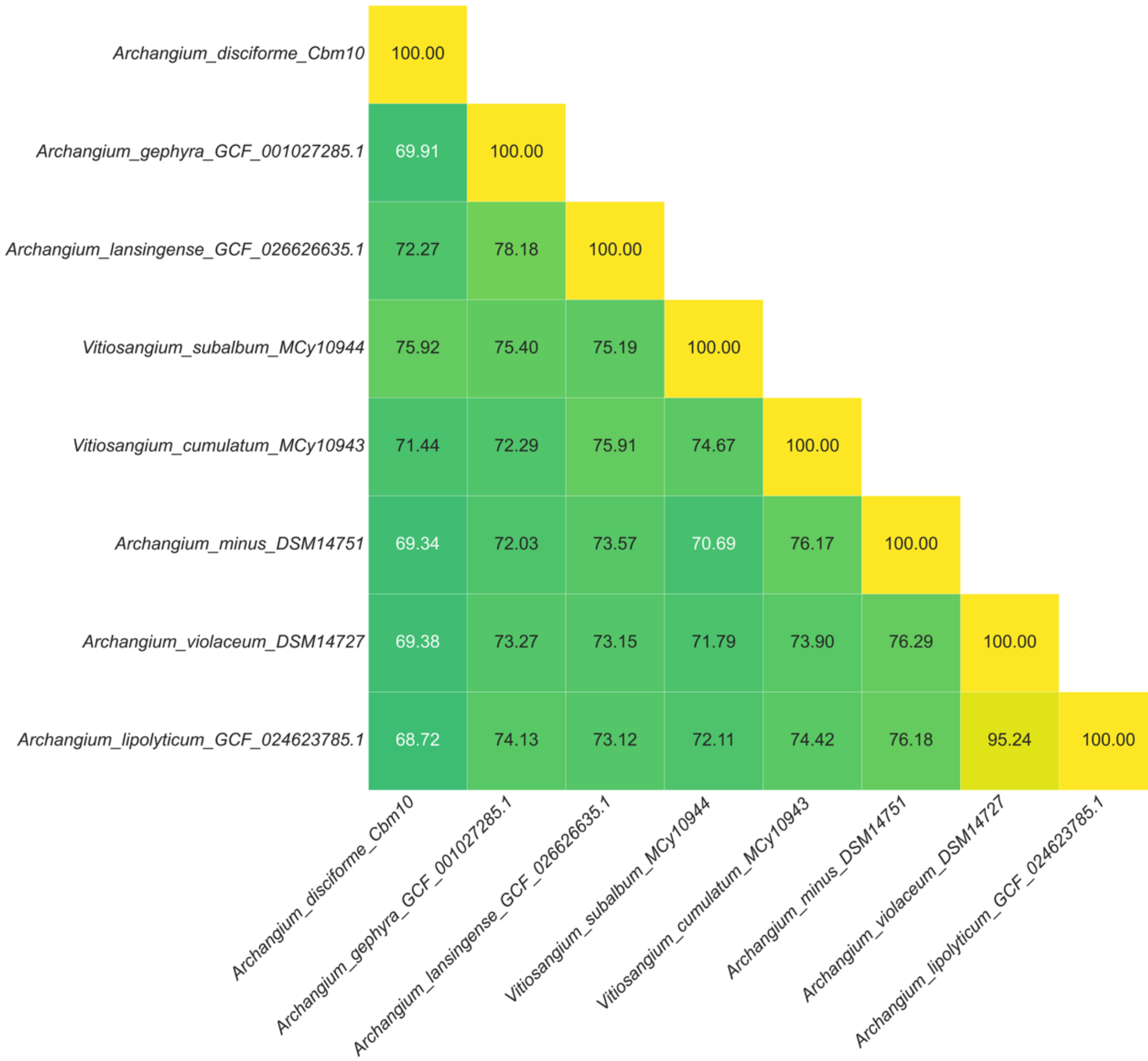

Supplementary Figure 17 The reclassified genera *Archangium* and *Vitiosangium.* The pairwise POCP values of species within *Archangium* and *Vitiosangium* suggest *Archangium gephyra* (Type species), *Archangium lansingense*, *Vitiosangium* subalbum (reclassify to *Archangium subalbum*) beong to the genus *Archangium*, while *Vitiosangium cumulatum* (Type species), *Archangium minus* (reclassify to *Vitiosangium minus*), *Archangium violaceum* (reclassify to *Vitiosangium violaceum*), *Archangium*. lipolyticum (reclassify to *Vitiosangium lipolyticum*) belong to the genus *Vitiosangium*.

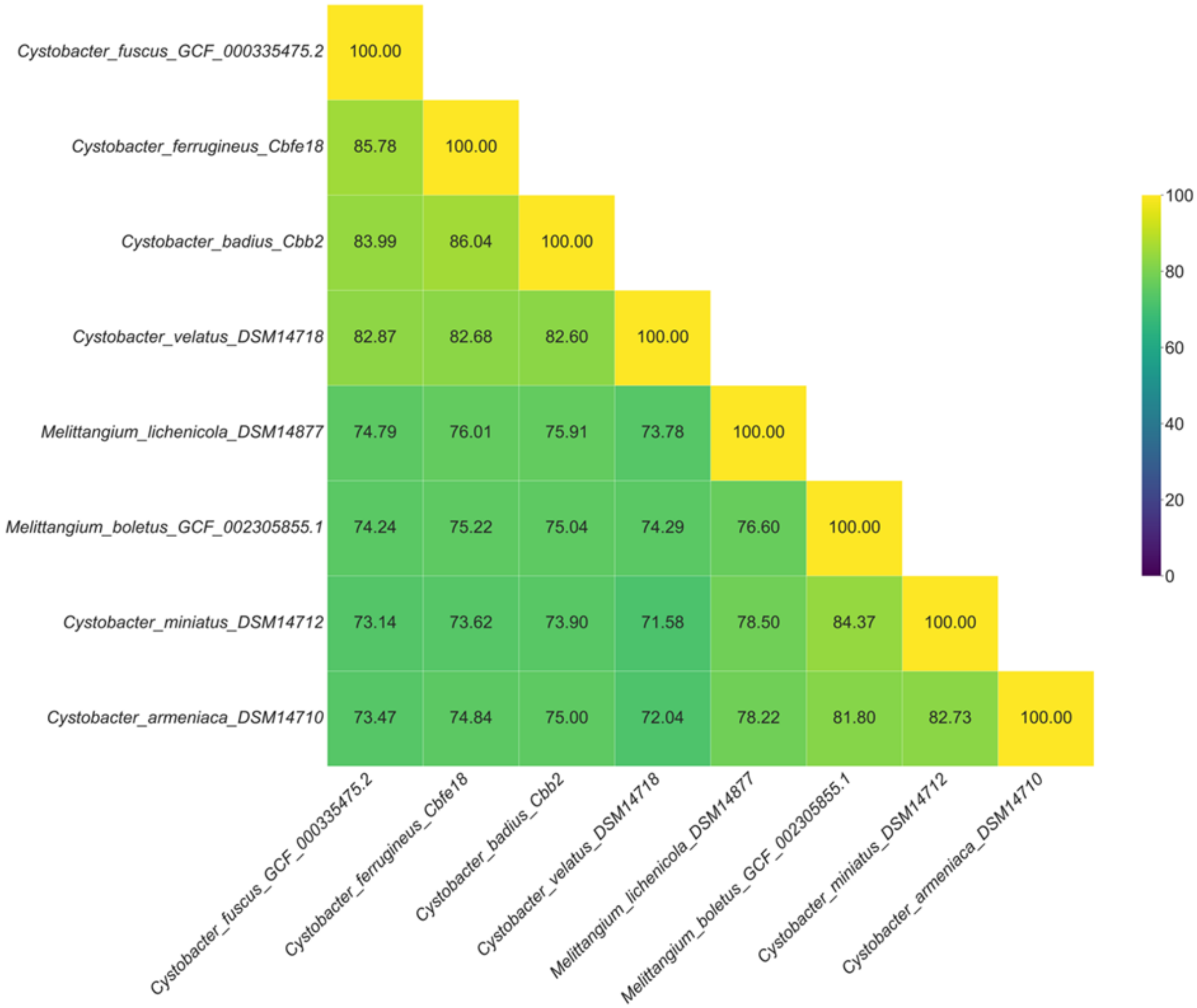

Supplementary Figure 18 The reclassified genera *Cystobacter* and *Melittangium*. The pairwise POCP values of species within *Cystobacter* and *Melittangium* suggest *Cystobacter fuscus* (Type species), *Cystobacter ferrugineus, Cystobacter badius* and *Cystobacter velatus* to belong to the genus Cystobacter, while *Melittangium lichenicola, Melittangium boletus* (Type species), *Cystobacter miniatus* (reclassified to *Melittangium miniatus*), *Cystobacter armeniaca* (reclassify to *Melittangium armeniaca*) and *Melittangium alboraceum* belong to the genus *Melittangium.*

Supplementary Note 2 New *Myxococcota* family proposal.

New Family 1: *Iridimyxaceae* fam. nov.

Iridimyxaceae (I.ri.di.my.xa.ce′ae. N.L. fem. n. *Iridimyxa*, type genus of a family; L. suff. –*aceae* ending to denote a family; N.L. fem. pl. n. *Iridimyxaceae* the family of the genus *Iridimyxa*).

New Family 2: *Saarlandiaceae* fam. nov.

Saarlandiaceae (Saar.lan.di.a.ce′ae. N.L. fem. n. *Saarlandia*, type genus of the family; L. suff. –*aceae* ending to denote a family; N.L. fem. pl. n. *Saarlandiaceae* the family of the genus *Saarlandia*).

New Family 3: *Tenuimyxaceae* fam. nov.

Tenuimyxaceae (Te.nu.i.my.xa.ce′ae. N.L. fem. n. *Tenuimyxa*, type genus of the family; L. suff. –*aceae* ending to denote a family; N.L. fem. pl. n. *Tenuimyxaceae* the family of the genus *Tenuimyxa*).

New Family 4: *Fluminibacteraceae* fam. nov.

Fluminibacteraceae (Flu.mi.ni.bac.ter.a.ce′ae. N.L. masc. n. *Fluminibacter*, type genus of the family; L. suff. –*aceae* ending to denote a family; N.L. fem. pl. n. *Fluminibacteraceae* the family of the genus *Fluminibacter*).

New Family 5: *Celerimyxobacteraceae* fam. nov.

Celerimyxobacteraceae (Ce.le.ri.my.xo.bac.te.ra.ce′ae. N.L. masc. n. *Celerimyxobacter*, type genus of the family; L. suff. –*aceae* ending to denote a family; N. L. fem. pl. n. *Celerimyxobacteraceae,* the family of the genus *Celerimyxobacter*).

New Family 6: *Subterraneimyxaceae* fam. nov.

Subterraneimyxaceae (Sub.ter.ra.ne.i.myx.a.ce′ae. N.L. fem. n. *Subterraneimyxa*, type genus of the family; L. suff. –*aceae* ending to denote a family; N.L. fem. pl. n. *Subterraneimyxaceae* the family of the genus *Subterraneimyxa*).

New Family 7: *Alimyxaceae* fam. nov.

Alimyxaceae (A.li.my.xa.ce′ae. N.L. fem. n. *Alimyxa*, type genus of the family; L. suff. –*aceae* ending to denote a family; N.L. fem. pl. n. *Alimyxaceae* the family of the genus *Alimyxa*).

New Family 8: *Plesiocystaceae* fam. nov.

Plesiocystaceae (Ple.si.o.cys.ta.ce′ae. N.L. fem. n. *Plesiocystis*, type genus of the family; L. suff. –*aceae* ending to denote a family; N.L. fem. pl. n. *Plesiocystaceae* the family of the genus *Plesiocystis*).

New Family 9: *Corallangiaceae* fam. nov.

Corallangiaceae (Co.rall.an.gi.a.ce′ae. N.L. neut. n. *Corallangium*, type genus of the family; L. suff. –*aceae* ending to denote a family; N. L. fem. pl. n. *Corallangiaceae* the family of the genus *Corallangium*).

New Family 10: *Pelagomyxaceae* fam. nov.

Pelagomyxaceae (Pe.la.go.my.xa.ce′ae. N.L. fem. n. *Pelagomyxa*, type genus of the family; L. suff. –*aceae* ending to denote a family; N.L. fem. pl. n. *Pelagomyxaceae* the family of the genus *Pelagomyxa*).

New Family 11: *Myxoflavaceae* fam. nov.

Myxoflavaceae (My.xo.fla.va.ce′ae. N.L. fem. n. *Myxoflava*, type genus of the family; L. suff. –*aceae* ending to denote a family; N. L. fem. pl. n. *Myxoflavaceae* the family of the genus *Myxoflava*).

New Family 12: *Scleromyxobacteraceae* fam. nov.

Scleromyxobacteraceae (Scle.ro.my.xo.bac.te.ra.ce′ae. N.L. masc. n. *Scleromyxobacter*, type genus of the family; L. suff. –*aceae* ending to denote a family; N.L. fem. pl. n. *Scleromyxobacteraceae* the family of the genus *Scleromyxobacter*).

New Family 13: *Limnomyxaceae* fam. nov.

Limnomyxaceae (Lim.no.my.xa.ce′ae. N.L. fem. n. *Limnomyxa*, type genus of the family; L. suff. –*aceae* ending to denote a family; N.L. fem. pl. n. *Limnomyxaceae* the family of the genus *Limnomyxa*).

New Family 14: *Cruentibacteraceae* fam. nov.

Cruentibacteraceae (Cru.en.ti.bac.ter.a.ce′ae. N.L. masc. n. *Cruentibacter*, type genus of the family; L. suff. –*aceae* ending to denote a family; N. L. fem. pl. n. *Cruentibacteraceae* the family of the genus *Cruentibacter*).

New Family 15: *Saxicystaceae* fam. nov.

Saxicystaceae (Sa.xi.cys.ta.ce′ae. N.L. fem. n. *Saxicystis*, type genus of the family; L. suff. –*aceae* ending to denote a family; N. L. fem. pl. n. *Saxicystaceae* the family of the genus *Saxicystis*).

New Family 16: *Versibacteraceae* fam.nov.

Versibacteraceae (Ver.si.bac.te.ra.ce′ae. N.L. masc. n. *Versibacter*, type genus of the family; L. suff. –*aceae* ending to denote a family; N. L. fem. pl. n. *Versibacteraceae* the family of the genus *Versibacter*).

New Family 17: *Filimyxobacteraceae* fam. nov.

Filimyxobacteraceae (Fi.li.my.xo.bac.te.ra.ce′ae. N.L. masc. n. *Filimyxobacter*, type genus of the family; L. suff. –*aceae* ending to denote a family; N. L. fem. pl. n. *Filimyxobacteraceae* the family of the genus *Filimyxobacter*).

New Family 18: *Halomyxaceae* fam. nov.

Halomyxaceae (Ha.lo.my.xa.ce′ae. N.L. fem. n. *Halomyxa*, type genus of the family; L. suff. –*aceae* ending to denote a family; N. L. fem. pl. n. *Halomyxaceae* the family of the genus *Halomyxa*).

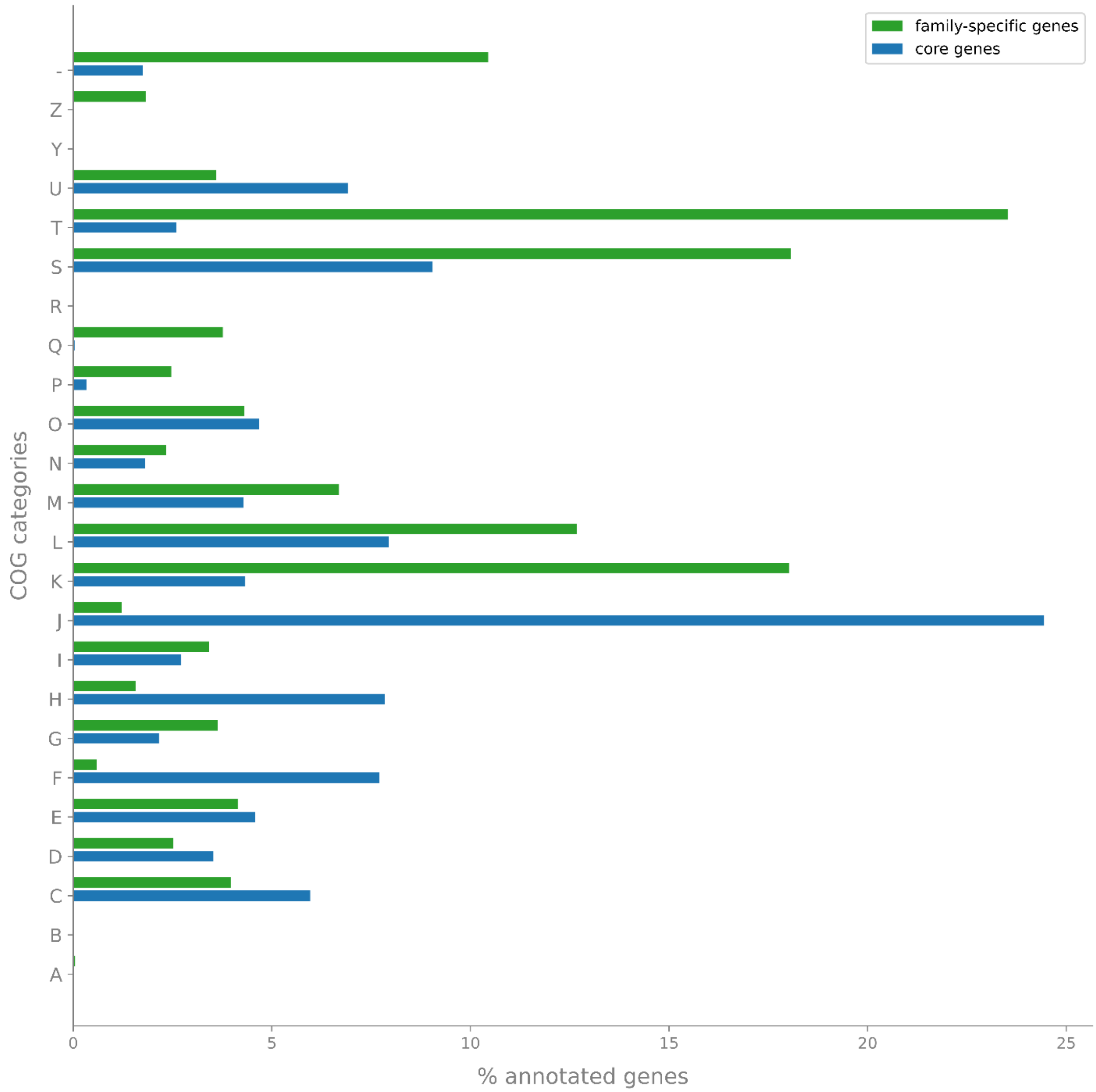

Supplementary Figure 19 Distribution of COG (Clusters of Orthologous Groups) categories among core genes (blue) and family-specific genes (green). Core genes are shared by all 153 myxobacterial genomes. The y-axis shows for each COG category on the x-axis its percentage of the annotated genes across the family-specific genes or the core genes. COG-categories:

J	Translation, ribosomal structure and biogenesis
A	RNA processing and modification
K	Transcription
L	Replication, recombination and repair
B	Chromatin structure and dynamics
D	Cell cycle control, cell division, chromosome partitioning
Y	Nuclear structure
V	Defense mechanisms
T	Signal transduction mechanisms
M	Cell wall/membrane/envelope biogenesis

N	Cell motility
Z	Cytoskeleton
W	Extracellular structures
U	Intracellular trafficking, secretion, and vesicular transport
O	Posttranslational modification, protein turnover, chaperones
X	Mobilome: prophages, transposons
C	Energy production and conversion
G	Carbohydrate transport and metabolism
E	Amino acid transport and metabolism
F	Nucleotide transport and metabolism
H	Coenzyme transport and metabolism
I	Lipid transport and metabolism
P	Inorganic ion transport and metabolism
Q	Secondary metabolites biosynthesis, transport and catabolism
R	General function prediction only
S	Function unknown
-	No annotation

Supplementary Note 3 Structure elucidation of myxolutamid A and identification of the putative *mxl* BGC.

HRESIMS measurements revealed an $[M+H]^+$ peak for myxolutamid A at *m/z* 779.5400 (Supplementary Figure 22) corresponding to the molecular formula $C_{39}H_{71}N_8O_8$ (*m/z* calcd for $[M+H]^+$ 779.5389) featuring 9 double-bond equivalents (DBE). ^{1}H-NMR and HSQC experiments in DMSO-d_6 (Supplementary Figure 21 and 23) display characteristic signals for an octapeptid with eight α-proton signals (Supplementary Table 5). Combination of COSY and HMBC correlations (Supplementary Figure 22 and 24) reveal the octapeptide to consist of five leucines as well as three alanines and the amino acid sequence was elucidated as Leu-Leu-Leu-Ala-Ala-Leu-Leu-Ala via HMBC correlations from the respective α-protons to the adjacent carbonyl groups and confirmed by the fragmentation sequence observed in the MS^2 spectra (Supplementary Figure 26). Noteworthy, the α-proton of Leu-1 does not show a correlation to the carbonyl of Leu-2, yet, the established connections between the other amino acids only allow ring closure in this position. Marfey's analysis (Supplementary Figure 28 and 29) was used to confirm an all-D configuration for the comprised amino acids.

Pendulispora MSr11367 contains 10 NRPS and 14 NRPS hybrid biosynthetic gene cluster predicted with antiSMASH 8.0[4]. Only in cluster 1.35, the predicted A-domain specificity fits the amino acid composition of myxolutamid A, which is why we propose this BGC to encode for the biosynthetic machinery involved in myxolutamid formation. The nearest Stachelhaus code[5] match of the A-domain in module 1, 3 and 4 is Leu or 3-OH Leu with their sequences showing 91, 91 and 77 % similarities, respectively. Only module 2 is predicted to incorporate alanine with the nearest Stachelhaus code match of the A-domain at 94 % similarity. Interestingly, the NRPS core gene only contains four A-domains which would require at least two modules in the BGC to act repetitively for forming the full peptide before release by the TE in module 4 (Supplementary Figure 30). The amino acid order determined by NMR and MS^2 indicates the *mxi*BGC as non-linear NRPS (Type C) rather than an iterative one (Type B[6]). Additionally, the esterase domain in module 3 seems to act *in trans* on the other modules to generate the all-D configuration observed in myxolutamid A, which is also consistent with the C-domains in module 2, 3, and 4 predicted as $^{D}C_{L}$ domains[7].

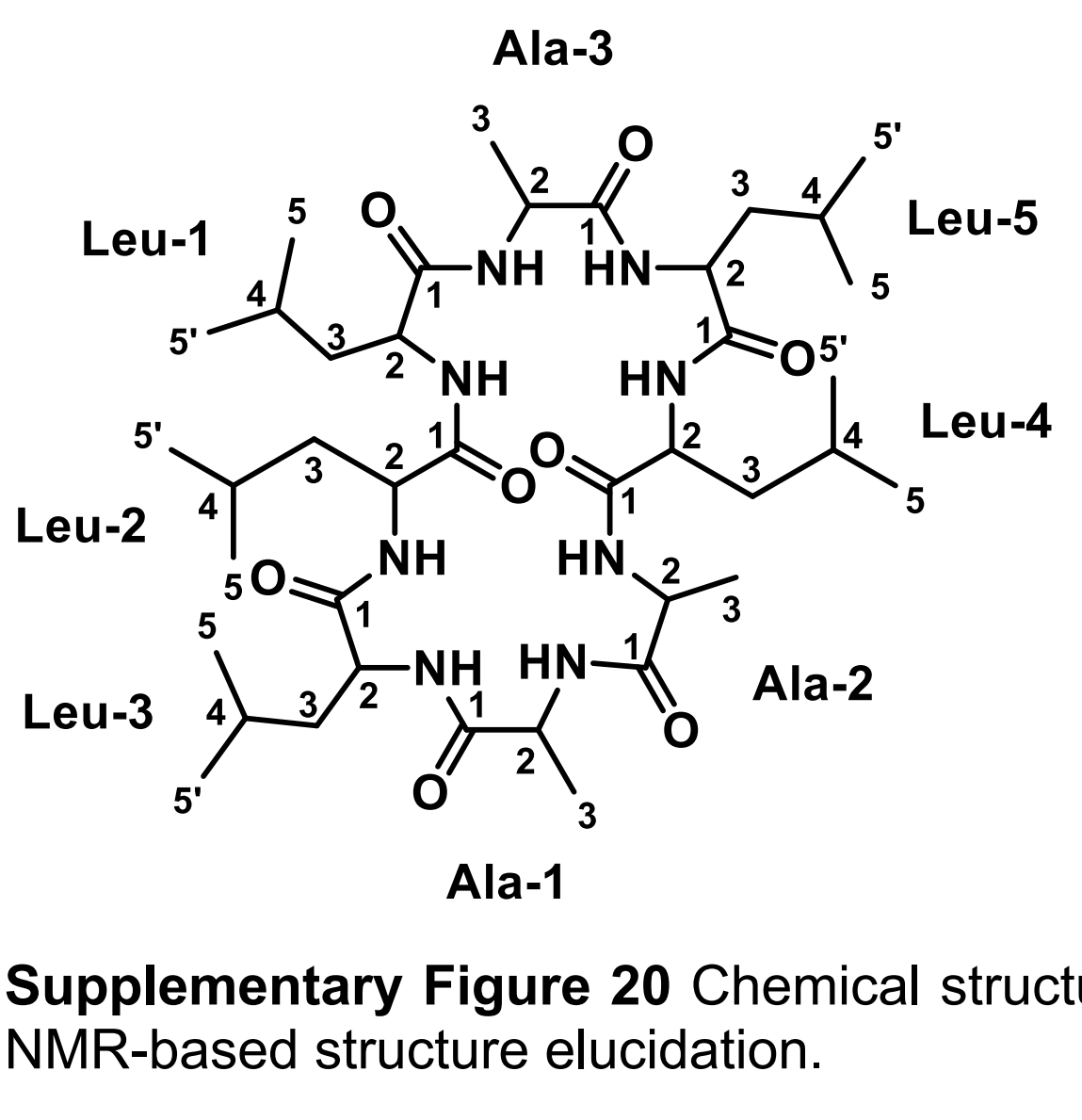

Supplementary Figure 20 Chemical structure of myxolutamid A with atom numbering used for NMR-based structure elucidation.

Supplementary Table 5 NMR spectroscopic data of myxolutamid A in DMSO-d6 at 500/125 MHz.

#	δ ^{13}C [ppm]	δ ^{1}H [ppm], mult (*J* [Hz])	COSY	HMBC
**Leu-1**				
1	175.1	-	-	-
2	54.7	4.19, m	3, NH	1, 3, 4
3	39.6	1.48, 1.73, m	2, 4	2, 4,
4	24.0	1.86, m	3, 5'	3, 5'
5	20.5	0.93, d*	3, 4, 5'	3, 4, 5'
5'	22.8	1.01, d (6.26)	4, 5	3, 4, 5
NH	-	9.02, bd	2	-
**Leu-2**				
1	172.0	-	-	-
2	52.5	4.12, m	3, NH	1, Leu-3 1
3	39.3	1.48, 1.90, m	2, 4	2, 4, 5, 5'
4	24.1	1.69, m	3, 5, 5'	3, 5, 5'
5	21.4	0.96, d (6.56)	3, 4, 5'	3, 4, 5'
5'	23.1	1.02, d (6.64)	4, 5	3, 4, 5
NH	-	7.54, bd	2	-
**Leu-3**				
1	171.3	-	-	-
2	50.4	4.32, m	3, 4, NH	1, 3, 4, NH, Ala-1 1
3	38.4	1.69, 1.79, m	2, 4, 5, 5'	2, 4, 5, 5'
4	22.9	1.66, m	3, 5, 5'	2, 3, 5
5	20.6	0.91, d (6.50)	3, 4, 5'	3, 4, 5'
5'	22.8	0.99, d (6.71)	4, 5	3, 4, 5
NH	-	7.63, d (7.71)	2	Ala-1 1
**Ala-1**				
1	172.7	-	-	-
2	51.7	3.89, m	3, NH	1, 3
3	15.8	1.38, d (7.30)	2	1, 2
NH	-	8.71, bd	2	1, Ala-2 1
**Ala-2**				
1	173.6	-	-	-
2	48.5	4.50, m	3, NH	1, 3, Leu-4 1
3	15.5	1.23, d (6.33)	2	1, 2
NH	-	7.40, bs	2	Leu-4 1
**Leu-4**				
1	171.3	-	-	-
2	48.6	4.78, m	3, NH	1, 3
3	40.8	1.81, 1.54, m	2, 4, 5, 5'	2, 4, 5, 5'
4	23.9	1.58, m	3, 5, 5'	-
5	20.3	0.90, d (6.41)	3, 4, 5'	3, 4, 5'
5'	22.8	1.01, d (6.26)	4, 5	3, 4, 5
NH	-	7.14, bd (9.40)	2	Leu-5 1
**Leu-5**				
1	171.6	-	-	-
2	49.6	4.37, m	3	1, 3
3	39.9	1.69, 1.75, m	2, 4, 5, 5'	2, 5, 5'
4	23.8	1.81, m*	3, 5, 5'	-
5	20.6	0.94, d*	3, 4, 5'	3, 4, 5'

5’	23.3	0.99, d (5.34)	3, 4, 5’	3, 4, 5
NH	-	7.59, bd (9.99)	2	Ala-3 1
**Ala-3**				
1	173.0	-	-	-
2	50.8	4.09, m	3, NH	1, 3, Leu-1 1
3	16.1	1.40, d (7.50)	2	1, 2
NH	-	8.74, bd	2	-

* no assignment possible due to overlapping signals

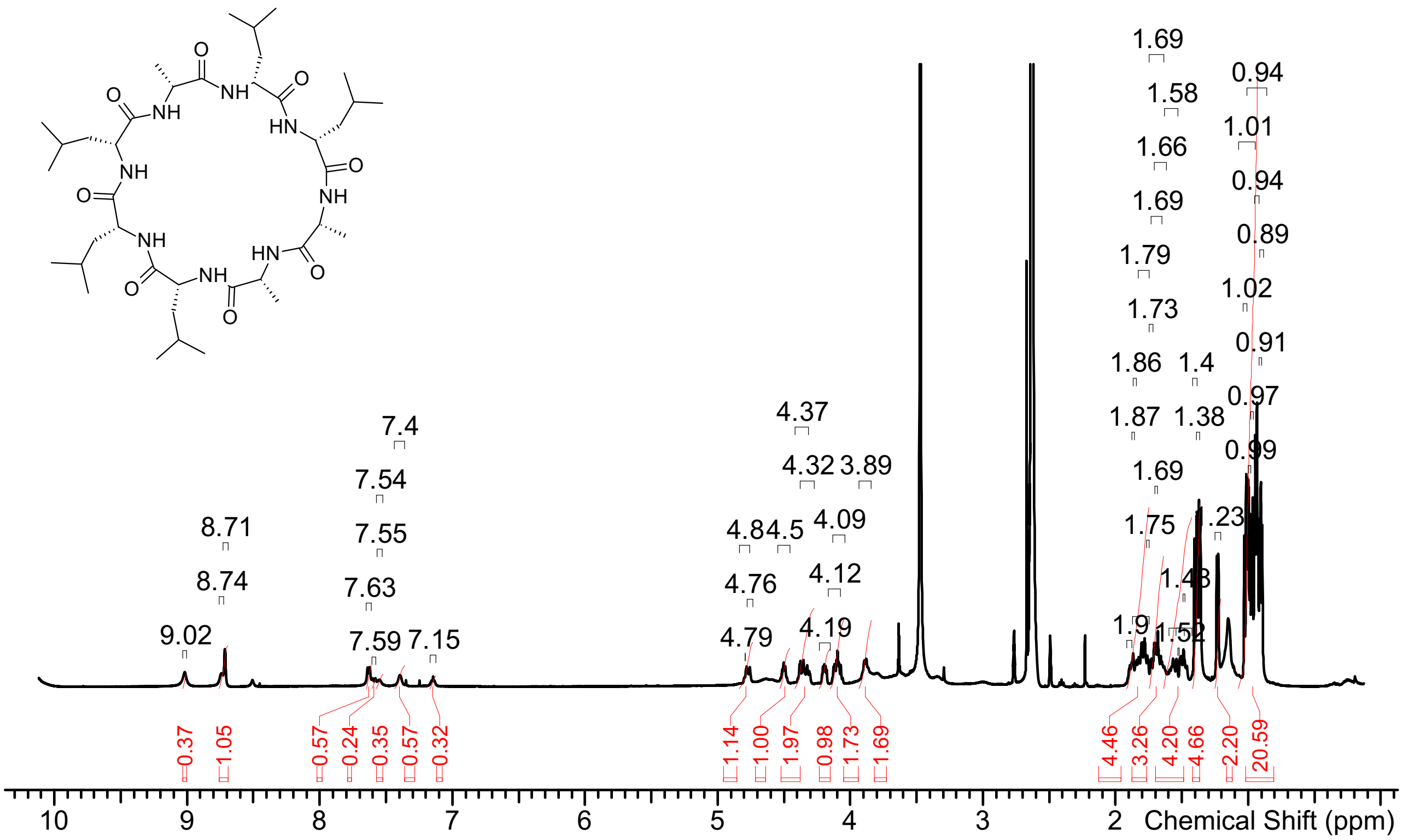

Supplementary Figure 21 ^{1}H-spectrum of myxolutamid A in DMSO-d_6 at 500 MHz.

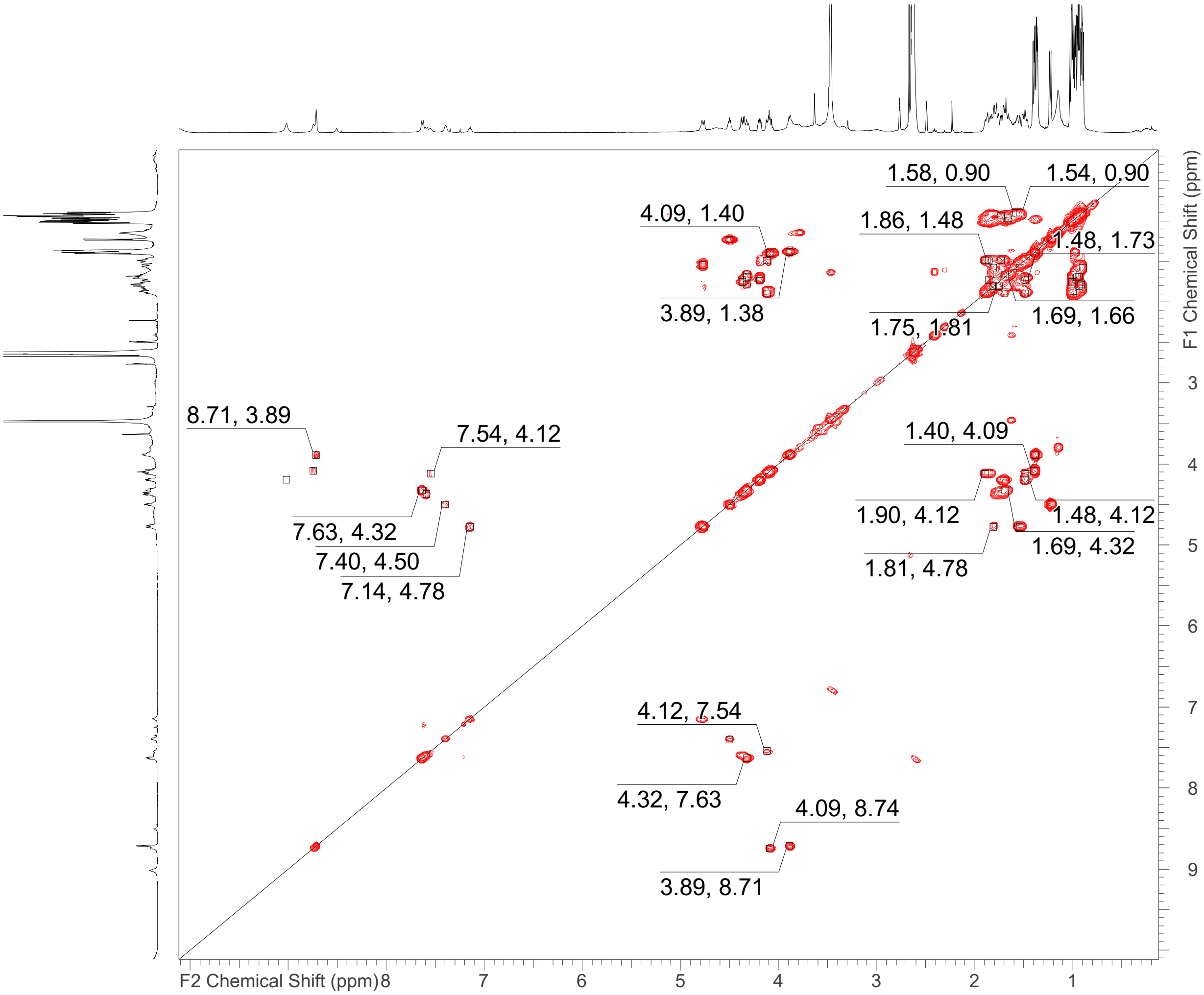

Supplementary Figure 22 COSY-spectrum of myxolutamid A in DMSO-d_6 at 500 MHz.

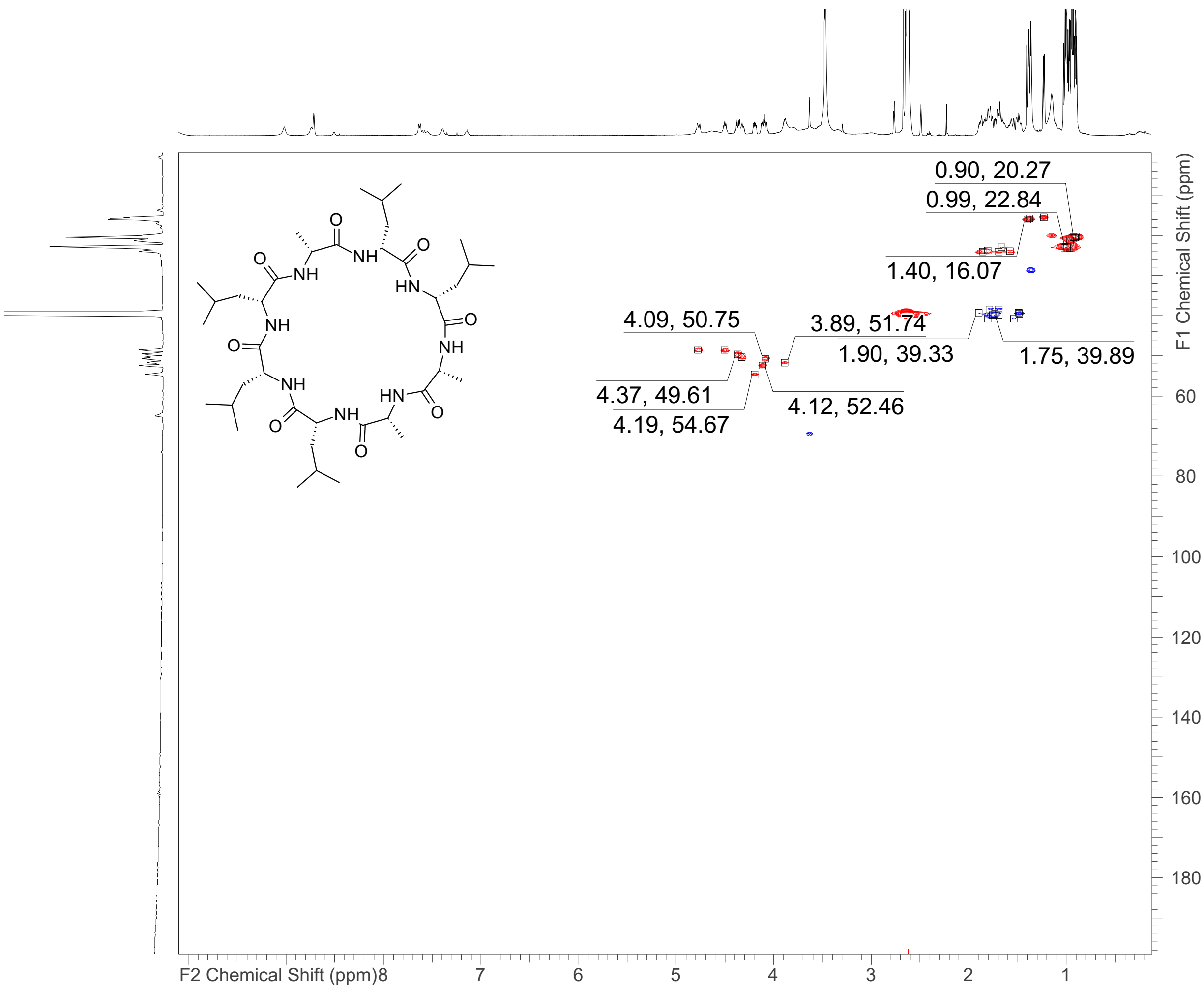

Supplementary Figure 23 HSQC-spectrum of myxolutamid A in DMSO-d_6 at 500 MHz/125 MHz.

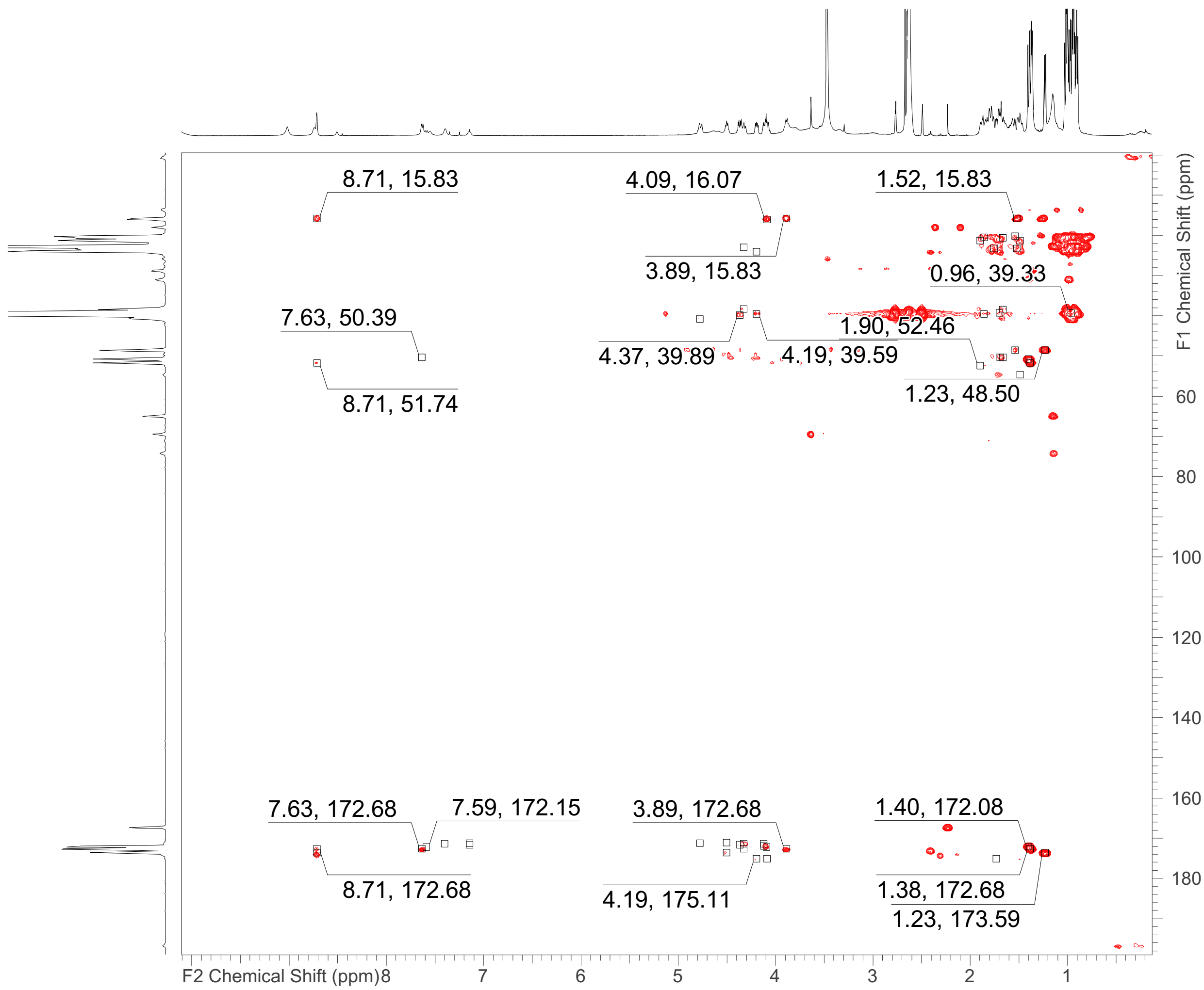

Supplementary Figure 24 HMBC-spectrum of myxolutamid A in DMSO-d6 at 500 MHz/125 MHz.

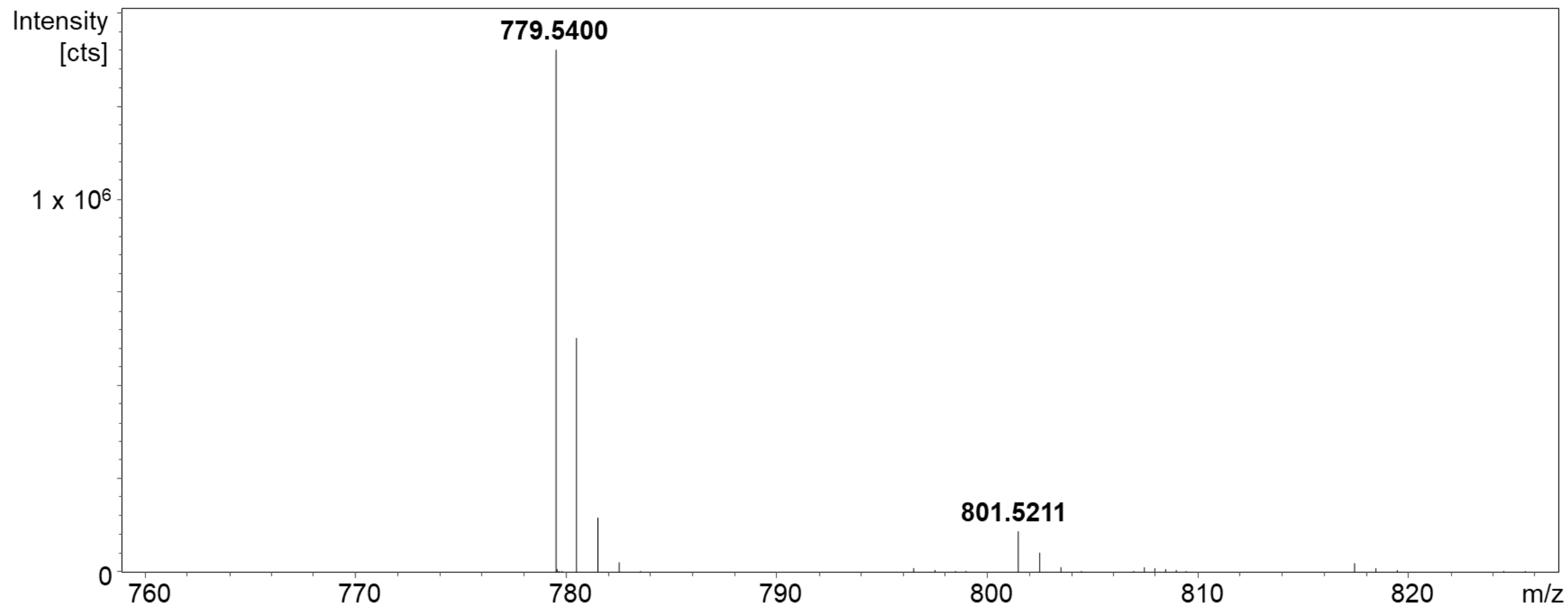

Supplementary Figure 25 MS spectrum for myxolutamid A showing $[M+H]^+$ at *m/z* 779.5400 and $[M+Na]^+$ at *m/z* 801.5211 as most prominent ion types.

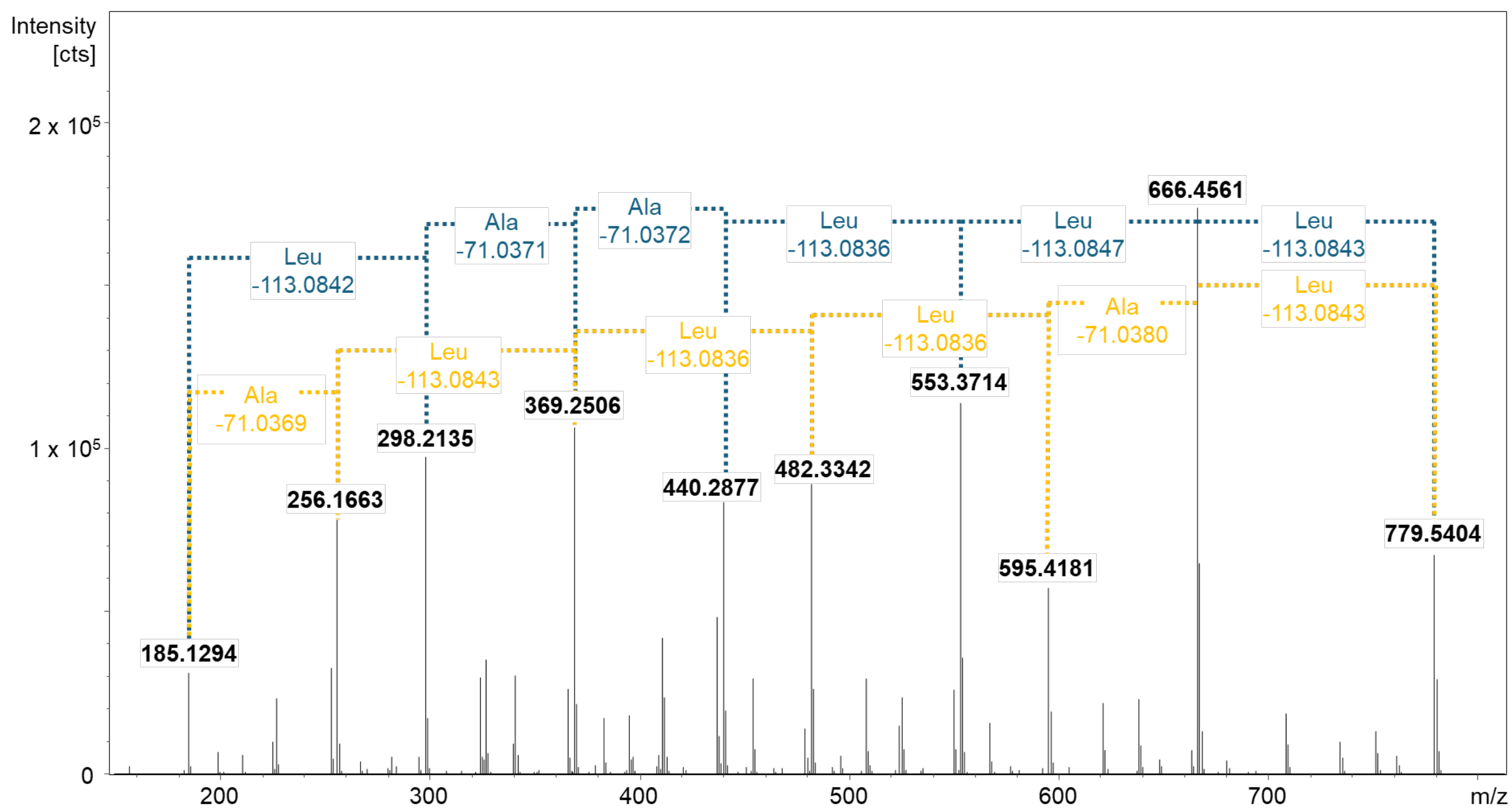

Supplementary Figure 26 MS^2 fragment spectrum for myxolutamid A showing sequential losses of leucine and alanine in myxolutamid A.

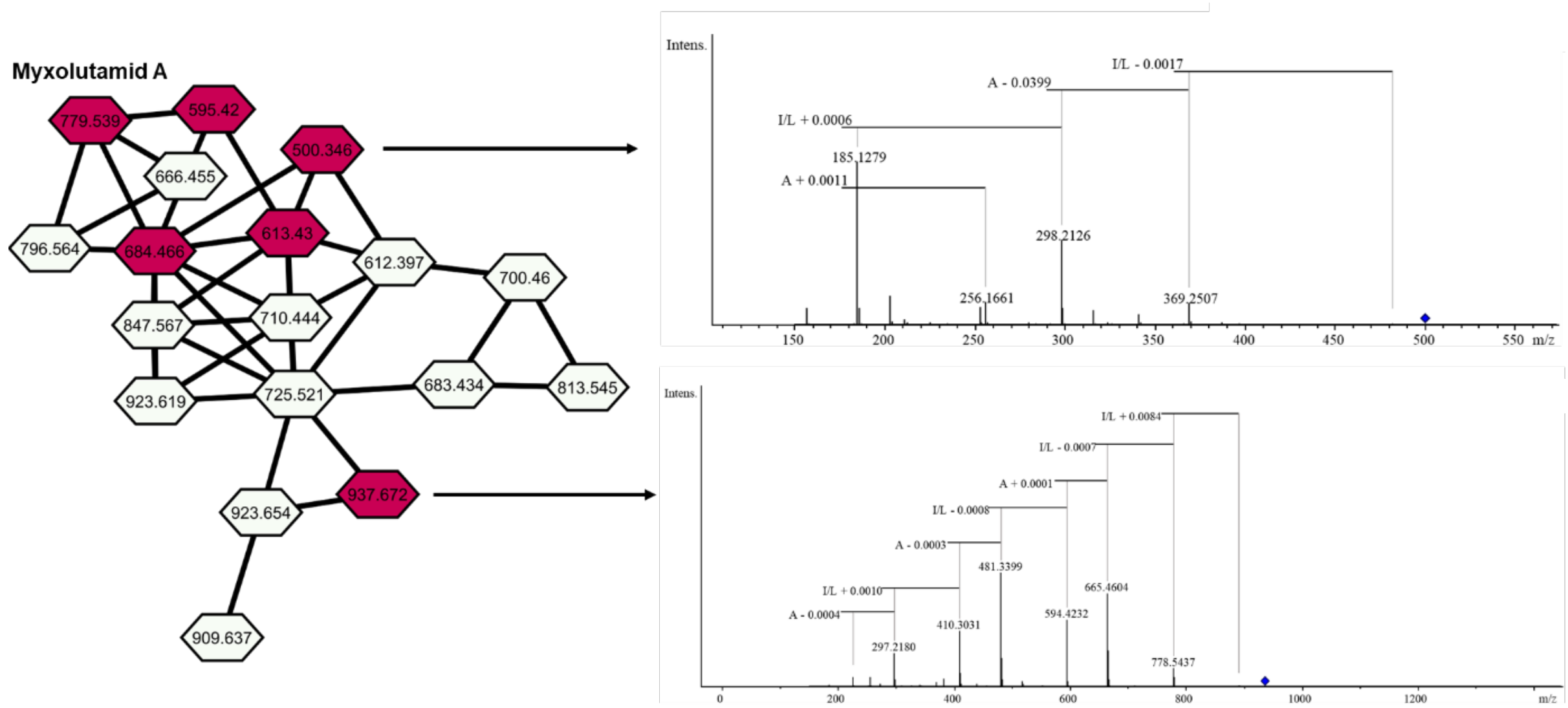

Supplementary Figure 27 Myxolutamid molecular network generated in GNPS and MS^2 spectra of selected family members showing sequential losses of leucine and alanine. The myxolutamid derivative at $[M+H]^+$ shows an m/z at 595.4190 for $[M+H]^+$ in line with the sum formula $C_{30}H_{55}N_6O_6^+$ (*m/z* calcd for $[M+H]^+$ 595.4178 Δ2.1 ppm), indicating a six membered cyclic structure reduced by one leucine and one alanine in comparison to myxolutamid A. We also observe the corresponding ring opened analogues through water addition at *m/z* 684.4658 as $[M+H]^+$ with the molecular formula $C_{33}H_{62}N_7O_8^+$ (*m/z* calcd for $[M+H]^+$ 684.4654 Δ0.57 ppm) as well as at m/z 613.4297 as $[M+H]^+$ matching the sum formula $C_{30}H_{57}N_6O_7^+$ (*m/z* calcd for $[M+H]^+$ 595.4178 Δ2.1 ppm).

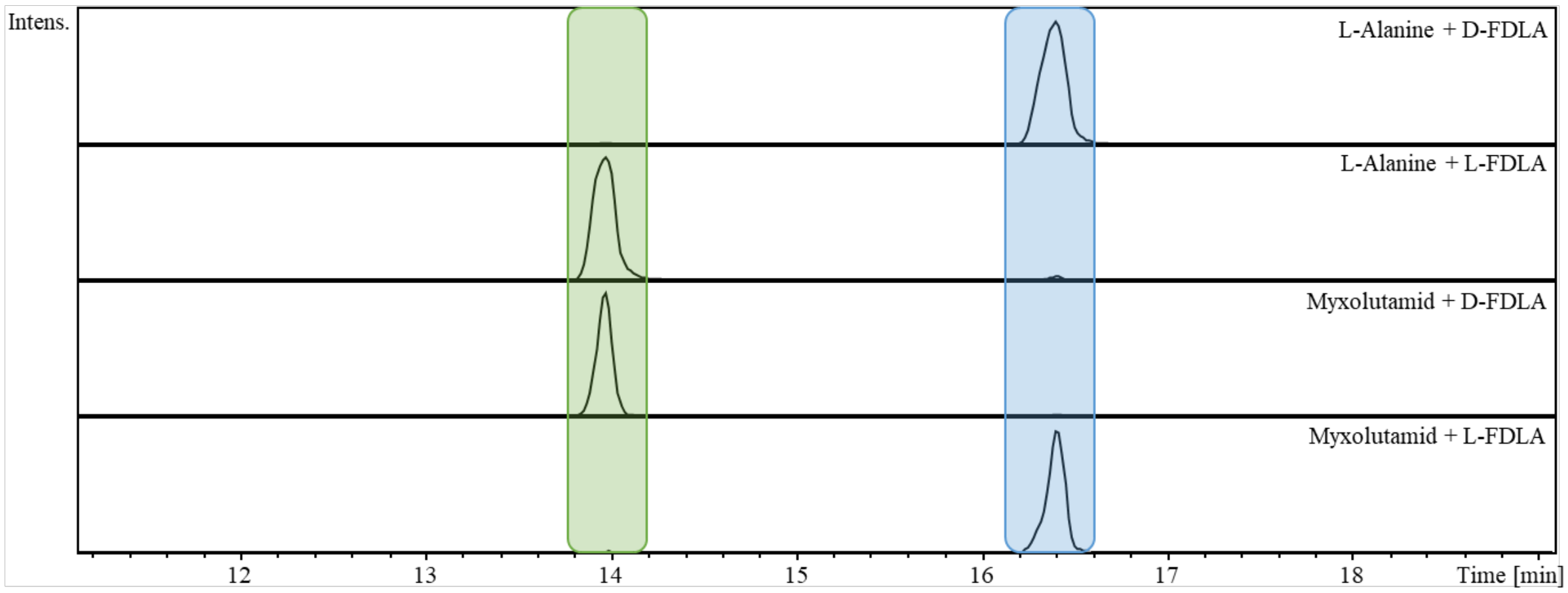

Supplementary Figure 28 Marfey's derivatization of reference L-alanine with D-FDLA and L-FDLA (upper 2 chromatograms) and myxolutamid A with D-FDLA and L-FDLA (lower 2 chromatograms) for retention time comparison resulted in assignment of D-alanine incorporation in myxolutamid A.

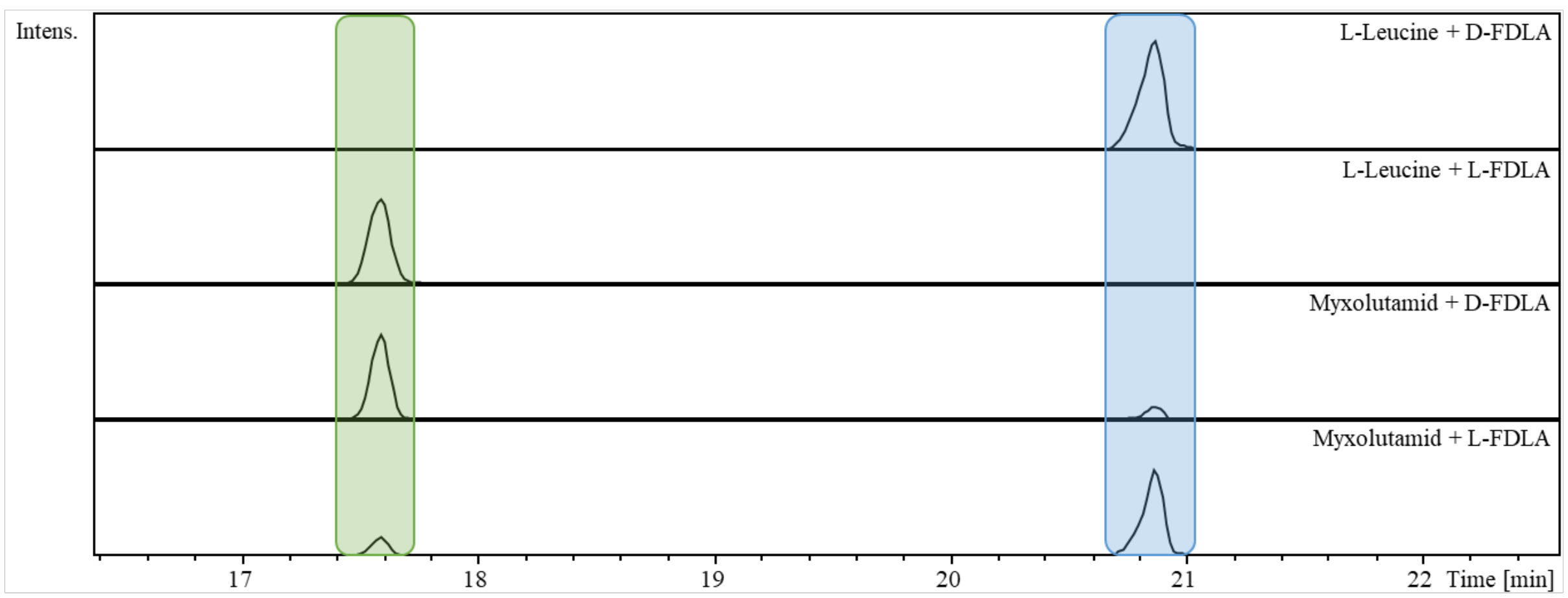

Supplementary Figure 29 Marfey's derivatization of reference L-leucine with D-FDLA and L-FDLA (upper 2 chromatograms) and myxolutamid A with D-FDLA and L-FDLA (lower 2 chromatograms) for retention time comparison resulted in assignment of D-leucine incorporation in myxolutamid A.

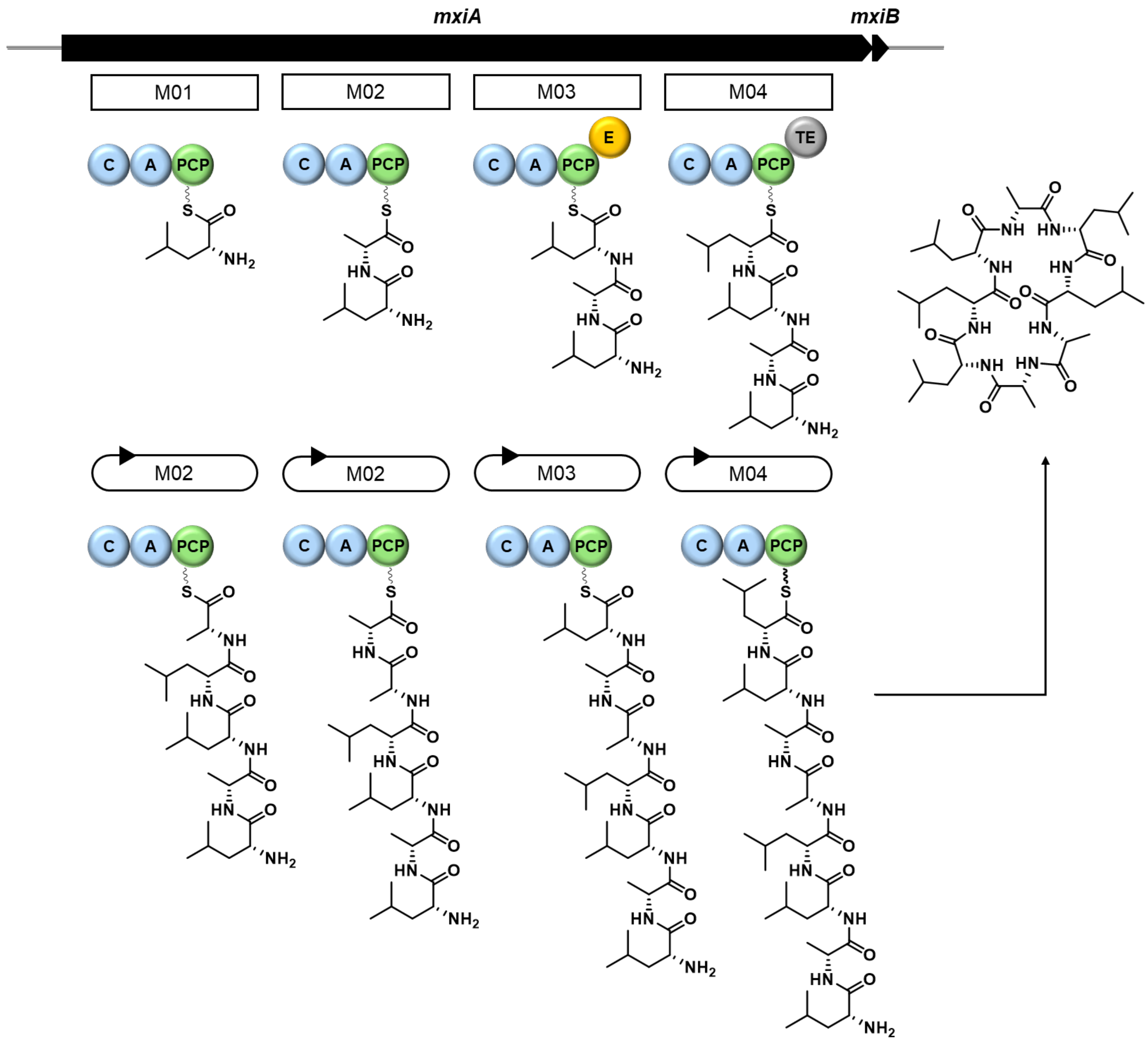

Supplementary Figure 30 *mxi*BGC from *Pendulispora* MSr11367 putatively involved in formation of myxolutamid A. The BGC is proposed to be a non-linear NRPS with Module 2 acting repetitively twice after a first pass through the assembly line (marked with circular arrows) before the growing peptide chain is passed again to module 3 and 4 and released by the TE of module 4. M= module; C = condensation domain; A = adenylation domain; PCP = peptide carrier protein domain; E = esterase domain; TE = thioesterase domain.

Supplementary Table 6 Open reading frames in the putative *mxl* BGC from *Pendulispora* MSr11367 with proposed function and closest homologues.

Name	Size [AA]	Proposed function/ homologue	Organism	Accession number of closest protein homologue	Sequence identity [%]
MxiA	4906	ATP-dependent valine adenylase	*Brevibacillus parabrevis*	Q70LM5.1	39%
MxiB	89	Amino acid activation/ Protein MbtH	*Mycobacterium tuberculosis*	P59965.1	62%

Supplementary Note 4 Structure elucidation of sorangicin X and Y.

HRESIMS data of sorangicin X (Supplementary Figure 44) showed an $[M-H_2O+H]^+$ signal at *m/z* 783.4472, corresponding to a molecular sum formula of $C_{48}H_{63}O_9$ (calc. 783.4467; Δ 0.6 ppm), which contains 18 double-bond equivalents (DBEs). The chemical structure of sorangicin X was elucidated from 1D and 2D NMR data in methanol-d_4 (Supplementary Table 7). It differs from the reported sorangicin A [8,9] in several parts of the large macrolactone moiety (Supplementary Figure 32). Firstly, in accordance with the recently reported sorangicin P[10], sorangicin X features an additional triene-moiety at C-15 to C-20. The positions of the two additional olefinic protons H-17 and H-18 were determined by COSY correlations with the adjacent methines ($\delta_{H\text{-}16}$ 6.04, $\delta_{H\text{-}17}$ 6.32, $\delta_{H\text{-}18}$ 6.11, $\delta_{H\text{-}19}$ 6.25). Another difference in sorangicin X is the methyl-residue C-46 ($\delta_{C\text{-}46}$ 9.0, $\delta_{H\text{-}46}$ 0.8) bound to C-22, replacing the hydroxyl-residue bound to the respective carbon in sorangicin A. Moreover, sorangicin X features a ketone ($\delta_{C\text{-}25}$ 213.0) that is absent in sorangicin A. Its position was established based on HMBC correlations of the surrounding protons ($\delta_{H\text{-}23}$ 3.14, $\delta_{H\text{-}24a}$ 2.46, $\delta_{H\text{-}24a}$ 2.29, $\delta_{H\text{-}26}$ 2.14, $\delta_{H\text{-}27}$ 3.38). The configuration of *Z* and *E* double bonds was determined by their vicinal coupling constants of < 12 Hz and 14-15 Hz, respectively. This analysis yielded similar results and configurations for all double bonds that are shared with sorangicin A and point towards a *Z* configuration of the additional double bond at C-17 and C-18. The high structural similarity between sorangicin X and sorangicin A is further reinforced by a high resemblance of the respective δ_C and δ_H (Supplementary Table 8, 9). Some shift differences could be caused by a change in the ring tension of the macrolactone through the additional double bond at C-17 to C-18 as well as slight changes in the conformation caused by the substitution of two hydroxyl-residues in sorangicin A by the methyl-residue at C-22 and the ketone at C-25 in sorangicin X. The absence of the hydroxyl-residue at C-22 in sorangicin X might be also the reason for the surprisingly large differences in the chemical shifts of the sidechain, as it was hypothesized to be stabilized within the macrolactone ring by a hydrogen bond bridge between the hydroxyl-residue at C-22 and the carboxylic acid of C-1 in sorangicin A[8].

Sorangicin Y (Supplementary Figure 45) shows a HRESIMS $[M-H_2O+H]^+$ signal at *m/z* 785.4637 (calc. 785.4624 Δ = 1.7 ppm), corresponding to the molecular sum formula of $C_{48}H_{65}O_9$ that has an unsaturation degree of 17 DBEs. Its structure was elucidated from 1D and 2D NMR data in methanol-d_4 (Supplementary Figure 33, Supplementary Table 10) and shows a high similarity to the structure of sorangicin X. However, unlike sorangicin X, it lacks the signal of the ketone C-25 at 213.0 ppm. Instead, the HSQC spectrum features a further hydroxylated methine ($\delta_{C\text{-}25}$ 71.2, $\delta_{H\text{-}25}$ 3.79), which position was established based on clear COSY correlations with the adjacent methylene and methine ($\delta_{H\text{-}24a}$ 1.67, $\delta_{H\text{-}24a}$ 1.48, $\delta_{H\text{-}26}$ 1.50).

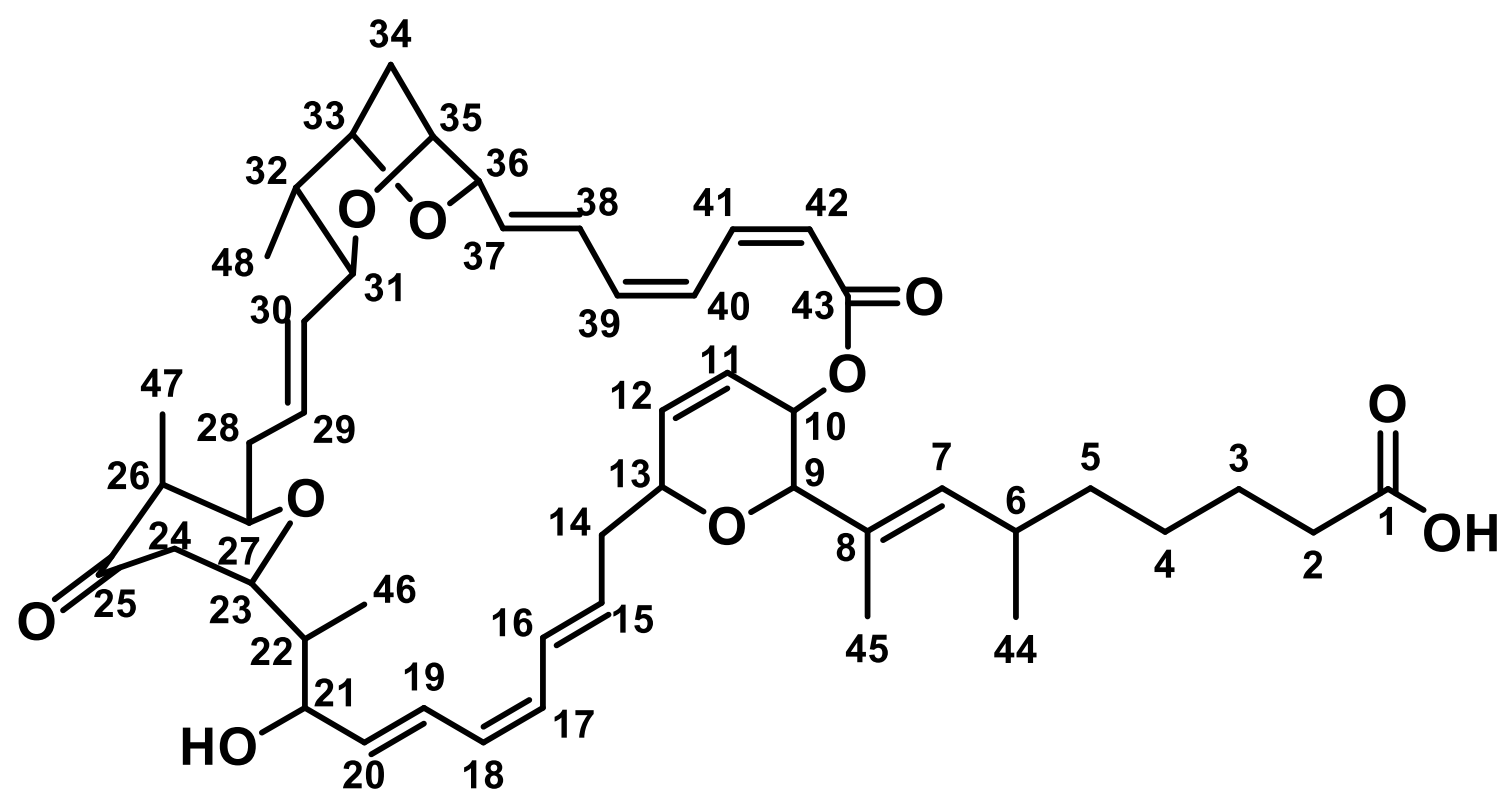

Supplementary Figure 31 Chemical structure of sorangicin X with atom numbering used for NMR-based structure elucidation.

Supplementary Table 7 NMR spectroscopic data of sorangicin X in methanol-d_4 at 500/125 MHz.

#	δ ^{13}C [ppm]	δ ^{1}H [ppm], mult (*J* [Hz])	COSY	HMBC
1	177.6			
2	35.8	2.19, td (7.6, 7.6, 1.2)	3	1, 3, 4
3	26.3	1.47, m	2, 4, 5a	1, 2, 4, 5
4	28.5	1.13, m	3, 5a, 5b	2, 3, 5
5a	39.4	1.08, m	3, 4	4, 6, 7
5b		0.86, m	4, 6	3, 4, 6, 7
6	32.6	2.27, m	5b, 7, 44	3, 7, 8
7	133.1	5.15, d (10.1)	6, 9, 45	5, 6, 8, 9, 44, 45
8	129.8			
9	73.4	4.06, s	7, 10, 45	5, 7, 8, 10, 13, 45
10	65.7	5.45, m	9, 11	9, 11, 12, 43
11	124.5	6.09, m	10, 12, 13	9, 10, 13
12	136.9	6.16, dd (9.9, 2.8)	11, 13	10, 11, 13
13	75.0	4.52, m	11, 12, 14a, 14b	9
14a	35.7	2.44, m	13, 15	13, 16
14b		2.26, m	13, 15, 16	13, 15
15	134.7	5.84, ddd (14.9, 10.4, 4.3)	14a, 14b, 16	13, 14, 16, 17
16	133.7	6.04, br dd (15.2, 11.1)	14b, 15, 17	14, 17, 18, 19
17	135.7	6.32, m	16, 18	15, 18
18	130.8	6.11, m	17, 19	16, 17, 19, 20
19	135.6	6.25, dd (15.1, 10.8)	18, 20	17, 18, 21
20	130.8	5.65, br dd (15.0, 9.3)	19, 21	18, 19, 21, 22
21	73.9	4.53, m	20, 22	19, 20, 22, 23, 46
22	45.0	2.00, m	21, 23, 46	20, 21, 23, 24, 46
23	81.1	3.14, m	22, 24a, 24b	21, 22, 25, 27, 46
24a	43.2	2.46, m	23, 27	22, 23, 25, 26
24b		2.29, m	23	25, 26
25	213.0			
26	48.8	2.14, m	27, 47	24, 25, 28, 47
27	78.7	3.38, m	24a, 26, 28	23, 25, 28, 29, 47
28	35.7	2.40, m	27, 29	26, 27, 29, 30
29	130.2	5.27, ddd (15.1, 10.7, 4.1)	28, 30	27, 28, 30, 31
30	134.6	5.46, m	29, 31	28, 29, 32
31	80.9	3.80, t (9.1, 9.1)	30, 32	29, 30, 32, 33, 48

32	41.9	1.41, m	31, 33, 48	30, 31, 33, 34, 48
33	81.0	4.30, m	32, 34a, 34b	31, 34, 35, 48
34a	39.5	2.03, m	33, 35	33, 36
34b		1.92, br dd (11.5, 1.1)	33, 35	32, 33, 35, 36, 37
35	77.4	4.40, m	34a, 34b, 36	31, 33, 36
36	81.4	4.60, m	35, 37, 38, 40	
37	135.5	6.30, m	36, 38	35, 36, 38, 39
38	128.1	7.05, m	36, 37, 39	36, 39, 40
39	137.9	6.45, br t (11.1, 11.1)	38, 40, 42	37, 38, 41
40	127.3	7.26, m	36, 39, 41, 42	38, 41, 42
41	139.3	7.11, m	40, 42	39, 40, 42, 43
42	119.5	5.54, br d (11.4)	39, 40, 41	40, 41, 43
43	167.9			
44	22.4	0.69, br d (6.6)	6	3, 4, 5, 6, 7, 8
45	14.8	1.54, s	7, 9	5, 7, 8, 9
46	9.0	0.88, br d (7.0)	22	21, 22, 23
47	10.3	1.06, br d (7.2)	26	25, 26, 27
48	15.4	0.76, br d (6.7)	32	31, 33

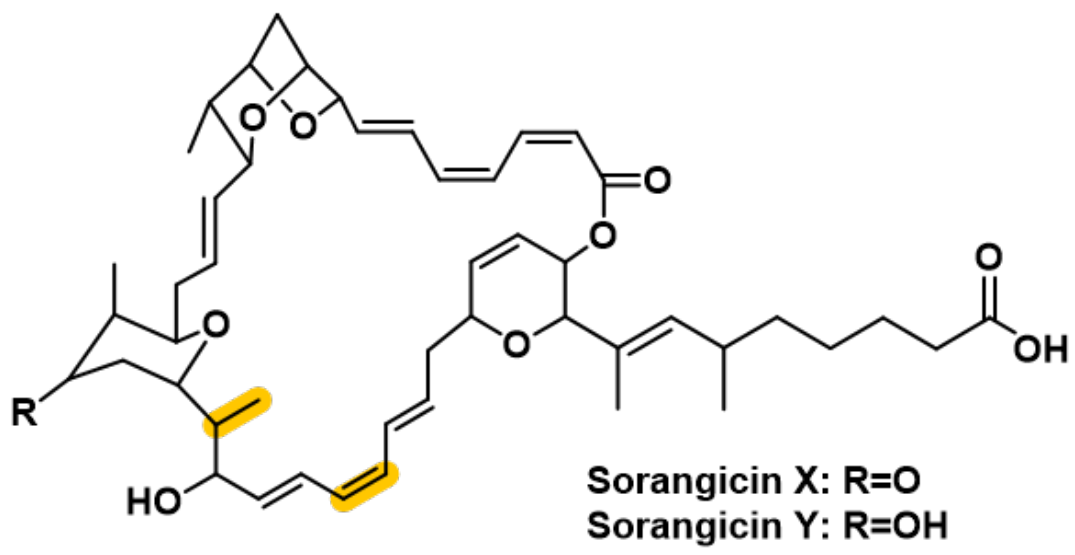

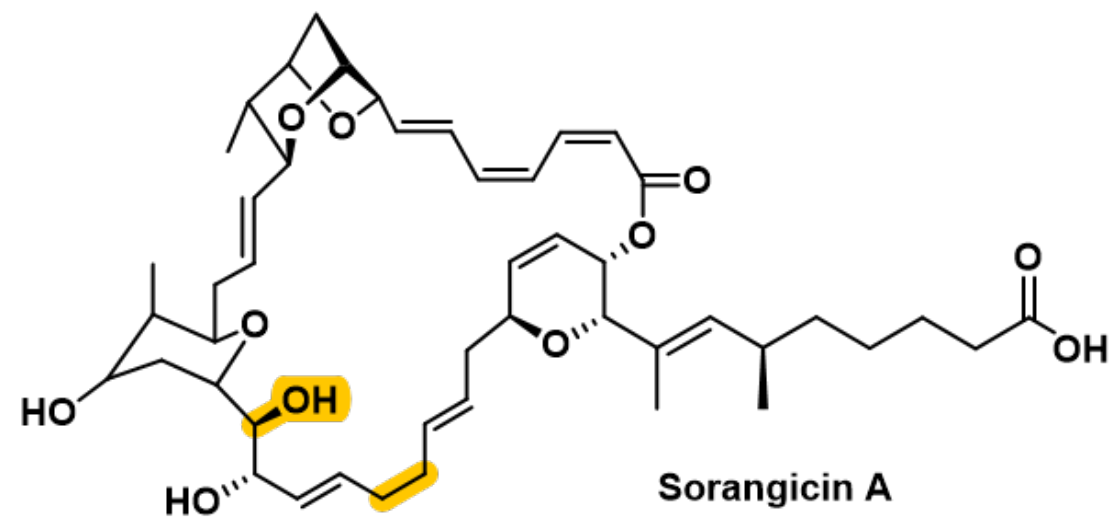

Supplementary Figure 32 Structural comparison of sorangicins X and Y with sorangicin A. Differences are highlighted in yellow.

Supplementary Table 8 ^{1}H NMR comparison of sorangicin X and sorangicin A in methanol-d_4.

#	δ ^{1}H [ppm] sorangicin X	#	δ ^{1}H [ppm] sorangicin A	Δ (δ ^{1}H sorangicin X - δ ^{1}H sorangicin A)
1		1		0.00
2	2.19	2	2.3	-0.11
3	1.47	3	1.62	-0.16
4a	1.13	4a	1.36	-0.23
4b	1.08	4b	1.3	-0.22
5a	1.08	5a	1.42	-0.34
5b	0.86	5b	1.25	-0.39
6	2.27	6	2.43	-0.16
7	5.15	7	5.34	-0.19
8	-	8	-	-
9	4.06	9	4.28	-0.22
10	5.45	10	5.35	0.10
11	6.09	11	6.05	0.04
12	6.16	12	6.17	-0.01
13	4.52	13	4.43	0.09
14a	2.44	14a	2.43	0.01
14b	2.26	14b	2.17	0.09
15	5.84	15	5.58	0.26
16	6.04	16	5.58	0.46
17	6.32	-	-	-
18	6.11	-	-	-
-	-	17a	2.24	-
-	-	17b	2.14	-
-	-	18a	2.24	-
-	-	18b	2.17	-
19	6.25	19	5.79	0.46
20	5.65	20	5.64	0.01
21	4.53	21	4.19	0.34
22	2.00	22	3.52	-1.52
23	3.14	23	3.73	-0.59
24a	2.46	24a	1.76	0.70
24b	2.29	24b	1.7	0.59

25	-	25	3.87	-
26	2.14	26	1.59	0.55
27	3.38	27	3.89	-0.51
28a	2.40	28a	2.32	0.08
28b	2.40	28b	2.17	0.23
29	5.27	29	5.54	-0.27
30	5.46	30	5.42	0.04
31	3.80	31	3.87	-0.07
32	1.41	32	1.46	-0.05
33	4.30	33	4.32	-0.02
34a	2.03	34a	2.09	-0.06
34b	1.92	34b	1.97	-0.05
35	4.40	35	4.45	-0.05
36	4.60	36	4.61	-0.02
37	6.30	37	6.26	0.04
38	7.05	38	7.03	0.02
39	6.45	39	6.48	-0.03
40	7.26	40	7.24	0.02
41	7.11	41	7.19	-0.08
42	5.54	42	5.66	-0.12
43	-	43	-	-
44	0.69	44	0.93	-0.25
45	1.54	45	1.68	-0.14
46	0.88	-	-	-
47	1.06	46	0.93	0.13
48	0.76	47	0.86	-0.11

Supplementary Table 9 ^{13}C NMR comparison of sorangicin X and sorangicin A in methanol-d$_4$.

#	δ ^{13}C [ppm] sorangicin X	#	δ ^{13}C [ppm] sorangicin A	Δ (δ ^{13}C sorangicin X - δ ^{13}C sorangicin A)
1	177.6	1	177.9	-0.3
2	35.8	2	35.3	0.5
3	26.3	3	26.3	0.1
4	28.5	4	28.2	0.3
5	39.4	5	38.5	0.9
6	32.6	6	33.0	-0.3
7	133.1	7	134.2	-1.1
8	129.8	8	131.2	-1.5
9	73.4	9	74.4	-0.9
10	65.7	10	66.9	-1.2
11	124.5	11	123.8	0.7
12	136.9	12	136.9	0.1
13	75.0	13	75.3	-0.3
14	35.7	14	35.5	0.3
15	134.7	15	128.3	6.4
16	133.7	16	133.6	0.1
17	135.7	17	33.4	102.4
18	130.8	18	34.0	96.8
19	135.6	19	134.4	1.3
20	130.8	20	130.2	0.7
21	73.9	21	74.4	-0.6
22	45.0	22	77.8	-32.8
23	81.1	23	75.1	6.1
24	43.2	24	30.9	12.4
25	213.0	25	71.1	141.9
26	48.8	26	38.5	10.3
27	78.7	27	74.9	3.8
28	35.7	28	37.1	-1.5
29	130.2	29	133.0	-2.8
30	134.6	30	132.8	1.8
31	80.9	31	81.2	-0.3
32	41.9	32	42.2	-0.3
33	81.0	33	81.0	0.0
34	39.5	34	39.9	-0.4
35	77.4	35	77.6	-0.1
36	81.4	36	82.3	-0.9
37	135.5	37	134.9	0.6
38	128.1	38	127.8	0.2
39	137.9	39	137.6	0.3
40	127.3	40	127.0	0.3
41	139.3	41	139.1	0.2
42	119.5	42	119.7	-0.2

43	167.9	43	167.7	0.2
44	22.4	44	21.7	0.7
45	14.8	45	14.3	0.5
46	9.0	-	-	-
47	10.3	46	10.9	-0.6
48	15.4	47	15.4	0.0

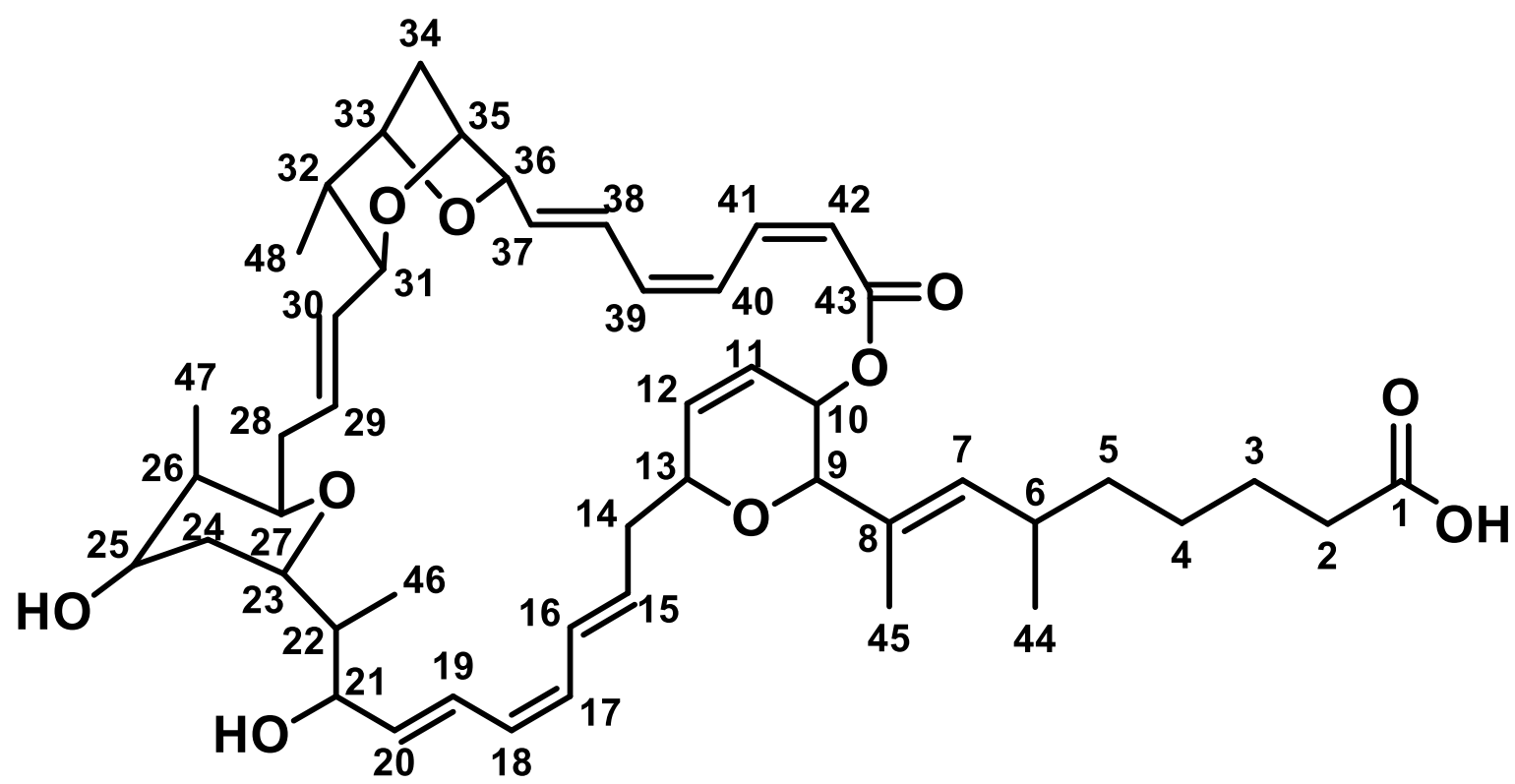

Supplementary Figure 33 Chemical structure of sorangicin Y with atom numbering used for NMR-based structure elucidation.

Supplementary Table 10 NMR spectroscopic data of sorangicin Y in methanol-d_4 at 500/125 MHz.

#	δ ^{13}C [ppm]	δ ^{1}H [ppm], mult (*J* [Hz])	COSY	HMBC
1	178.8			
2	36.3	2.24, m	3a, 3b	1, 3, 4
3a	26.2	1.62, m	2, 4a	1, 2, 4
3b		1.50, m	2, 4b	1, 2, 4
4a	28.4	1.25, m	3a, 5a, 5b	2, 3, 5, 6
4b		1.18, m	3b, 5a, 5b	2, 3, 5, 6
5a	38.8	1.41, m	4a, 4b, 6	3, 4, 6, 7, 44
5b		1.00, m	4a, 4b, 6	3, 6, 7, 44
6	32.5	2.32, m	5a, 5b, 7, 44	4, 5, 7, 8, 44
7	133.7	5.22, br d (10.2, 1.2, 1.2)	6, 9, 45	5, 6, 8, 9, 44, 45
8	129.5			
9	73.6	4.10, s	7, 10, 45	7, 8, 10, 13, 45
10	66.1	5.42, dd (6.0, 1.1)	9, 11	9, 11, 12, 43
11	124.5	6.09, m	10, 12, 13	9, 10, 12, 13, 14
12	136.9	6.15, dd (9.8, 2.6)	11, 13	10, 11
13	75.0	4.50, m	11, 12, 14a, 14b	9, 11, 14, 15
14a	35.6	2.45, dt (14.1, 10.7, 10.7)	13, 15	12, 13, 15, 16
14b		2.26, m	13, 15, 16	15
15	133.9	5.82, ddd (14.8, 10.0, 4.5)	14a, 14b, 16	13, 14, 16
16	131.5	6.12, m	14b, 15, 17	13, 14, 18
17	135.0	6.28, m	16	15, 16, 18
18	134.0	6.13, m	19	16, 19, 20
19	135.3	6.25, m	18, 20	17, 18, 20, 21
20	131.3	5.68, dd (14.9, 9.0)	19, 21	16, 18, 19, 21, 22
21	73.8	4.51, m	20, 22	19, 20, 22, 23, 46
22	44.7	1.78, ddd (9.9, 6.9, 4.4)	21, 23, 46	20, 21, 23, 24, 46
23	76.8	3.36, m	22, 24a, 24b	22, 24, 25, 27, 46
24a	33.6	1.67, m	23, 24, 25	22, 25, 26
24b		1.48, m	23, 24, 25	22, 23, 25
25	71.2	3.79, m	24a, 24b, 26	23, 26,27, 47
26	37.4	1.50, m	25, 27, 47	25, 47
27	74.6	3.72, m	26, 28	23, 25, 28, 29, 47
28	36.9	2.22, m	27, 29, 30	26, 27, 29, 30

29	131.9	5.37, m	28	28, 30, 31
30	133.0	5.36, m	28, 31	28, 31, 32
31	81.2	3.81, m	30, 32	29, 30, 32, 33, 48
32	42.0	1.38, m	31, 33, 48	30, 31, 33, 34, 48
33	81.1	4.29, d (6.4)	32, 34a, 34b	31, 34, 35, 48
34a	39.6	2.03, m	33, 35	32, 33
34b		1.92, br dd (11.6, 1.3)	33, 35	32, 33, 35
35	77.5	4.37, m	34a, 34b, 36	31, 33, 34, 36
36	81.4	4.58, m	35, 37, 38	37, 38
37	135.4	6.30, m	36, 38	35, 36, 38, 39
38	128.6	7.02, m	36, 37, 39	36, 39, 40
39	138.0	6.46, br t (11.2, 11.2)	38, 40, 42	37, 38, 41
40	127.4	7.26, m	39, 41, 42	36, 38, 41, 42, 43
41	139.3	7.11, m	40, 42	39, 43
42	119.6	5.56, d (11.4)	39, 40, 41	40, 41, 43
43	168.0			
44	22.6	0.76, d (6.6)	6	3, 5, 6, 7
45	14.6	1.56, d (0.6)	7, 9	7, 8, 9
46	9.7	0.86, br d (6.9)	22	22, 23
47	10.7	0.85, br d (7.2)	26	25, 26, 27
48	15.5	0.78, br d (6.7)	32	31, 32, 33

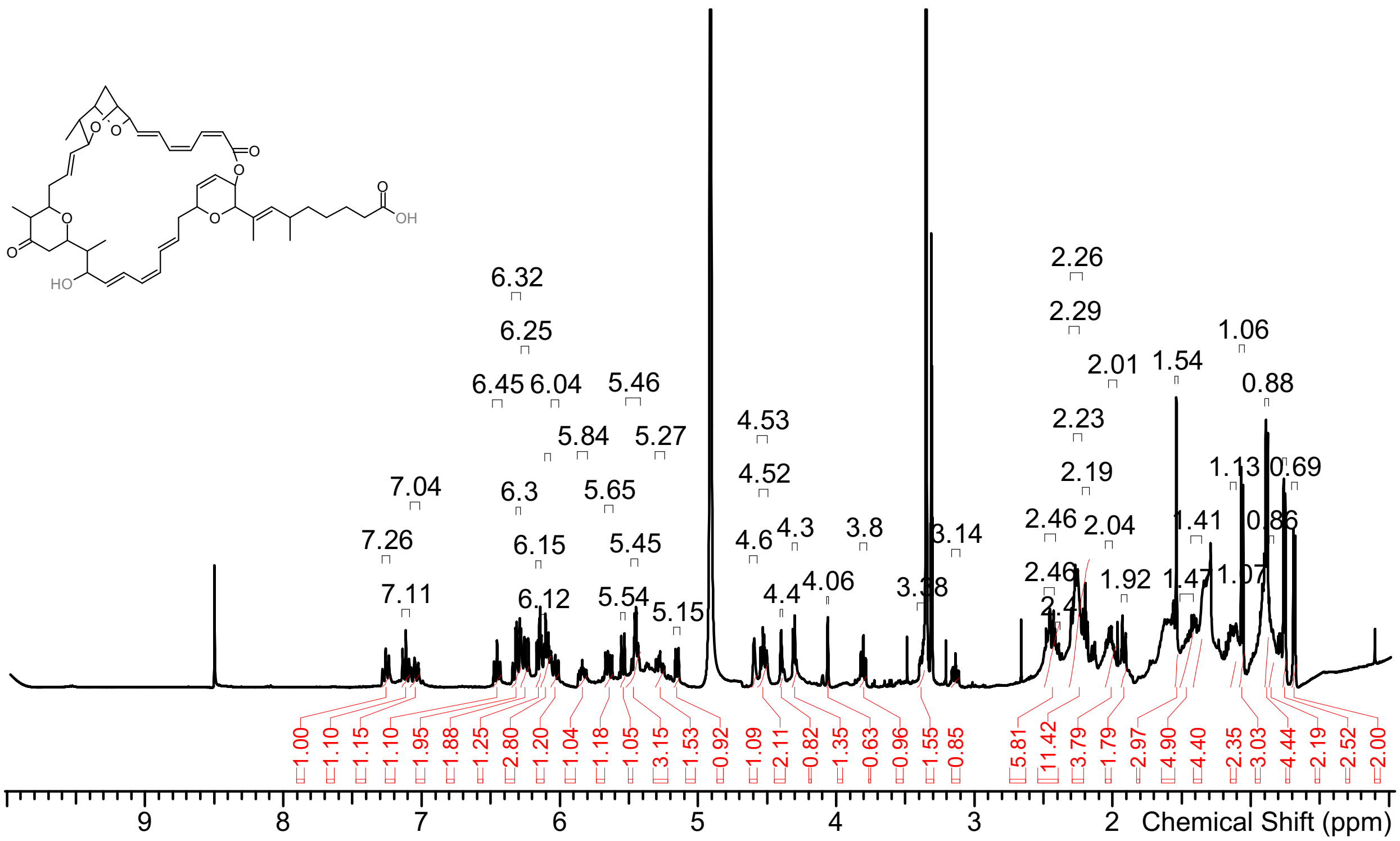

Supplementary Figure 34 ^{1}H-spectrum of sorangicin X in methanol-d_4 at 500 MHz.

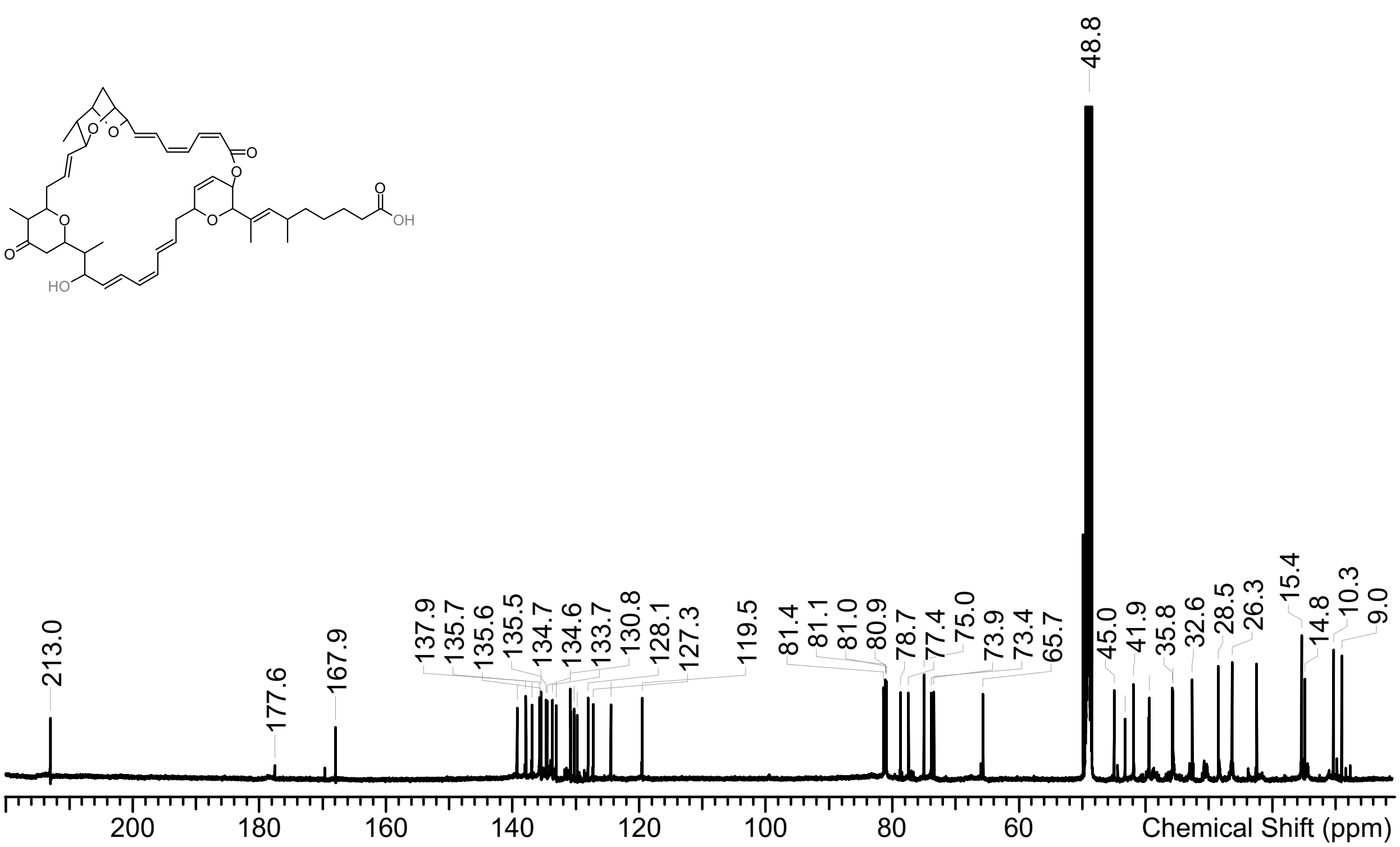

Supplementary Figure 35 ^{13}C-spectrum of sorangicin X in methanol-d_4 at 125 MHz.

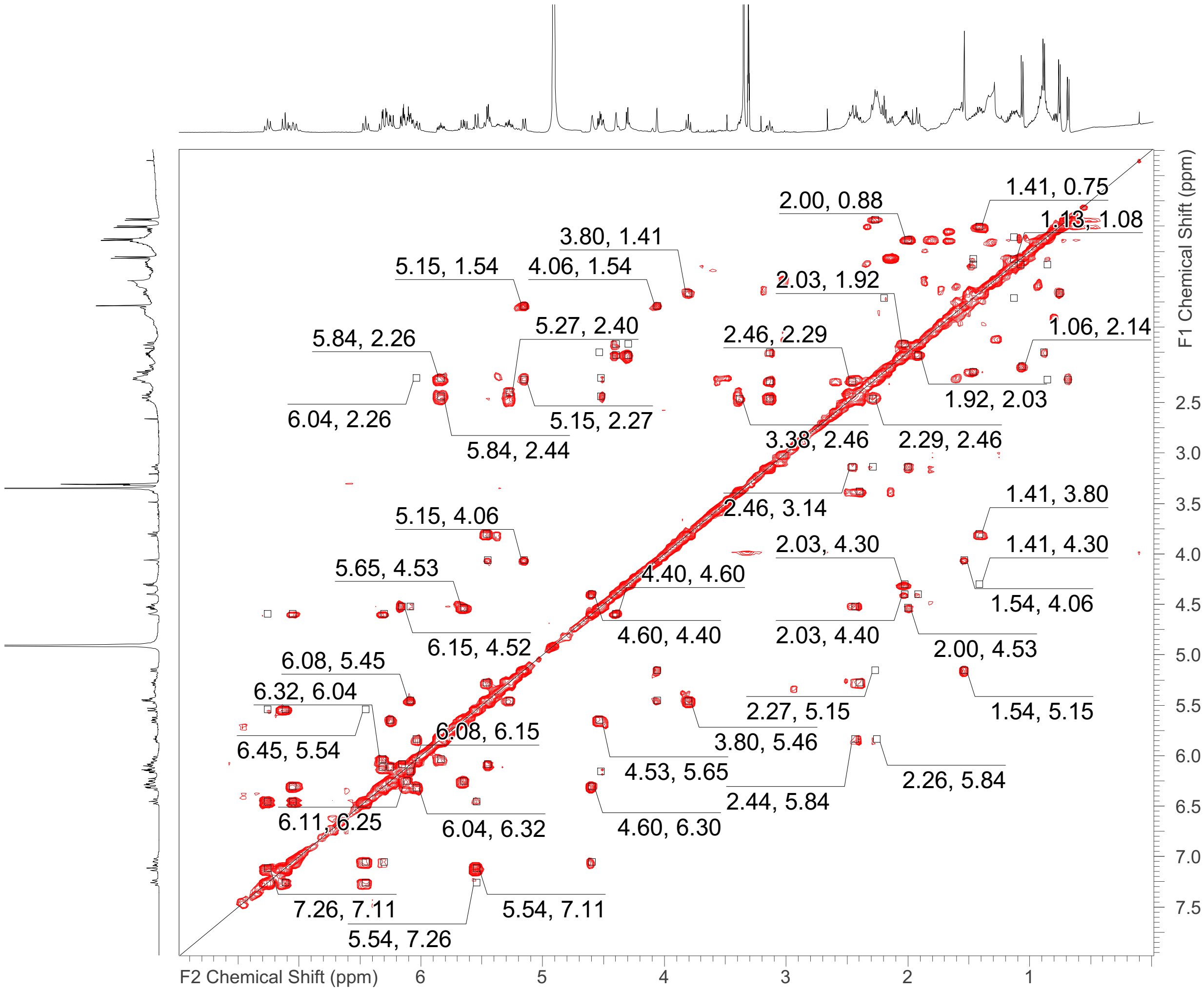

Supplementary Figure 36 COSY spectrum of sorangicin X in methanol-d_4 at 500 MHz.

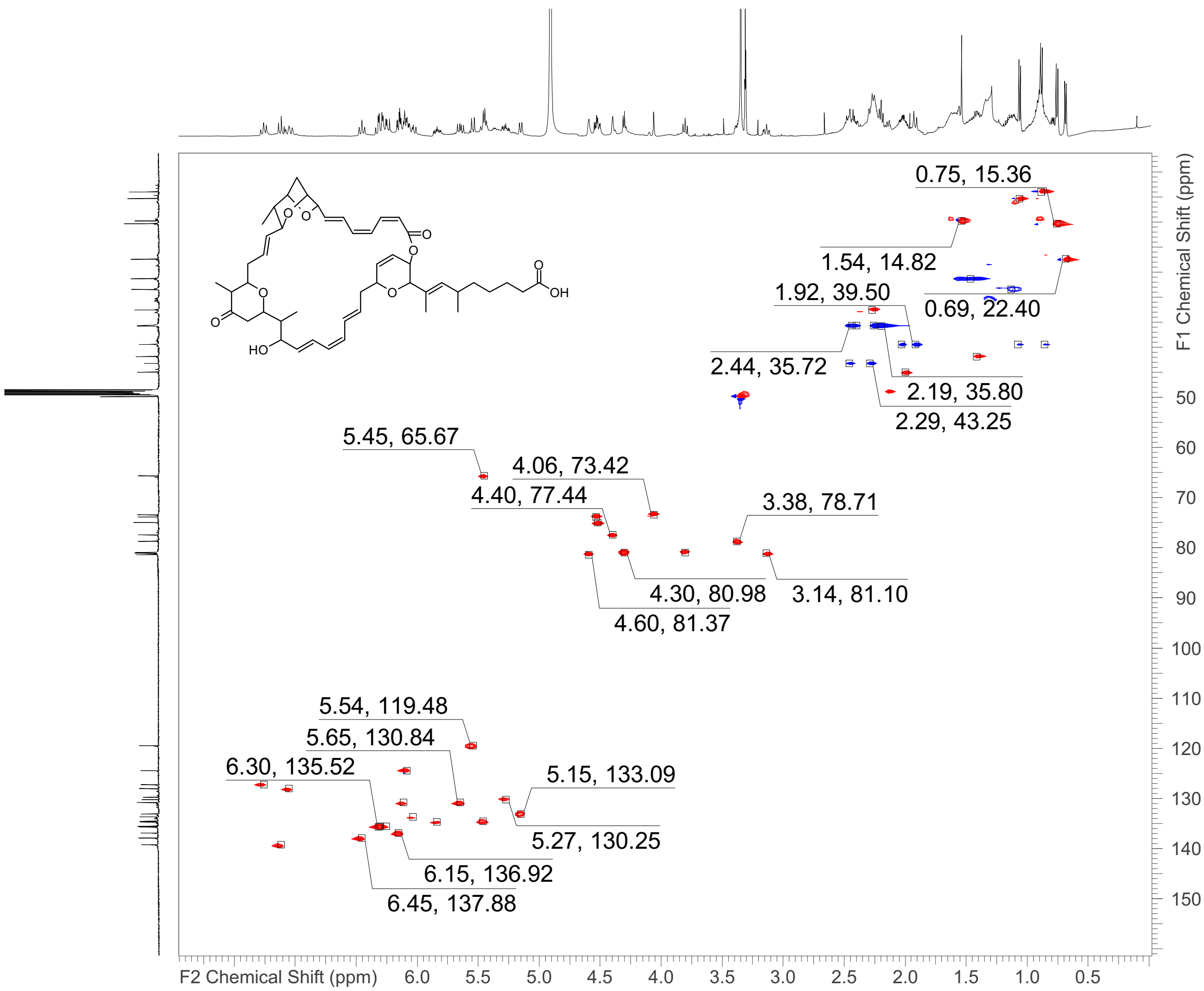

Supplementary Figure 37 HSQC spectrum of sorangicin X in methanol-d_4 at 500/125 MHz.

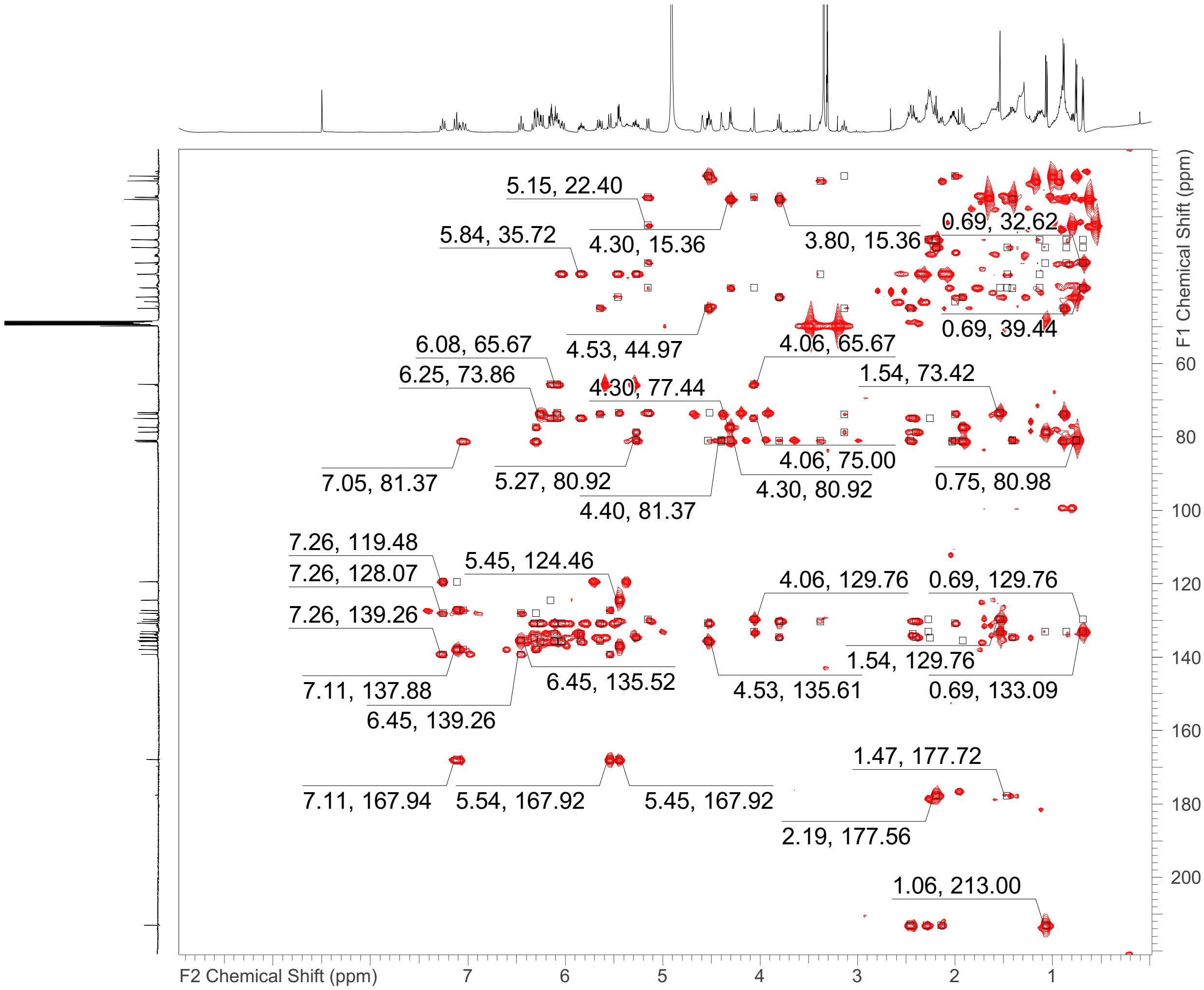

Supplementary Figure 38 HMBC spectrum of sorangicin X in methanol-d_4 at 500/125 MHz.

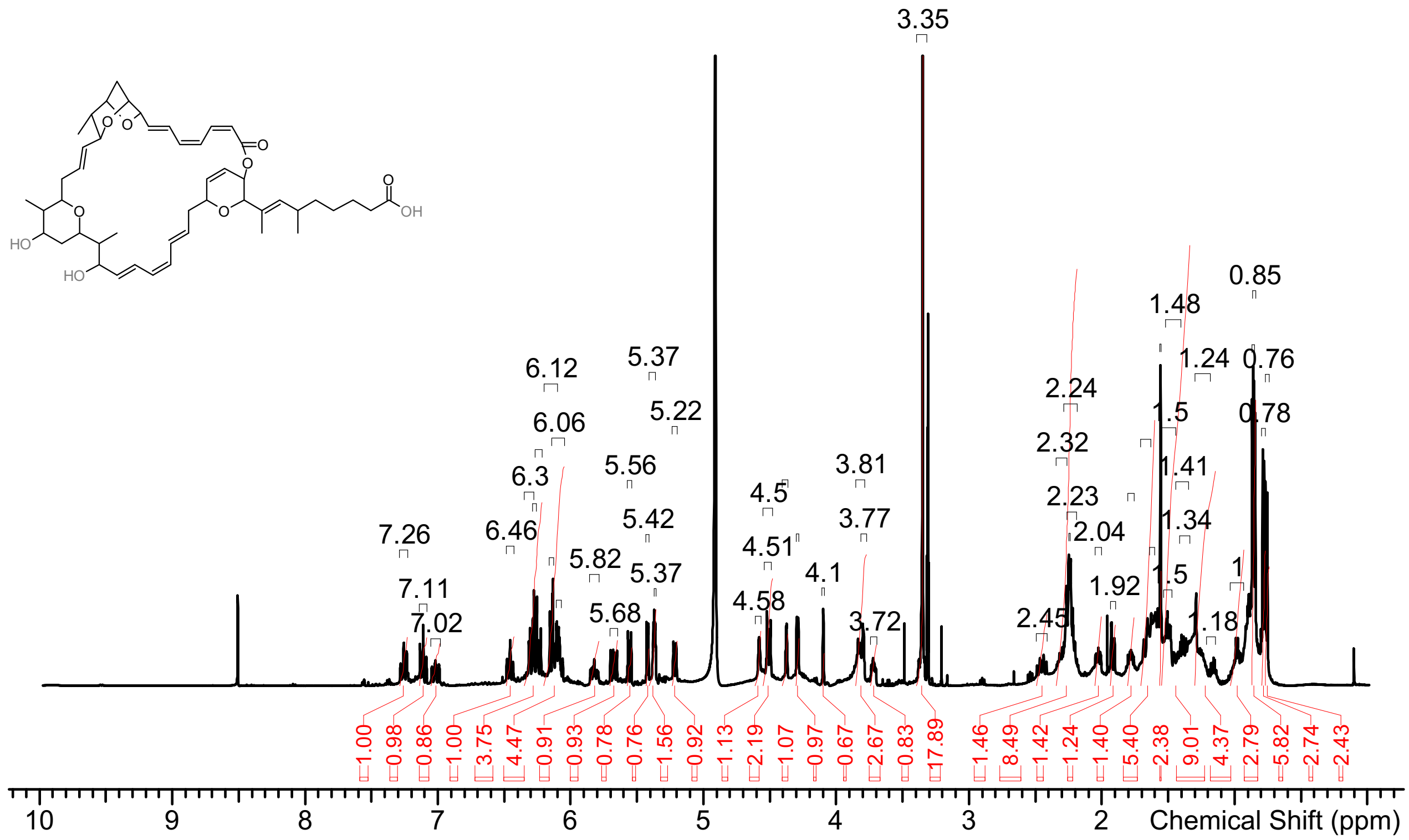

Supplementary Figure 39 ^{1}H-spectrum of sorangicin Y in methanol-d_4 at 500 MHz.

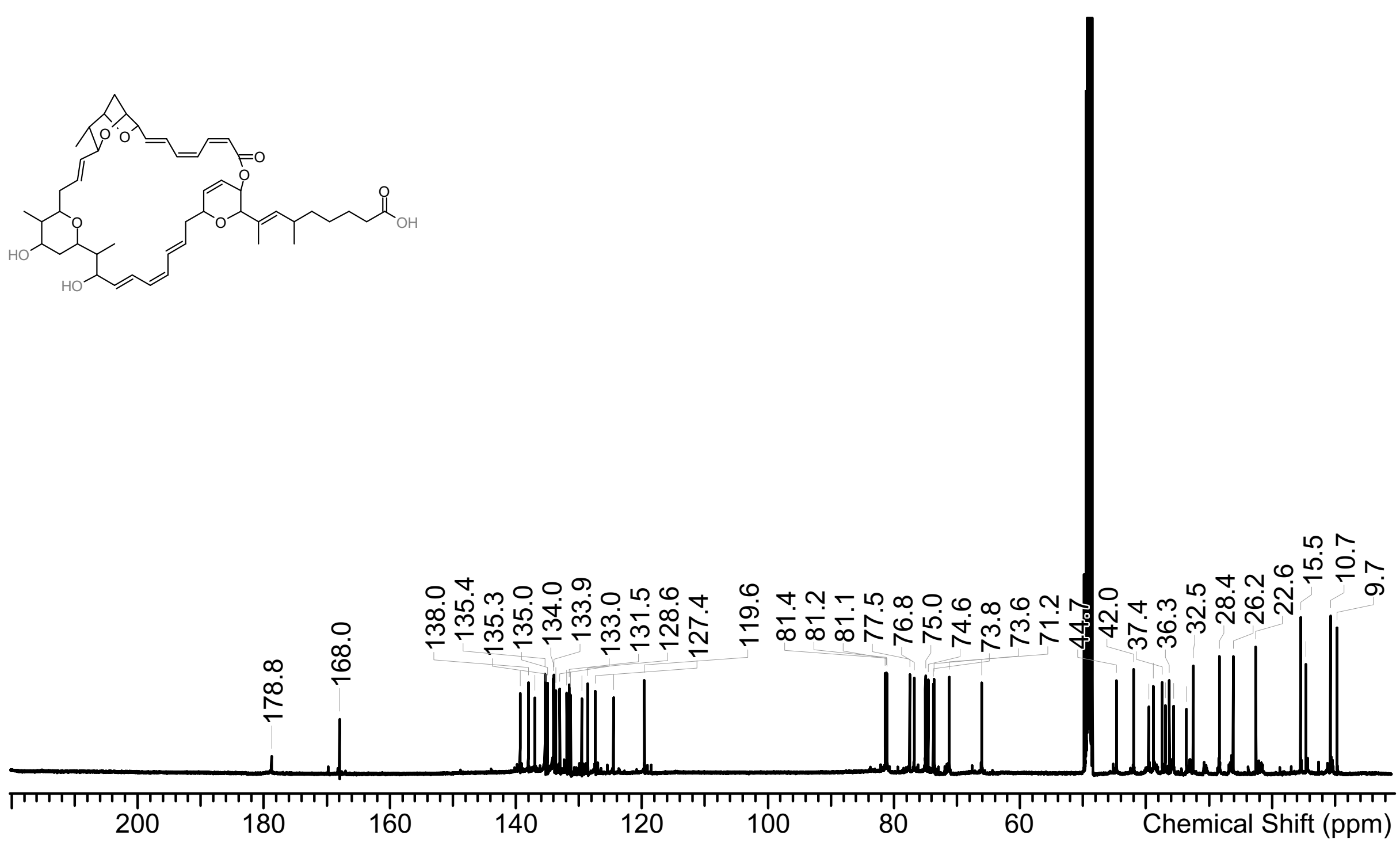

Supplementary Figure 40 ^{13}C-spectrum of sorangicin Y in methanol-d_4 at 125 MHz.

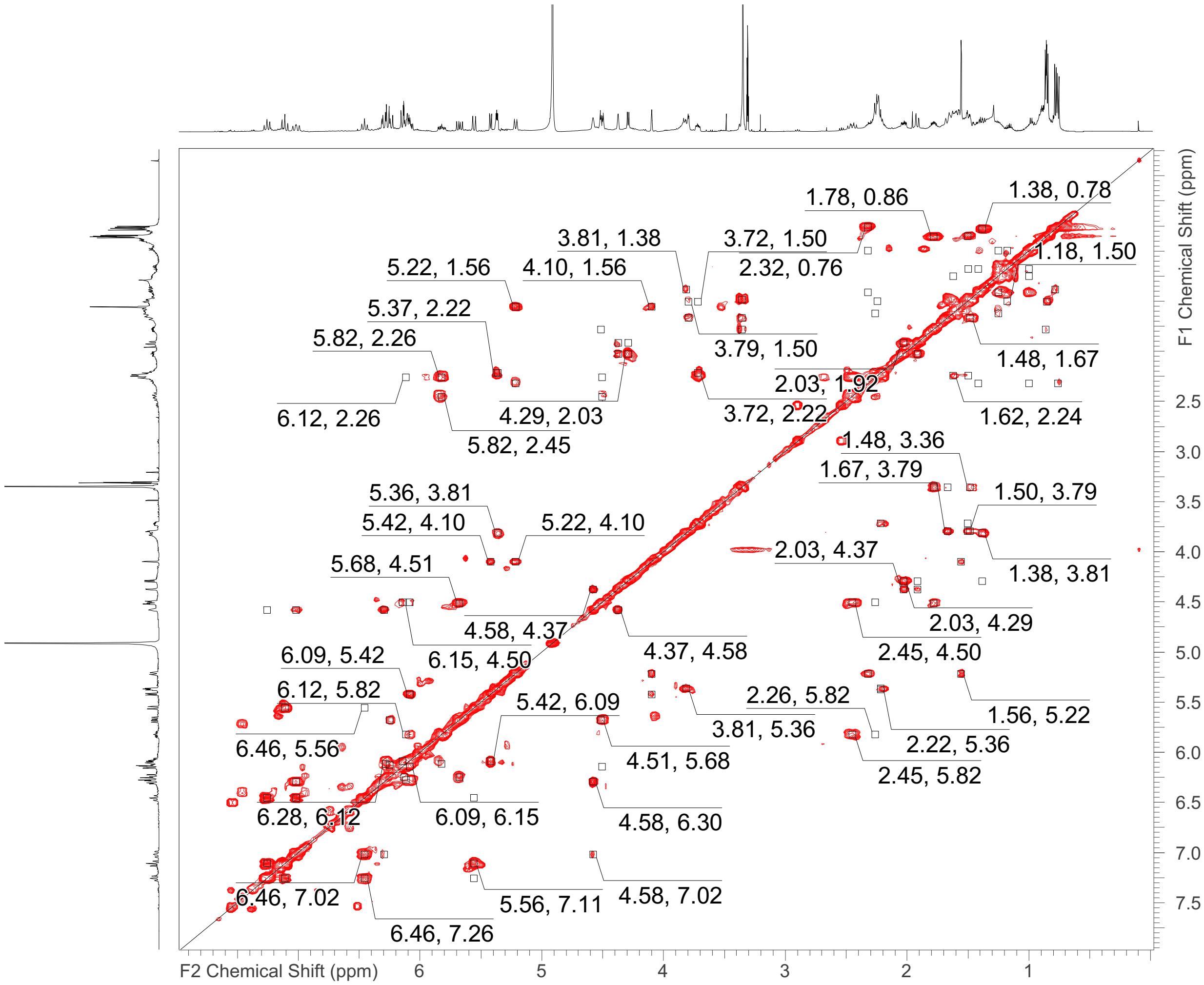

Supplementary Figure 41 COSY spectrum of sorangicin Y in methanol-d_4 at 500 MHz.

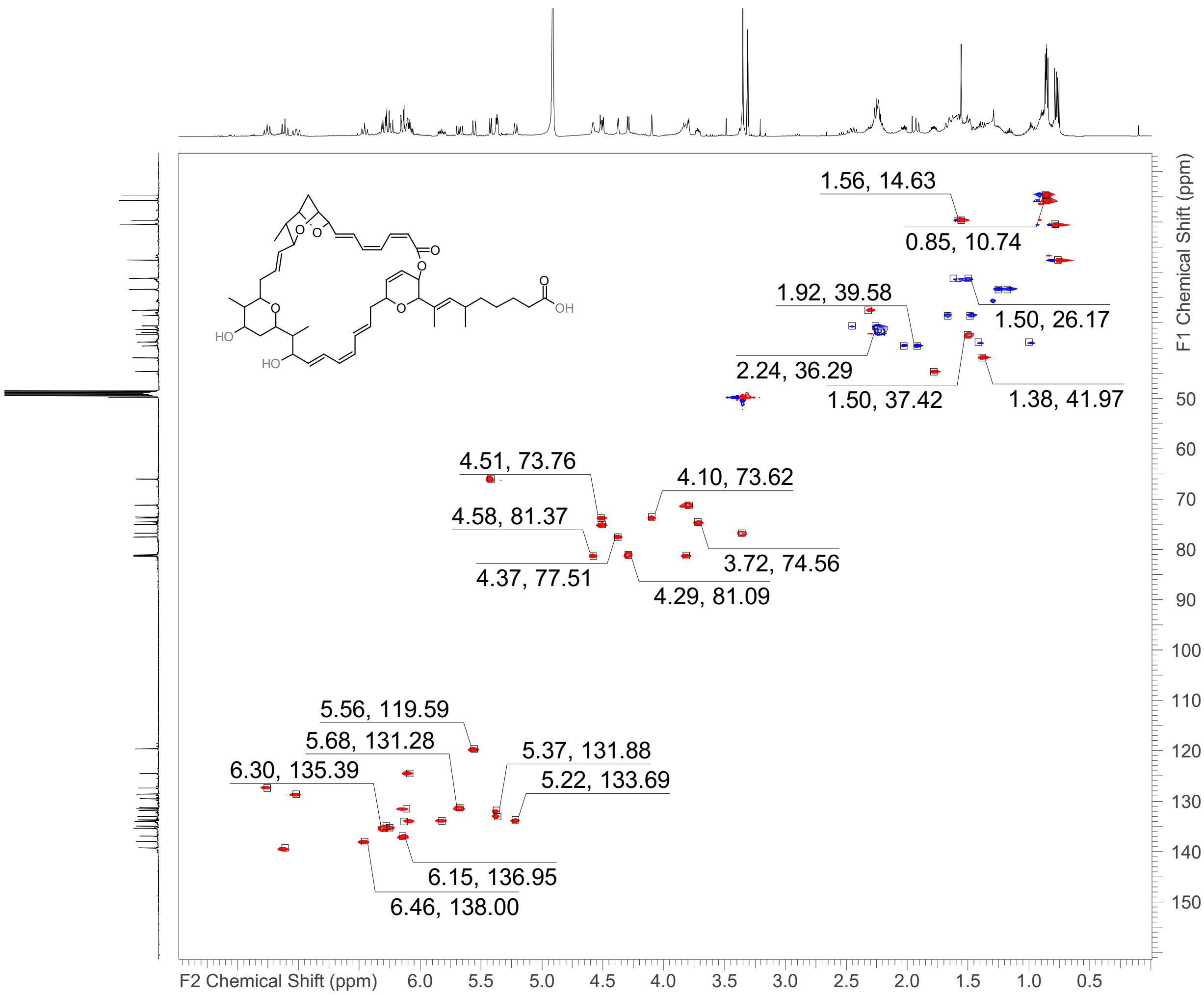

Supplementary Figure 42 HSQC spectrum of sorangicin Y in methanol-d$_4$ at 500/125 MHz.

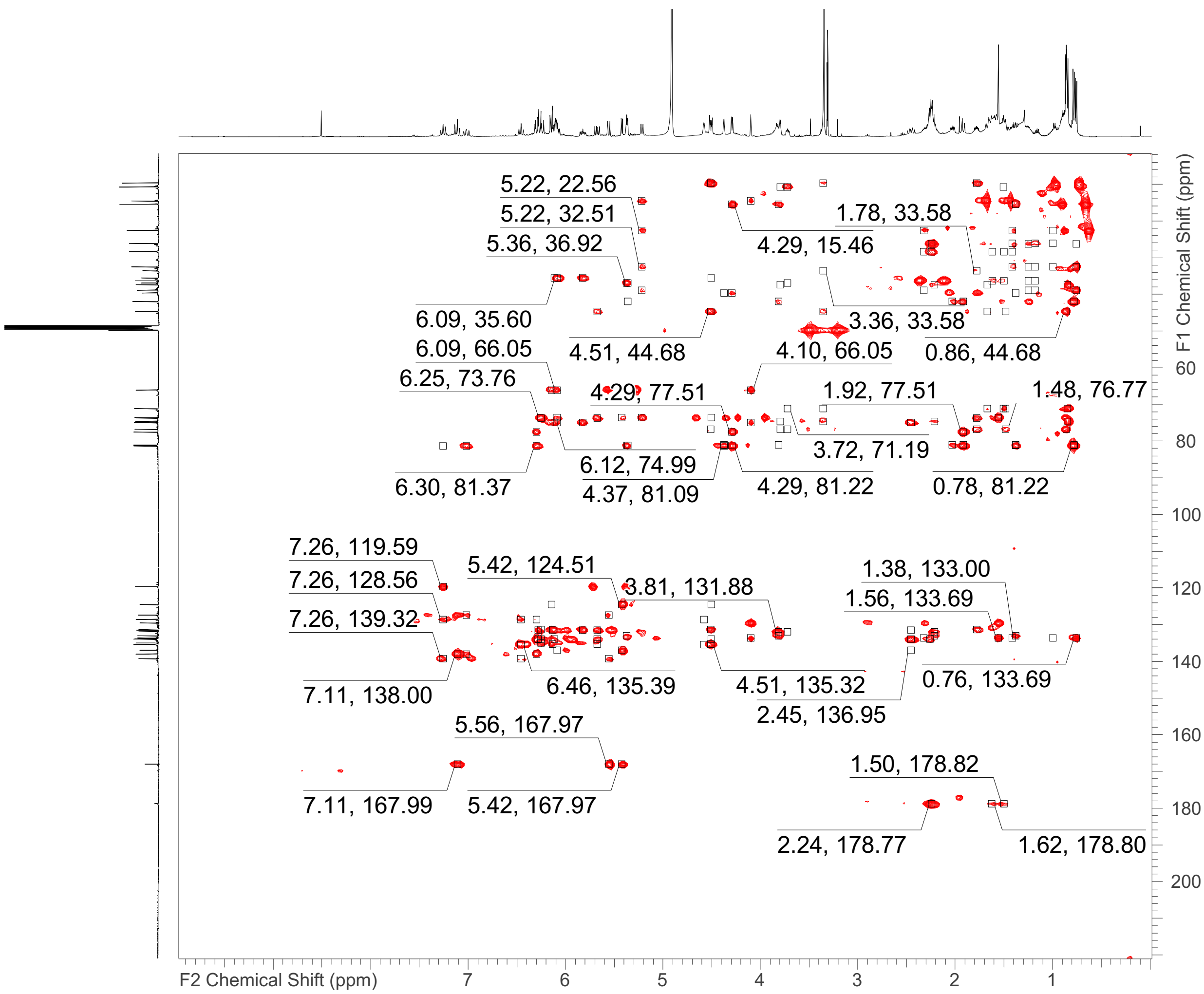

Supplementary Figure 43 HMBC spectrum of sorangicin Y in methanol-d_4 at 500/125 MHz.

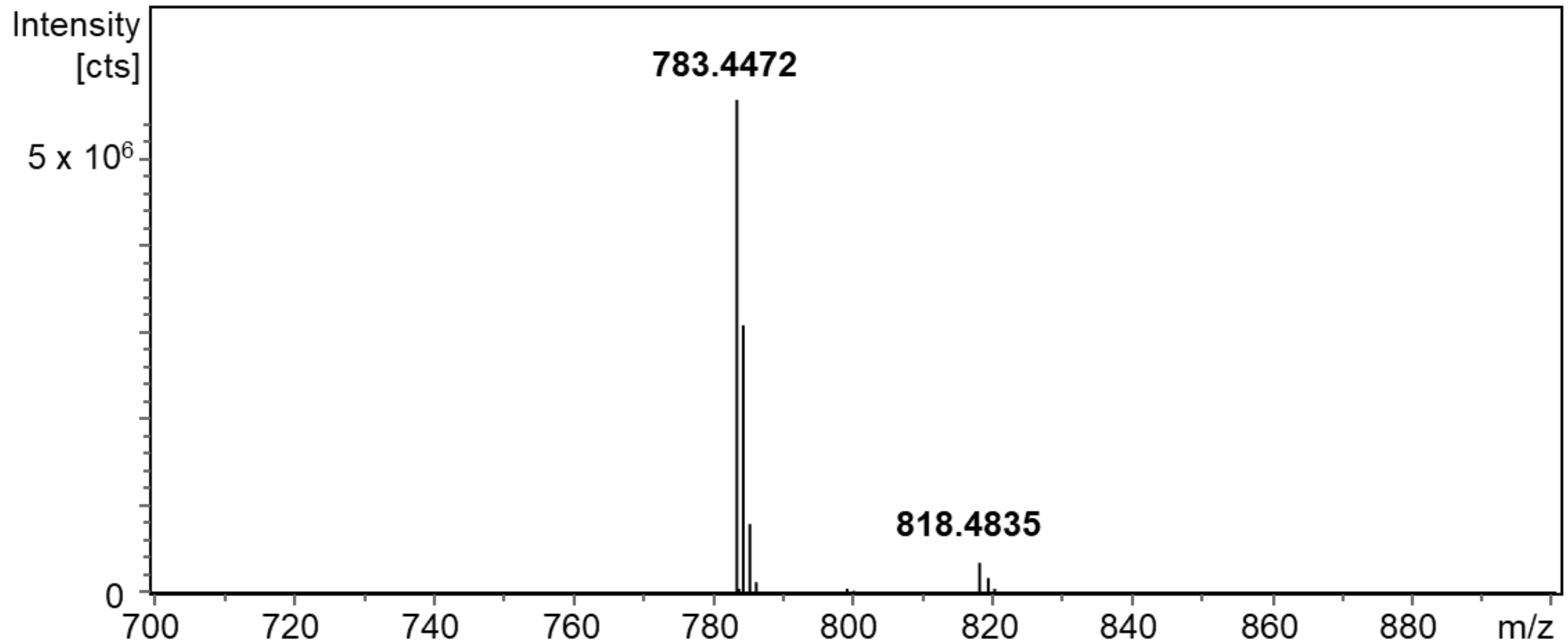

Supplementary Figure 44 MS spectrum of sorangicin X showing $[M-H_2O+H]^+$ at *m/z* 783.4472 and $[M+NH_4]^+$ at *m/z* 818.4835 as most prominent ion types.

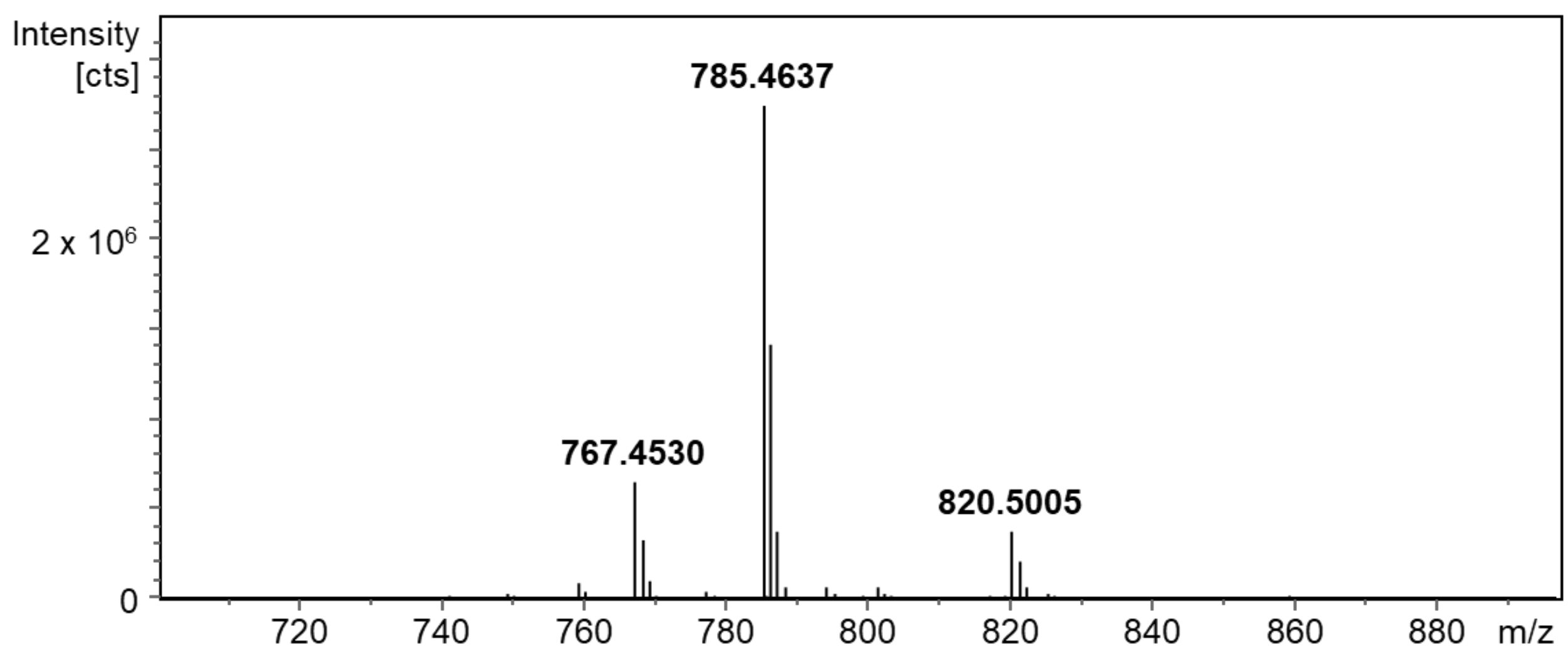

Supplementary Figure 45 MS spectrum of sorangicin Y showing $[M-2H_2O+H]^+$ at 767.4530, $[M-H_2O+H]^+$ at *m/z* 785.4637 and $[M+NH_4]^+$ at *m/z* 820.5005 as most prominent ion types.

Supplementary Note 5 Analysis of the *srx* BGC and comparison to the *sor* BGC.

Comparison of the published *sor* BGC from *S. cellulosum* So ce12[11] with the *sor* BGC from So ce1525 and the new *srx* BGC observed in So ce429 showed a high level of conversation (Supplementary Figure 46A). Differing from the published *sor* BGC from So ce12, the homologs of the large biosynthetic genes *sorG* and *sorH* are condensed into one larger gene in both sorangicin BGCs observed in our studies. This observation was verified by manual analysis of the sequences of all three BGCs. The biggest differences of the *srx* BGC compared to the *sor* BGCs are the absence of a homolog of *sorK* and a much lower identity between *srxC* and *sorC*, compared to all other genes in the BGC.

A comparison of the cluster architecture of the *srx* BGC in So ce429 and the *sor* BGC in So ce1525 (Supplementary Table 11) displayed additional keto-reductase (KR) and dehydratase (DH) domains in modules 11 and 12, both encoded by *srxC*. Moreover, this comparison showed an additional DH domain in module 2, additional acyl carrier protein (ACP) domains in modules 13 and 14 as well as an O-methyl-transferase (oMT) domain in module 18 of the *srx* BGC. However, the additional domains in modules 2 and 18 of the *srx* BGC did not yield in structural differences between sorangicins X and Y compared to sorangicin A. While module 2 of the *sor* BGC needs to accommodate *in trans* acting DH, MT, KR and enoylreductase (ER) domains to yield the final configuration of the incorporated ketide[10], module 2 of the *srx* BGC already features the DH domain, but still needs the other domains to act *in trans* to explain its final product. The additional oMT domain in module 18 on the other hand is in accordance with the predicted substrate specificity of the ketosynthase (KS) domains in module 19 of the *srx* and *sor* BGC, as they are predicted to incorporate a β-methoxy-residue, according to a phylogenetic analysis[11,12]. In the matured natural product carbon C-33, which might have been methoxylated in module 18, is part of the unusual bicyclic ether moiety that is shared between sorangicin X, Y, A, and P. As the biochemical reactions that lead to this dioxabicyclooctane moiety remain elusive[10,11], the original hydroxyl- or methoxy-residue at C-33 is likely lost during the maturation of the sorangicin scaffold.

The additional KR and DH domains in modules 11 and 12 of the *srx* BGC are of greater interest, as the structural differences between sorangicin X and Y compared to sorangicin A are incorporated between modules 10 and 14 of the respective biosynthetic machineries (Supplementary Figure 46B). The additional double bond in sorangicin X and Y is incorporated by module 10 of the biosynthetic machinery and fits the observed set of reducing domains (KR and DH) of that module in the *srx* and *sor* BGCs. However, the KS domains in module 11 of both pathways share the predicted substrate specificity of a fully reduced ketide[11]. Consequently, matching the predicted substrate specificity of this KS domain as well as the observed double bond in the final natural product would entail a full reduction of the growing natural product in module 10 by an *in trans* acting enoyl reductase (ER) domain and a subsequent oxidation at a later step of the biosynthesis. Therefore, it is likely that, in contrast to the prediction, the KS domain in module 11 of the *srx* BGC accepts substrates with a double bond at the α, β position. Module 11 of the *srx* BGC differs from the respective module in the *sor* BGC as it features a KR domain, yielding the observed double bond in sorangicins X and Y. The corresponding double bond in sorangicin A can be only explained by an *in trans* acting KR domain in that module.

When describing the biosynthesis of sorangicin A, the authors discussed the discrepancy between the biosynthetically predicted incorporation of an α-methyl, β-hydroxyl moiety by module 12 and the observed α-hydroxyl, β-hydroxyl moiety that was incorporated instead. They hypothesized that in module 12 the first acyl transferase (AT_a) domain of the *sor* BGC incorporates hydroxymalonate instead of malonate and that the hydroxyl functionality at the α position precludes a subsequent C-methylation reaction[11]. Interestingly, sorangicin X and Y feature the α-methyl, β-hydroxyl moiety that was predicted to be incorporated by module 12 of the *sor* BGC. While both AT domains of the *srx* BGC share an amino acid similarity of 91.3% and 91.0% as well as substrate specific fingerprints with their respective counterparts, module 12 of the *srx* BGC contains a DH domain that is absent in module 12 of the *sor* BGC. As this additional DH domain in module 12 of the *srx*

BGC is the only obvious difference to the *sor* BGC, it seems possible that it might enable the methylation of the α-carbon. However, this possibility needs further experimental proof as, to the best of our knowledge, there are no reports about non-canonical DH domains that act on α-hydroxyl residues or on vicinal alcohols.
The additional tandem ACP domains in modules 13 and 14 of the *srz* BGC do not affect the structure of the molecule, such tandem ACP domains are believed to increase the efficiency of rate-limiting biosynthetic steps[13]. Therefore, the most likely explanation for the structural differences between sorangicin X and Y is an incomplete reduction of the growing polyketide by the KR domain in module 14. Next to the architecture of the *srx* BGC, the differences in their production titers point towards sorangicin Y as main product and sorangicin X as byproduct of this biosynthetic pathway (Supplementary Figure 46C).

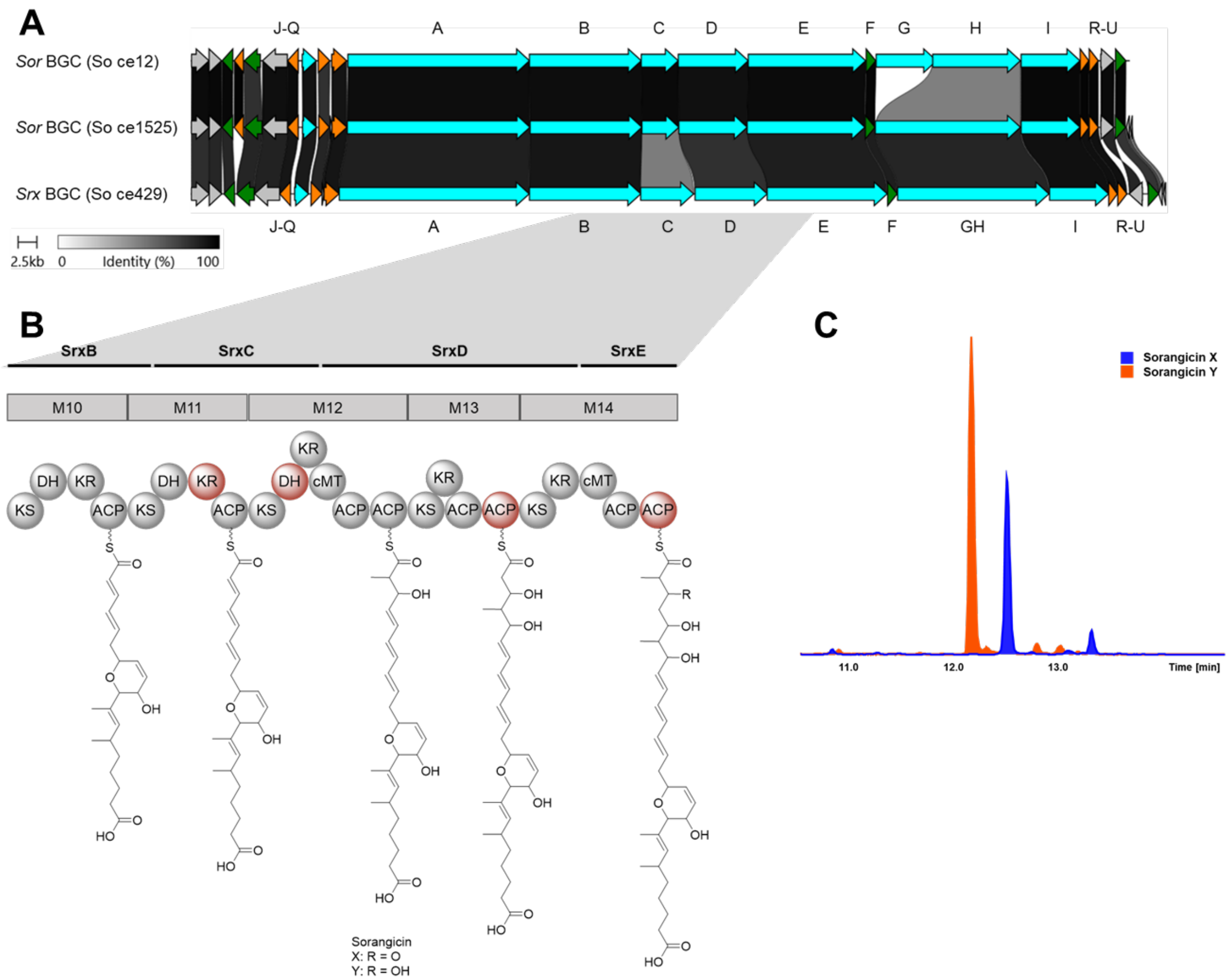

Supplementary Figure 46 The srx BGC and the biosynthesis of sorangicins X and Y compared to the sor BGC and the biosynthesis of sorangicin A. A The published sor BGC from S. cellulosum So ce12 compared to the sor BGC from S. cellulosum So ce1525 and the srx BGC from S. cellulosum So ce429. Core biosynthetic genes are displayed in blue, additional biosynthetic genes in orange, resistance genes in green, other genes are colored in grey. B Incorporation of differences in the chemical structures of sorangicin X and Y compared to sorangicin A by the modules 10 to 14 in the srx BGC. Additional domains compared to the sor BGC are marked in red. C Relative production of sorangicin X (blue) and Y (red) in CyH medium.

Supplementary Table 11 Comparison of the cluster architecture of the *srx* BGC in *S. cellulosum* So ce429 and the *sor* BGC in *S. cellulosum* So ce1525. Difference compared to the published *sor* BGC in *S. cellulosum* So ce12[11] are marked bold.

***srx* BGC So ce429**			***sor* BGC So ce1525**		
SrxA	1	KS	KS	1	SorA
		DH	DH		
		KR	KR		
		ACP	ACP		
	2	KS	KS	2	
		**DH**			
		ACP	ACP		
		ACP	ACP		
	3	KS	KS	3	
		DH	DH		
		KR	KR		
		cMT	cMT		
		ACP	ACP		
	4	KS	KS	4	
		KR	KR		
		ACP	ACP		
	5	KS	KS	5	
		ACP	ACP		
	6	KS	KS	6	
		KR	KR		
		ACP	ACP		
	7	KS*	KS*	7	
SrxB		DH	DH		SorB
		ACP	ACP		
		DH	DH		
	8	KS*	KS*	8	
		PS	PS		
		ACP	ACP		
	9	KS	KS	9	
		DH	DH		
		ACP	ACP		
	10	KS	KS	10	
		DH	DH		
		KR	KR		
		ACP	ACP		
	11	KS	KS	11	
SrxC		DH	DH		SorC
		**KR**			
		ACP	ACP		
	12	KS	KS	12	

		**DH**			
		KR	KR		
		cMT	cMT		
		ACP	**-**		
		ACP	ACP		
		KS	KS		
SrxD	13	KR	KR	13	SorD
		ACP	ACP		
		**ACP**			
		KS	KS		
		KR	KR		
	14	cMT	cMT	14	
		ACP	ACP		
		**ACP**			
		KS	KS		
		DH	DH		
	15	PS	PS	15	
		KR	KR		
SrxE		ACP	ACP		SorE
		KS	KS		
	16	DH	DH	16	
		KR	KR		
		ACP	ACP		
		KS	KS		
		DH	DH		
	17	KR	KR	17	
		cMT	cMT		
		ACP	ACP		
		KS	KS		
	18	**oMT**		18	
		ACP	ACP		
		KS	KS		
		DH	DH		
	19	PS	PS	19	
		KR	KR		
SrxGH		ACP	ACP		SorGH
		KS	KS		
	20	DH	DH	20	
		KR	KR		
		ACP	ACP		
		KS	KS		
	21	KR	KR	21	
		ACP	ACP		
	22	KS*	KS*	22	

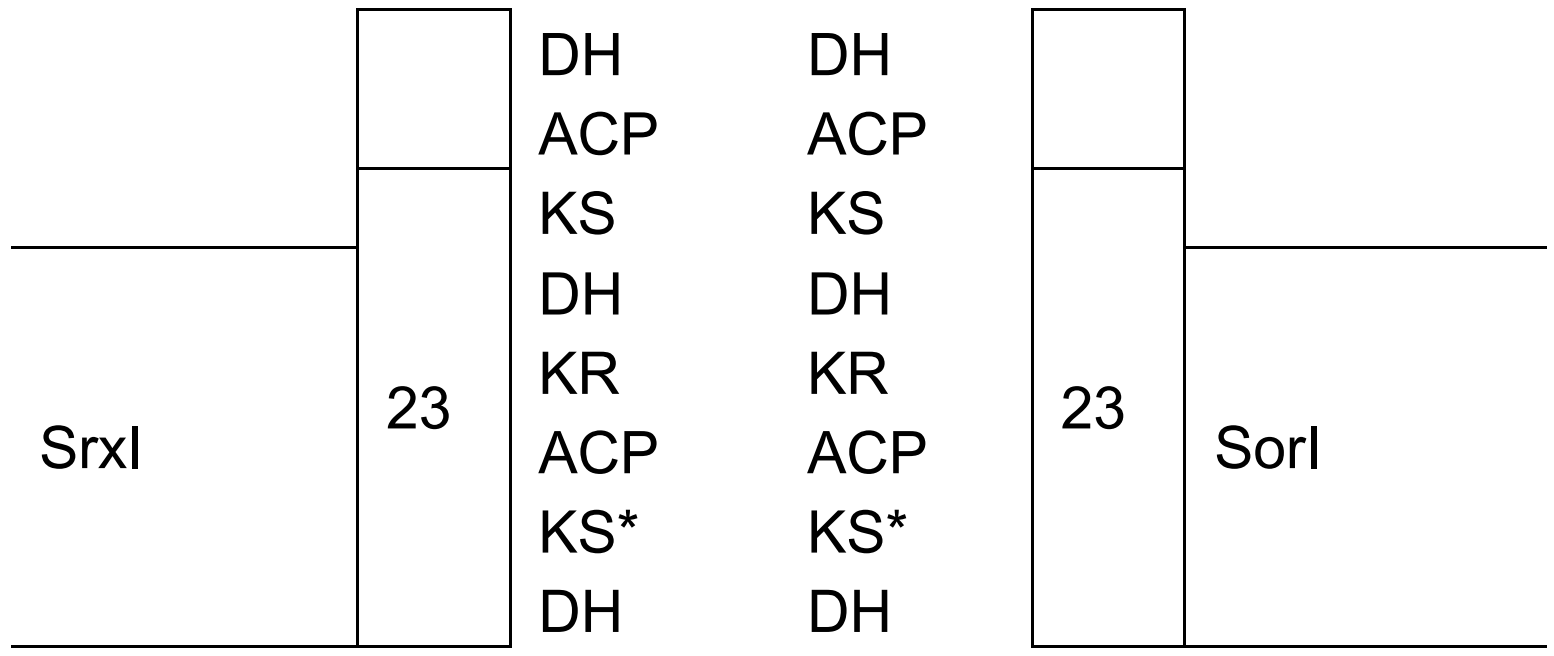
SrxI
23
DH
ACP
KS
DH
KR
ACP
KS*
DH
DH
ACP
KS
DH
KR
ACP
KS*
DH
23
SorI

Supplementary Table 12 Biological activities of sorangicin X and Y.

Test organism	Sorangicin A	Sorangicin X	Sorangicin Y	Rifampicin (control)
E. faecium DSM-20477	4	4	8	>0.64
S. aureus Newman	0.2-0.32	0.16-2	0.2-0.32	0.006
S. aureus Newman RIF^R	>64	>64	>64	>64
S. *aureus* N315 (MRSA)	0.2-0.32	0.05-0.08	0.16-0.2	≤0.003
A. baumannii DSM-30008	32	8	16-32	4
A. baumannii DSM-30008 RIF^R	64	16	64	>64
E. coli BW25113 (WT)	>64	>64	>64	32
E. coli Δ*acrB*	64	64	>64	8
E. coli Δ*tolC*	64	>64	>64	16
P. aeruginosa PA14	>64	>64	>64	>64

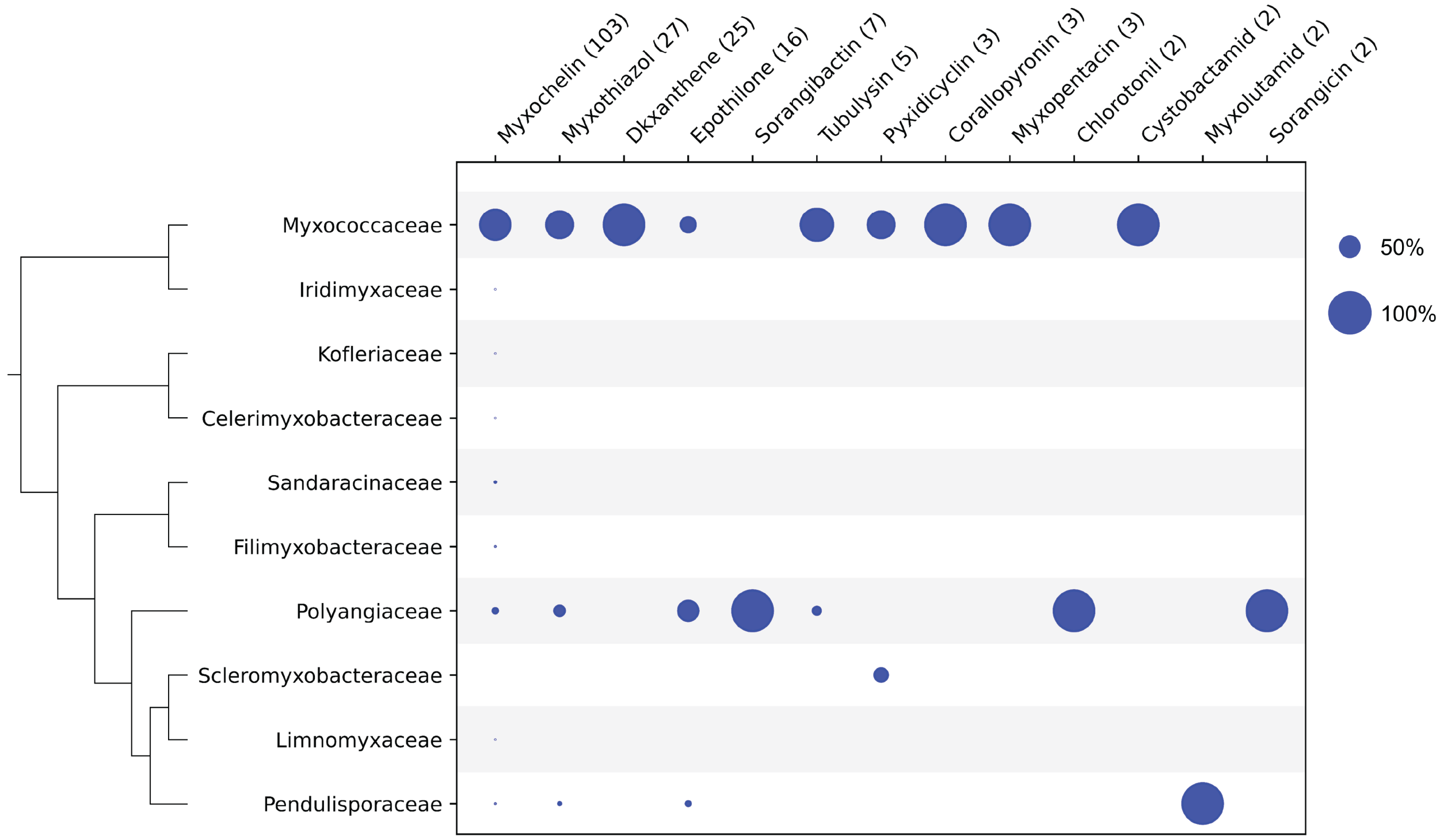

Supplementary Figure 47 Distribution of characterized *Myxococcota* biosynthetic gene clusters (BGCs) across taxonomic groups. Dot size indicates the relative abundance of each BGC, with numerical values denoting absolute occurrences in previously reported *Myxococcota* genomes and in those presented in this study. The accompanying phylogenetic tree is resolved at the family level.

Supplementary Note 6 Analysis of the expanded NCBI-derived *Myxococcota* dataset.

We retrieved 6,632 publicly available *Myxococcota* sequence entries from NCBI (accessed on April 13, 2026). To reduce redundancy, the dataset was dereplicated using the galah tool[13], yielding 4,206 non-redundant entries that were used for all subsequent analyses. This NCBI-derived dataset is not directly comparable to isolate genomes, as they are often highly fragmented (Supplementary Figure 48) and ~96% represent metagenome-assembled genomes (Supplementary Figure 49). Using antiSMASH[4], these non-redundant entries yielded 37,473 BGCs, of which ~79% (29,486) were fragmented. Notably, the distribution of biosynthetic classes in this expanded dataset deviates from that observed in both high-quality public and newly sequenced *Myxococcota* genomes (Supplementary Figure 50). The most striking difference is the marked reduction in the NRPS-PKS hybrid clusters. In high-quality public and newly sequenced *Myxococcota* genomes, these clusters account for 19.2% and 13.5% of total BGCs, respectively. In contrast, their proportion drops sharply to 7.6% in unfragmented and just 3.4% in fragmented NCBI sequence entries. Conversely, the relative abundance of standalone PKS and NRPS clusters increases substantially in the fragmented NCBI dataset. This inverse trend strongly suggests that fragmentation causes prediction software to misclassify large, multi-domain NRPS-PKS hybrid clusters as separate PKS and NRPS clusters. To further assess whether this extended dataset harbors additional non-redundant biosynthetic diversity, we performed a complementary GCF analysis using the unfragmented BGCs recovered from NCBI *Myxococcota* sequence entries. This filtering step was necessary to avoid artificial inflation of GCF counts caused by fragmented clusters. In total, 7,987 non-fragmented BGCs were clustered into 5,612 GCFs, of which only 1,435 (~25.5%) overlapped with GCFs identified in high-quality public and newly sequenced *Myxococcota* genomes as well as MIBiG (Supplementary Figure 51). The remaining majority of GCFs were new, hinting at a large number of potential new secondary metabolites encoded in these data. The additional non-redundant NCBI sequence entries were not further included in the rarefaction analysis, as this approach requires well-defined genome counts that cannot be reliably inferred from these data. In particular, the high degree of BGC fragmentation would lead to artificial inflation of GCF estimates, whereas restricting the analysis to only unfragmented BGCs would introduce bias in genome representation, given that fragmented and complete clusters often originate from the same entries. Nevertheless, the large number of additional GCFs identified in these entries further underscores the substantial, yet underexplored, biosynthetic potential within the *Myxococcota* phylum.

To complement the genome-based taxonomic analyses, 16S rRNA genes were identified from the dereplicated sequence entries using barrnap (v1.10.5)[14]. Predicted 16S rRNA gene sequences shorter than 1,200 bp were discarded using seqkit (v2.9.0)[15] to retain only near full-length or sufficiently informative fragments. For genomes in which multiple 16S rRNA gene copies were detected, only the longest 16S rRNA gene sequence was retained for downstream analysis. The resulting 16S rRNA gene sequences were compared against the 16S rRNA gene sequences extracted from the 154 genomes sequenced in this study, together with representative type-strain genomes, using BLASTn (v2.13.0+)[16]. BLASTn hits were filtered based on sequence identity and query coverage. Specifically, hits were retained when they showed a minimum query coverage of 80 % and met the corresponding identity threshold: ≥96.3 % sequence identity for genus-level assignment and ≥98.6 % sequence identity for species-level assignment. Pairwise average nucleotide identity (ANI) values were calculated using fastANI (v1.34)[17] between the dereplicated

sequence entries and the 154 genomes in this study, together with the representative type-strain genomes. For downstream interpretation, ANI values of ≥95 % were considered indicative of species-level genomic relatedness. Taxonomically, using 16S RNA and ANI analysis, we could assign 114 of these sequence entries to species represented by our new strains and 150 to our new genera (Supplementary Table 13).

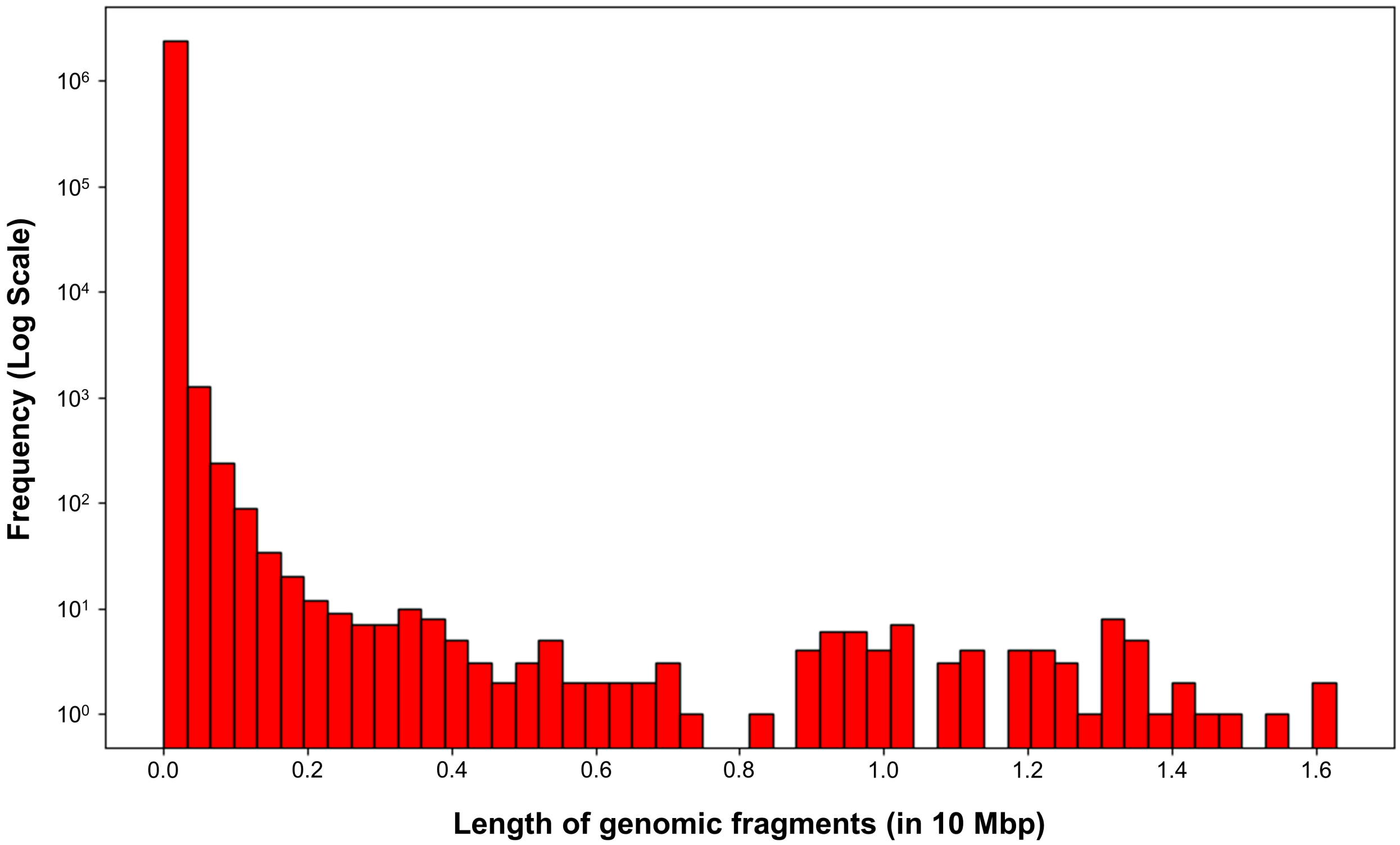

Supplementary Figure 48 Histogram showing the size distribution of genomic fragments in NCBI sequencing entries. A total of 2,414,453 fragments were identified across 4,206 non-redundant entries. The logarithmic y-axis reveals a highly skewed distribution, where the vast majority of the dataset consists of extremely short, fragmented sequences, with a long tail of rare, larger contiguous fragments.

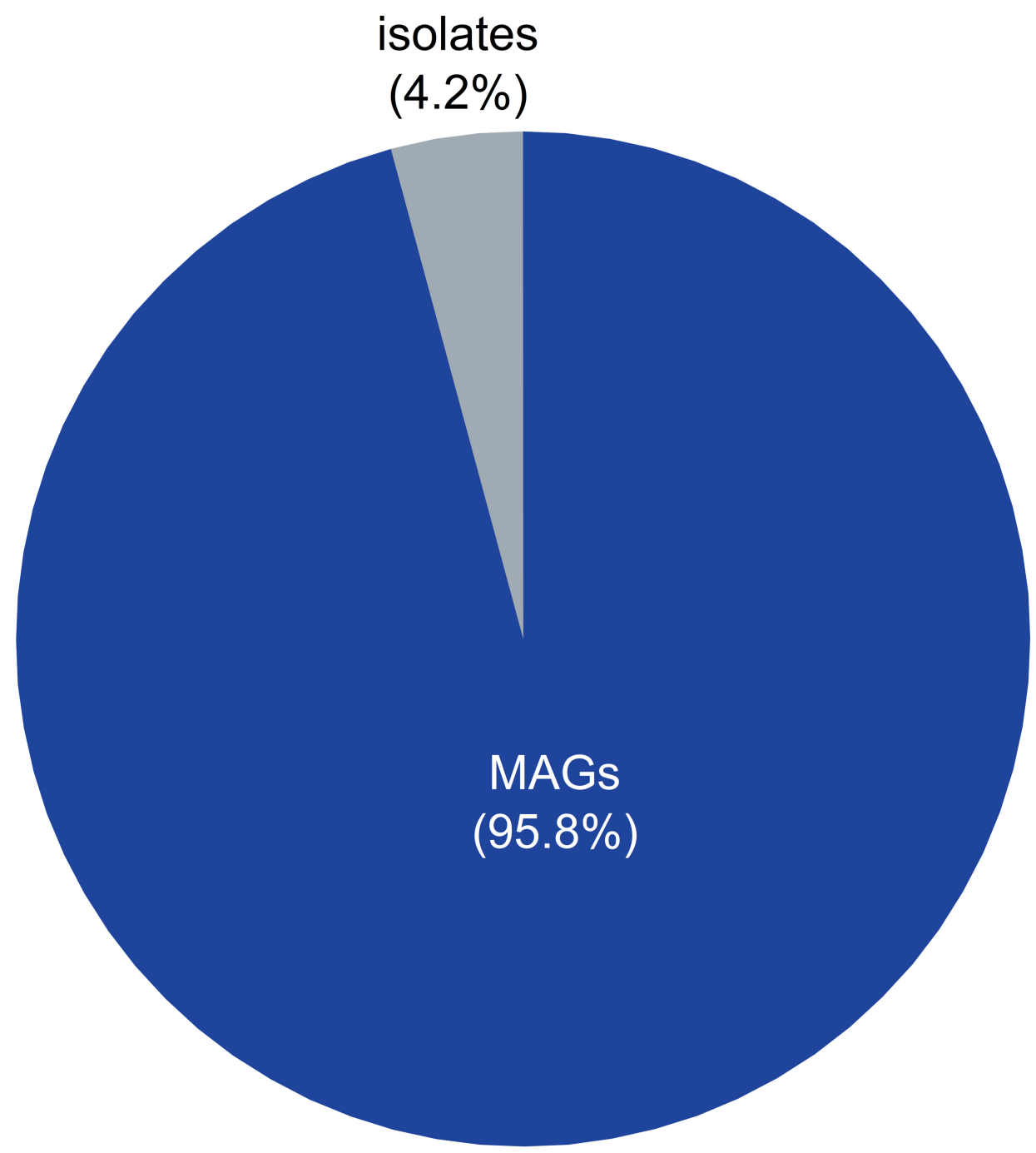

Supplementary Figure 49 Composition of MAGs and isolates among the 4,206 non-redundant NCBI sequencing entries from *Myxococcota* phylum

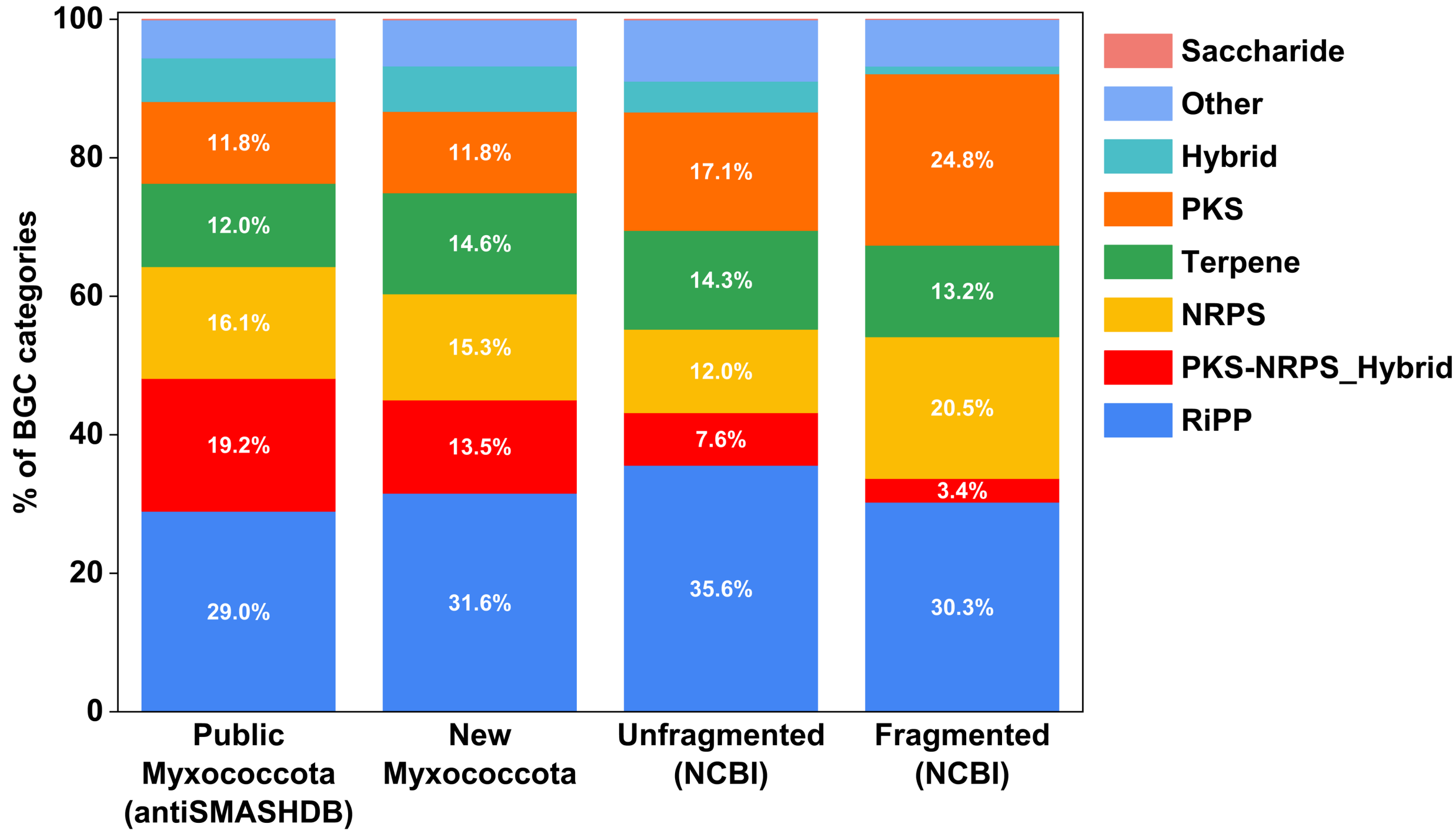

Supplementary Figure 50 Distribution of BGCs into major biosynthetic classes across four *Myxococcota* datasets, including unfragmented and fragmented BGCs from NCBI sequencing entries.

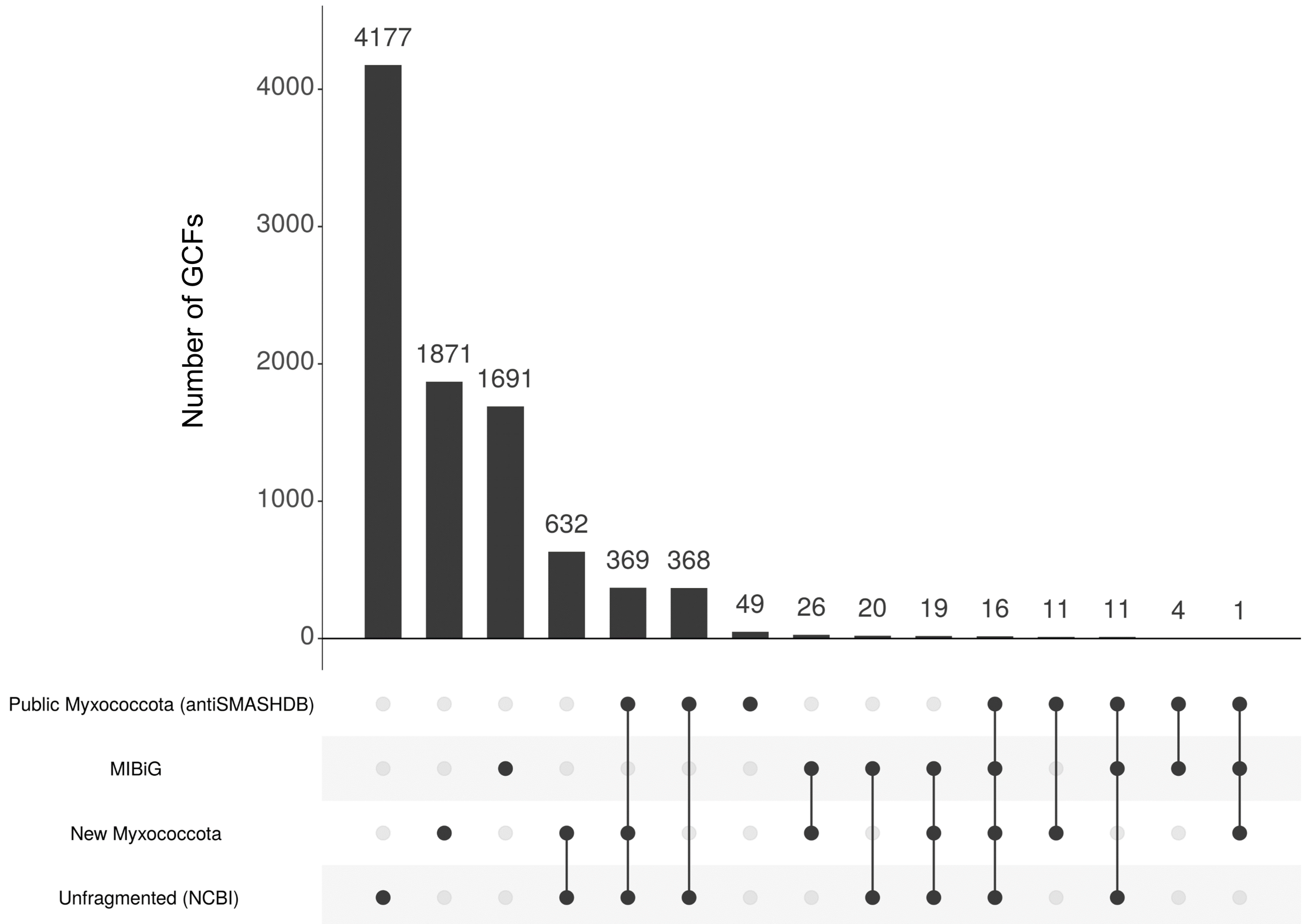

Supplementary Figure 51 Upset plot showing the number of unique and shared GCFs between different *Myxococcota* datasets, including unfragmented BGCs from NCBI sequencing entries.

Supplementary Note 7 Selecting the clustering thresholds for BiG-SCAPE[18] and BiG-SLiCE[19].

We evaluated the effect of different BiG-SCAPE similarity thresholds on clustering quality by comparing the resulting GCFs against compound-level similarity. First, we downloaded all BGCs from MIBiG (v3.0)[20] together with the SMILES strings of their known products. Using RDKit[21], we computed Extended Connectivity Fingerprints for all compounds and calculated pairwise Tanimoto similarities to generate a compound similarity matrix. K-means clustering was then applied to group compounds, with the optimal number of clusters (k) selected by maximizing the silhouette score. These compounds were then clustered at the optimal 'k' value and subsequently mapped back to their corresponding BGCs, forming a ground-truth clustering in BGC space. We then clustered all MIBiG BGCs at different BiG-SCAPE thresholds and compared them to the ground truth using the adjusted_rand_score and adjusted_mutual_ info_score (3 different methods) (Python library scikit-learn[22]). Both metrics measure agreement between two clustering solutions, with values close to 1 indicating high concordance, while 0 suggests no agreement. As shown in Supplementary Table 14, all metrics achieved their highest values at a BiG-SCAPE threshold of 0.5. Therefore, we adopted a threshold of 0.5 in our study for BiG-SCAPE-based clustering to identify the correct BiG-SLiCE threshold in the subsequent section

Supplementary Table 14 Comparison of clusterings produced at different threshold values for BiG-SCAPE with the ground truth based on compound similarity for data from MIBiG.

BiG-SCAPE threshold	adjusted_rand_score	adjusted_mutual_info_score (geometric)	adjusted_mutual_info_score (arithmetic)	adjusted_mutual_info_score (max)
0.1	0.111	0.15	0.147	0.081
0.2	0.231	0.272	0.268	0.161
0.3	0.317	0.371	0.368	0.248
0.4	0.375	0.439	0.437	0.327
**0.5**	**0.375**	**0.459**	**0.458**	**0.397**
0.6	0.292	0.424	0.424	0.39
0.7	0.263	0.396	0.394	0.321
0.8	0.244	0.378	0.375	0.286
0.9	0.229	0.358	0.354	0.263
1	0.226	0.355	0.351	0.259

We compared the BGC clustering results produced by BiG-SCAPE (ground truth) to identify which BiG-SLiCE threshold yields comparable GCF assignments. For this analysis, we used BGCs from our 153 newly sequenced *Myxococcota* genomes, publicly available *Myxococcota* BGCs from the antiSMASH database, and all MIBiG BGCs. These BGCs were clustered using BiG-SCAPE (threshold 0.5; ground truth; see above paragraph) and BiG-SLiCE across thresholds ranging from 0.1 to 0.9 (step size 0.1). Clustering similarity was quantified using the adjusted_rand_score and adjusted_mutual_info score as described above. As shown in Supplementary Table 15, BiG-SLiCE at a threshold of 0.5 produced the highest agreement with the BiG-SCAPE clustering (threshold 0.5). However, the difference between thresholds 0.4 and 0.5 was minimal, and because 0.4 is the default BiG-SLiCE threshold, we adopted 0.4 for clustering in the main analyses.

Supplementary Table 15 Comparison of clusterings produced at different threshold values for BiG-SLICE with the ground truth based on BiG-SCAPE clustering at threshold 0.5 for the same dataset.

BiG-SLICE threshold	adjusted_rand_score	adjusted_mutual_info_score (geometric)	adjusted_mutual_info_score (arithmetic)	adjusted_mutual_info_score (max)
0.1	0.116	0.202	0.195	0.110
0.2	0.196	0.362	0.355	0.223
0.3	0.298	0.526	0.520	0.378
0.4	0.366	0.600	0.598	0.501
**0.5**	**0.386**	**0.619**	**0.619**	**0.592**
0.6	0.344	0.602	0.602	0.562
0.7	0.085	0.490	0.482	0.370
0.8	0.017	0.374	0.350	0.232
0.9	0.017	0.339	0.289	0.176

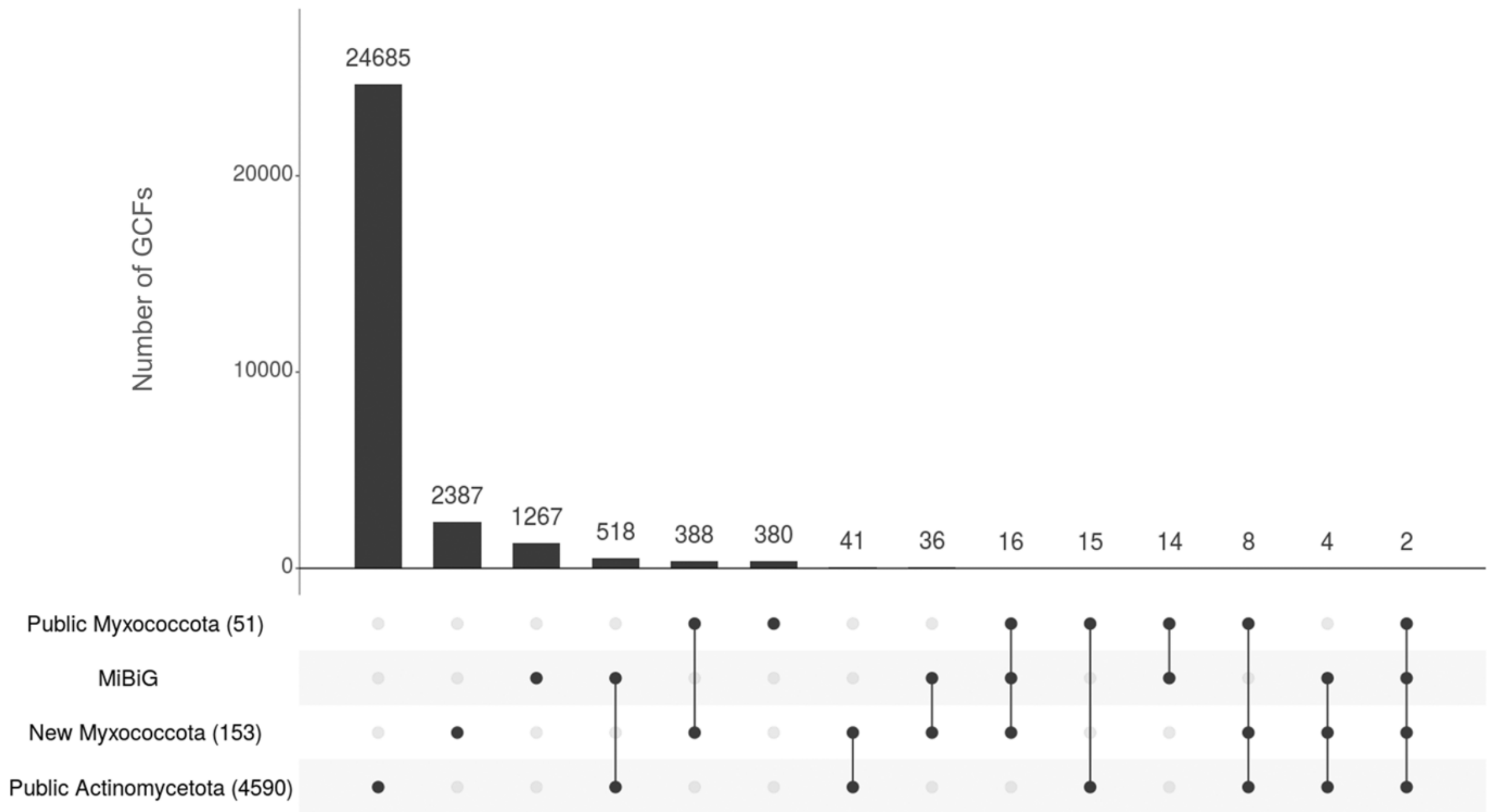

Supplementary Figure 52 Upset plot showing the number of unique and shared GCFs between different taxa and databases considered in this study.

Supplementary Note 8 Selection of *Actinomycetota* strains comparable to the in-house *Myxococcota* based on 16S DNA sequence.

The 16S rRNA sequences belonging to the phylum *Actinomycetota* were retrieved using NCBI BLAST[16], with three type strain sequences used as queries: Micromonospora (NR_026282), Streptomyces (NR_025663), and Actinomadura (NR_025344). This resulted in the retrieval of 16S rRNA sequences for 779 strains (having complete genomes) out of the 4,590 *Actinomycetota* strains with biosynthetic gene cluster (BGC) data available in the antiSMASH database[23]. In parallel, 16S sequences of 204 *Myxococcota* genomes were extracted from a local BLAST using Geneious Prime[24] (v2024.0.3).

The following tasks were performed using Galaxy's mothur toolsuite[25]. Both datasets were separately aligned based on the Silva SSU[26,27] (v138.1) database reference alignment and then trimmed. This resulted in 16S sequence lengths between 1,405 and 1,481 nt equivalent to the *E. coli* K-12 reference 16S sequence variable regions V1 to V9. With the processed sequences, we built separate *Myxococcota* and *Actinomycetota* distance matrices to establish sequence identities between the strains. A mothur clustering analysis (OptiClust, precision=0.001, metric=mcc, initial randomization=singleton, max number of iterations=100) was then performed using the distance matrices, which grouped each dataset's strains into families (94.1 % 16S RNA sequence identity), genera (96.3 % sequence identity), and species (98.6 % sequence identity) at 16S phylogenetic resolution.

Based on the phylogenetic diversity of 204 *Myxococcota* strains dataset, 204 out of the 779 *Actinomycetota* strains were selected to produce a dataset with matched diversity. Given its historical value in drug discovery, strains from the family *Streptomyteceae* were prioritized to match with the most represented *Myxococcota* family (*Myxococcaceae*). The remaining taxa were impartially selected in order to replicate the *Myxococcota* dataset's phylogenetic diversity.

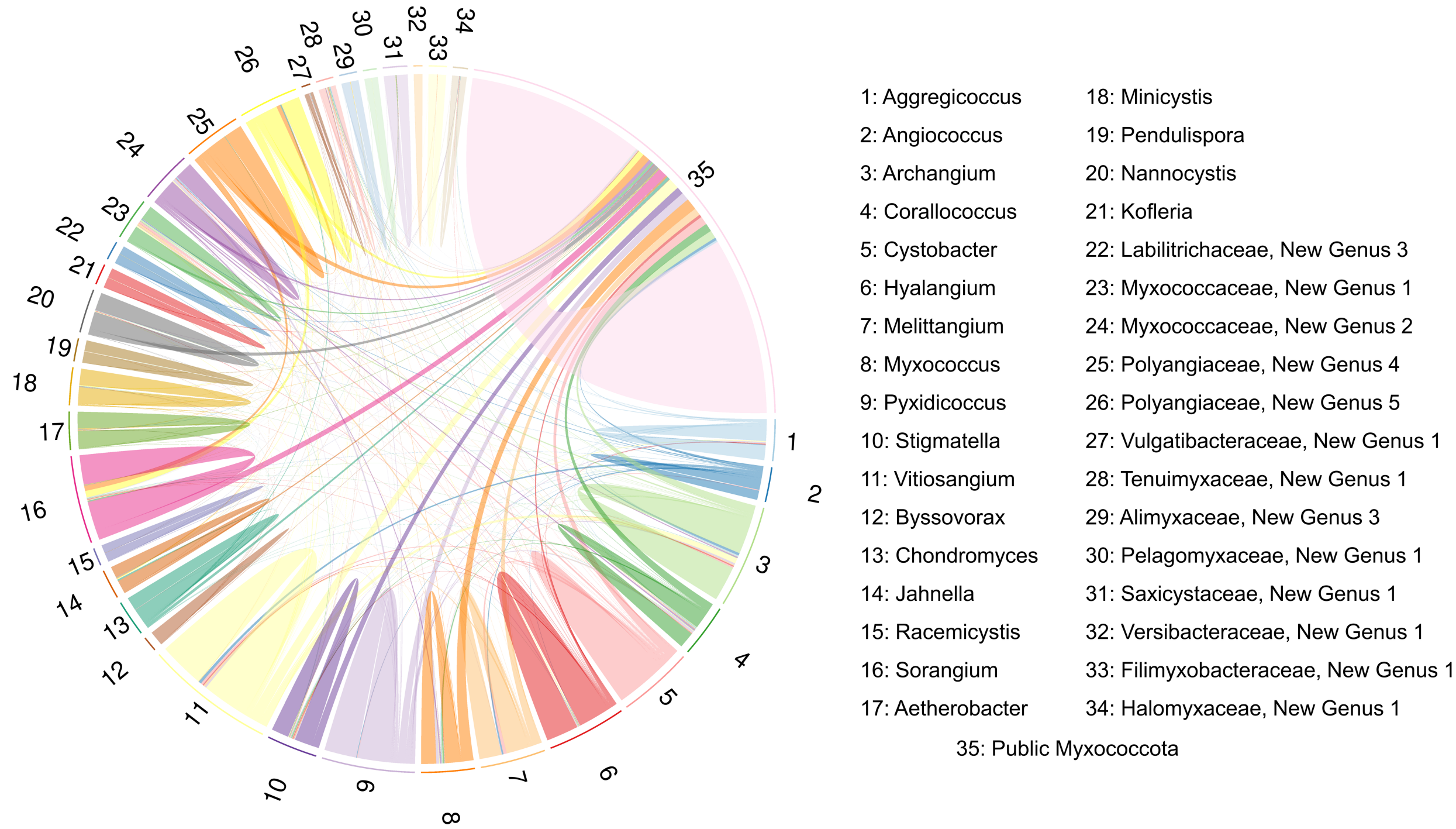

Supplementary Figure 53 Ribbon plot showing the overlap of GCFs between different genera (comprising at least two strains) in *Myxococcota* and public *Myxococcota* genomes.

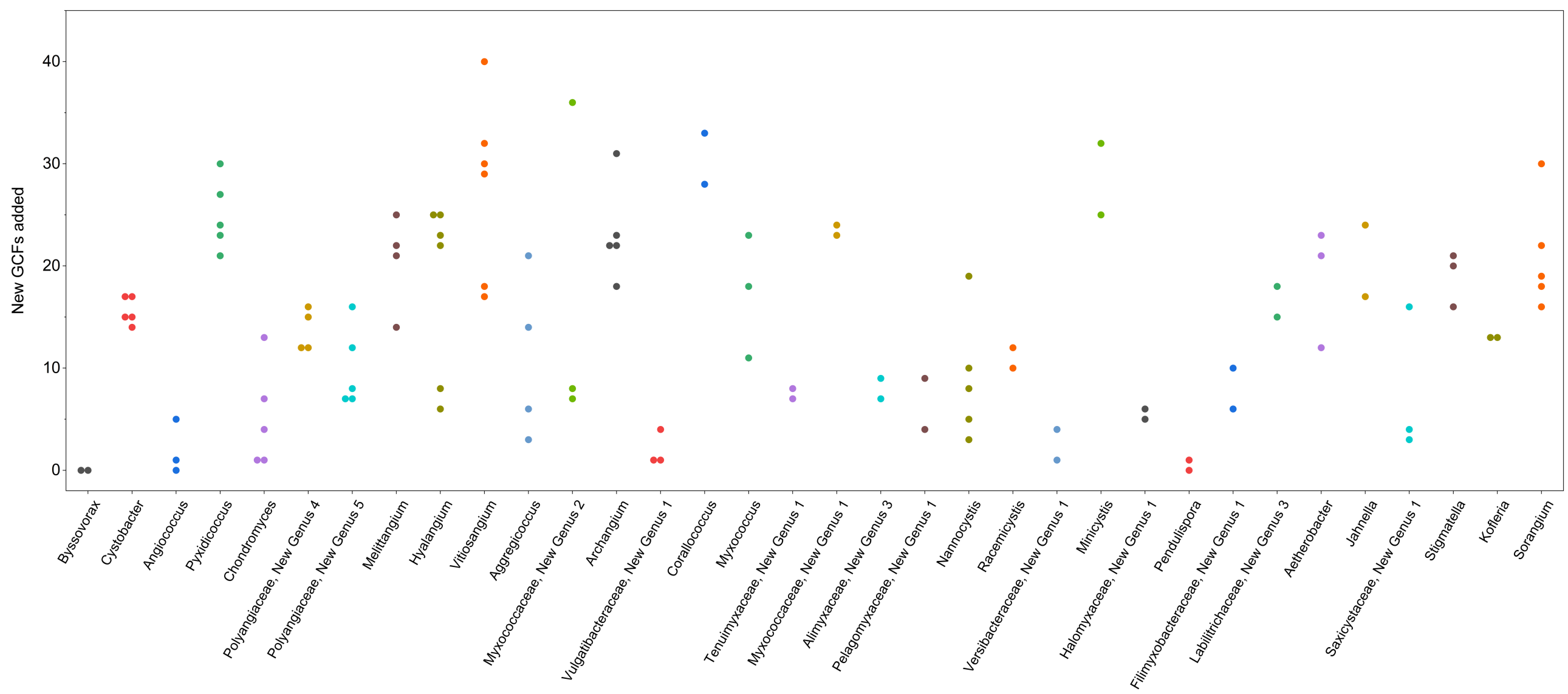

Supplementary Figure 54 Swarm plot showing the number of new GCFs added by individual strains in each genus. Only genera with at least two strains considered.

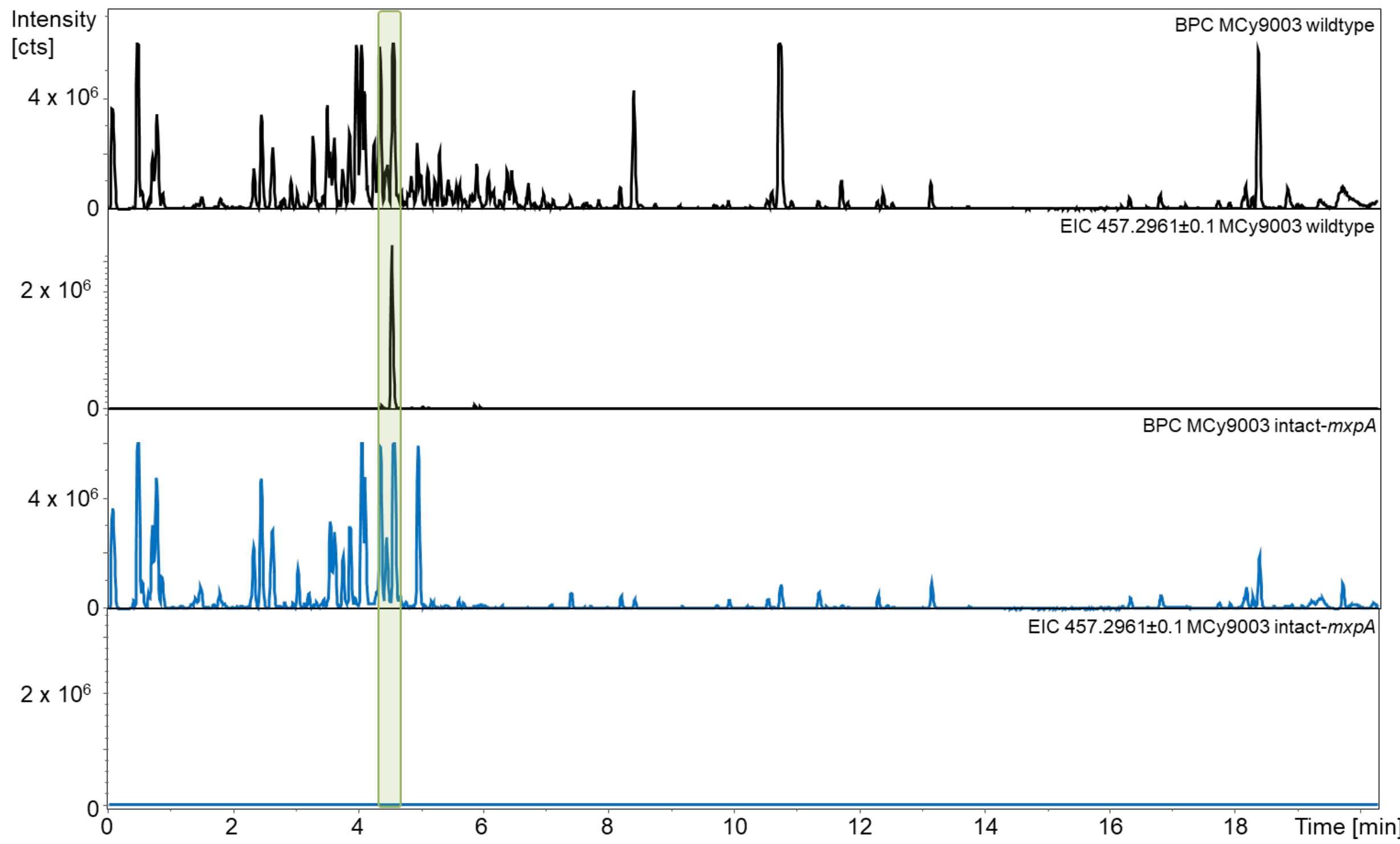

Supplementary Figure 55 Inactivation mutant of *mxpA*. Black chromatograms depict the base peak chromatogram (BPC) from MCy9003 wildtype extracts and the extracted ion chromatogram (EIC) at 457.2961 for highlighting the myxopentacin peak observed in wildtype extracts, blue chromatograms show BPC and EIC in the inactivation MCy9003 intact-*mxpA* mutant.

Supplementary Note 9 Structure elucidation of myxopentacin A.

HRESIMS data of myxopentacin A showed an intense signal for the double charged ion $[M+2H]^{2+}$ at *m/z* 457.297 (Supplementary Figure 61) consistent with a sum formula of $C_{46}H_{78}N_{10}O_9$ (calc. 457.29711 measured 457.29713 Δ = 0.04 ppm). Analysis of the proton spectrum showed signals characteristic for a mostly peptidic structure (Supplementary Table 16, Supplementary Figure 57). Examination of the HSQC spectrum (Supplementary Figure 59) revealed a deshielded signal at δ_H 4.09 ppm, δ_c 61.3 ppm which could be assigned as α-position of valine using COSY and HMBC correlations (Supplementary Figure 57, 59) forming the C-terminal end of the peptide with the respective shift of the carbonyl at, δ_c 178.1 ppm indicating a free carboxylic acid. Neighboring to the assigned valine, we assigned a 5-amino-2-ethyl-8-guanidino-4-oxooctanoic acid (AGOOA) building block using characteristic chemical shifts and COSY as well as HMBC correlations: the deshielded signal at δ_H 4.43 ppm (H-7_{AGOOA}) indicative for an α-position, neighbors a ketone as indicated by HMBC correlations to a carbon resonance at δ_C 208.3 ppm (C-6_{AGOOA}) To the same carbon resonance, HMBC correlations can be observed from two methylene protons at δ_H 3.07 and 2.58 ppm (H-5_{AGOOA}). These two methylene protons are part of a spin system consisting of a methine group at δ_H 2.77 ppm (H-2_{AGOOA}) substituted by an ethyl function and connected to the C-terminal valine via a peptide bond. Further five deshielded proton signals at δ_H 4.36, 4.28, 4.30, 4.42 and 3.67 ppm could be assigned as β-protons of five cispentacin (APCP) moieties, adjacent to the AGOOA building block. MS^2 fragmentation data was used to support the structure determined via 2D NMR spectroscopy (Supplementary Figure 62), showing a repetitive chain of five ACPC units, followed by the AGOOA moiety and the C-terminal valine. The configuration of the amino acids was determined by Marfey´s analysis to be L-valine and 1*(R)*,2*(S)*-amino-cyclopentylcarboxylic acid (Supplementary Figures 63 and 64).

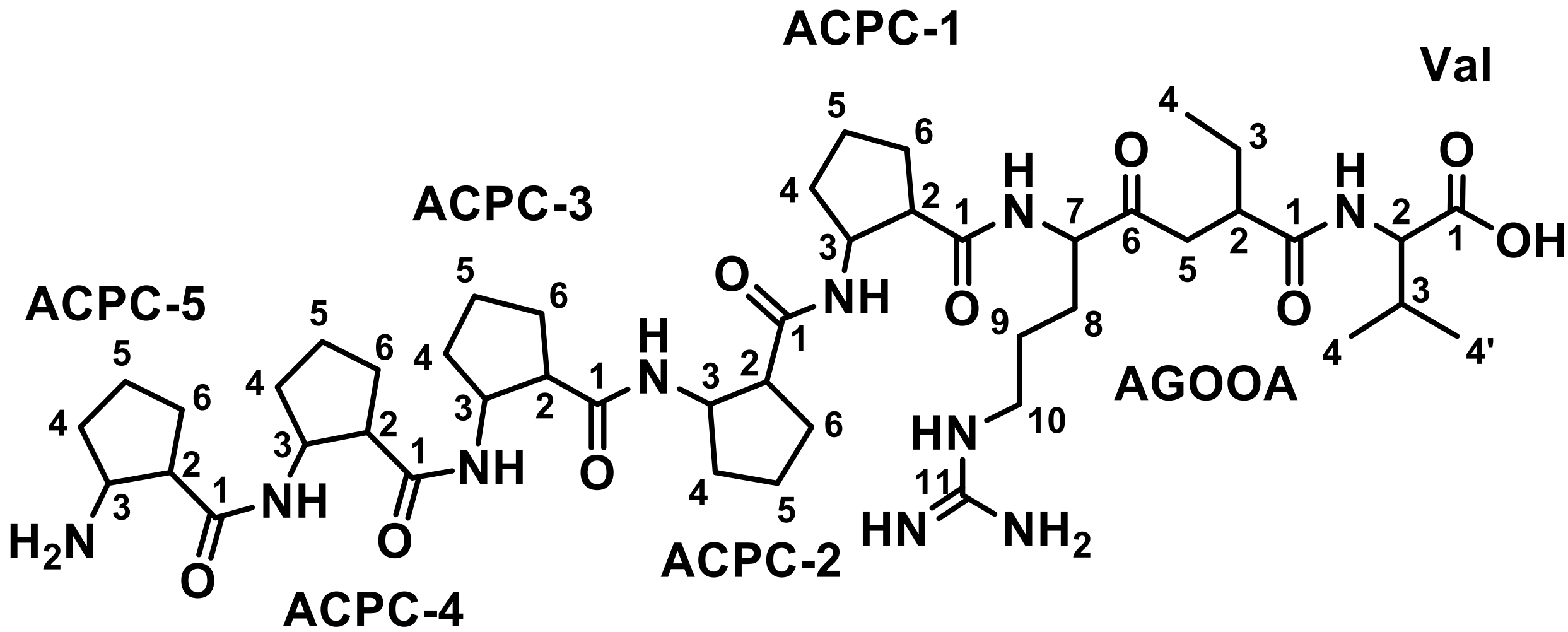

Supplementary Figure 56 Chemical structure of myxopentacin A with atom numbering used for NMR-based structure elucidation.

Supplementary Table 16 NMR spectroscopic data of myxopentacin A in methanol-d_4 at 500/125 MHz.

#	δ ^{13}C [ppm]	δ ^{1}H [ppm], mult (*J* [Hz])	COSY	HMBC
**Val**				
1	178.1	-	-	-
2	61.3	4.09, bd (5.6)	3	1, 3, 4, 4', AGOOA-1
3	32.2	2.08, m	2, 4, 4'	2, 4, 4'
4	19.8	0.93*	3, 4'	2, 3, 4'
4'	18.3	0.92*	3, 4	2, 3, 4
**AGOOA**				
1	176.3	-	-	-
2	44.1	2.77*	3, 5	3, 4
3	26.7	1.64, 1.51*	2, 4	1, 2, 3, 4, 5
4	11.7	0.96, t (7.5)	3	2, 3
5	42.6	3.07, dd (18.3, 10.9), 2.58, dd (18.3, 3.6)	2	1, 2, 3, 6
6	208.3	-	-	-
7	58.4	4.43*	8	6, 8, 9, ACPC-1
8	27.6	1.58, 1.85*	7, 9	7, 9
9	25.1	1.49*	8, 10	-
10	41.6	3.17, bt (6.5)	9	7, 8, 11
11	158.3	-	-	-
**ACPC-1**				
1	175.2	-	-	-
2	49.1	2.91, bdd (15.4, 8.0)	3, 6	1, 3, 6
3	53.6	4.36, bdd (13.4, 6.5)	2, 4	2, 4, 5, 6, ACPC-2

4	32.9	1.65, 1.88*	3, 5	3, 5, 6
5	23.4	1.56, 1.86*	4, 6	2, 4, 6
6	28.5	1.82, 1.97*	5, 2	2, 3, 5
**ACPC-2**				
1	174.7	-	-	-
2	49.1	2.83*	3, 6	1, 3, 4, 5, 6
3	53.6	4.28*	2, 4	1, 2, 6, ACPC-3
4	32.9	1.65, 1.92*	3, 5	3, 5
5	23.4	1.56, .1.86*	4, 6	2, 4, 6
6	28.5	1.82, 1.97*	5, 2	1, 2, 3, 5
**ACPC-3**				
1	174.5	-	-	-
2	49.1	2.83*	3, 6	1, 3, 4, 5, 6
3	53.6	4.30*	2, 4	1, 2, 4, ACPC-4
4	32.9	1.65, 1.92*	3, 5	3, 5
5	23.4	1.56, .1.86*	4, 6	2, 4, 6
6	28.5	1.82, 1.97*	5, 2	1, 2, 3, 5
**ACPC-4**				
1	174.5	-	-	-
2	49.1	2.83*	3, 6	1, 3, 4, 5, 6
3	53.5	4.42, bdd (13.4, 7.4)	2, 4	2, 4, ACPC-5
4	32.9	1.65, 1.92*	3, 5	3, 5
5	23.4	1.56, .1.86*	4, 6	2, 4, 6
6	28.5	1.82, 1.97*	5, 2	2, 3, 5
**ACPC-5**				
1	174.7	-	-	-
2	46.5	2.84*	3, 6	1, 2, 6
3	54.4	3.67, bdd (11.0, 6.6)	2, 4	2, 4, 5, 6
4	31.8	1.80, 2.10*	3, 5	1, 3, 5
5	22.6	1.74, 1.91*	4, 6	4, 6
6	29.6	1.87, 2.04*		2, 5

* no assignment possible due to overlapping signals

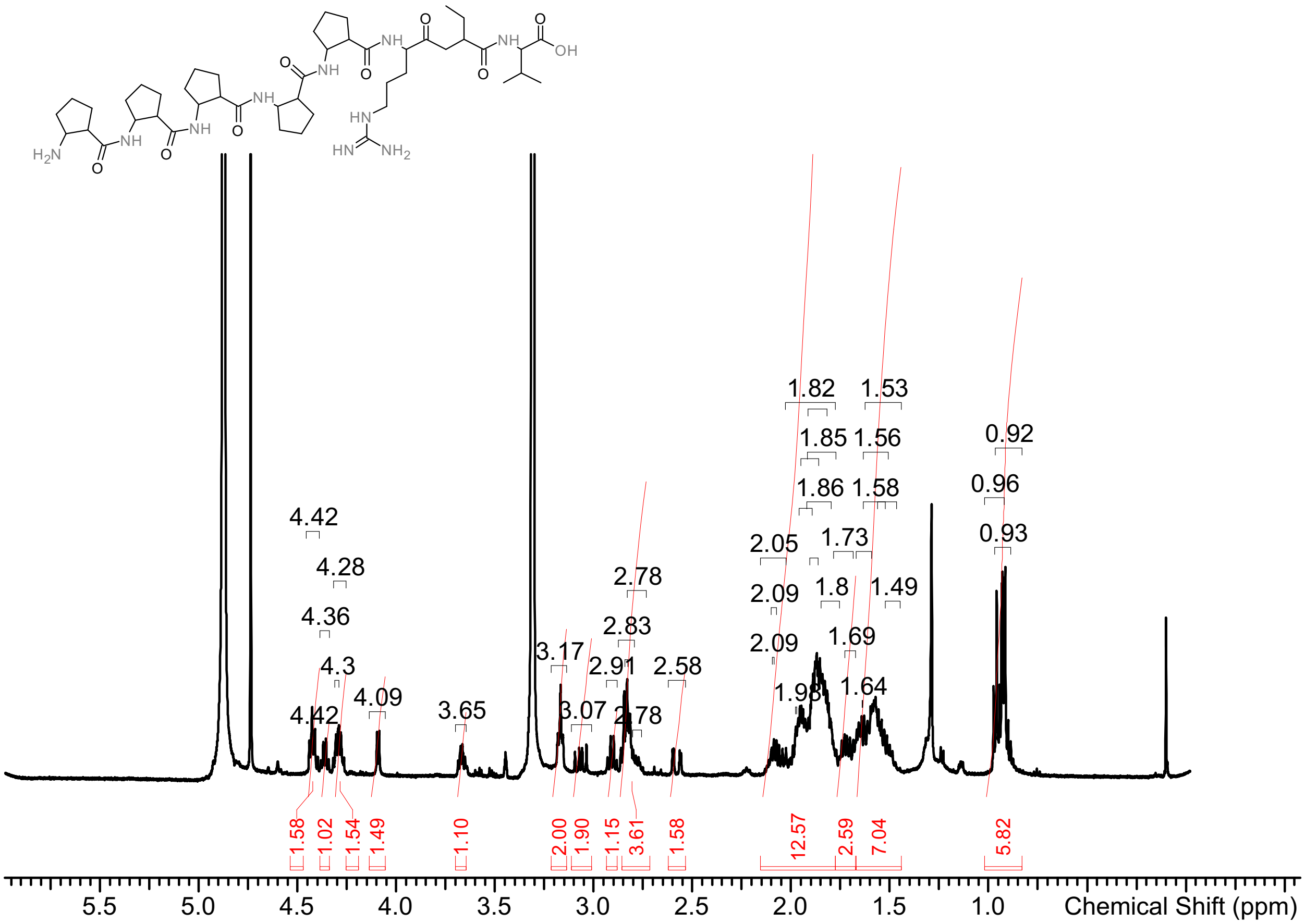

Supplementary Figure 57 ^{1}H-spectrum of myxopentacin A in MeOD-d_4 at 500 MHz.

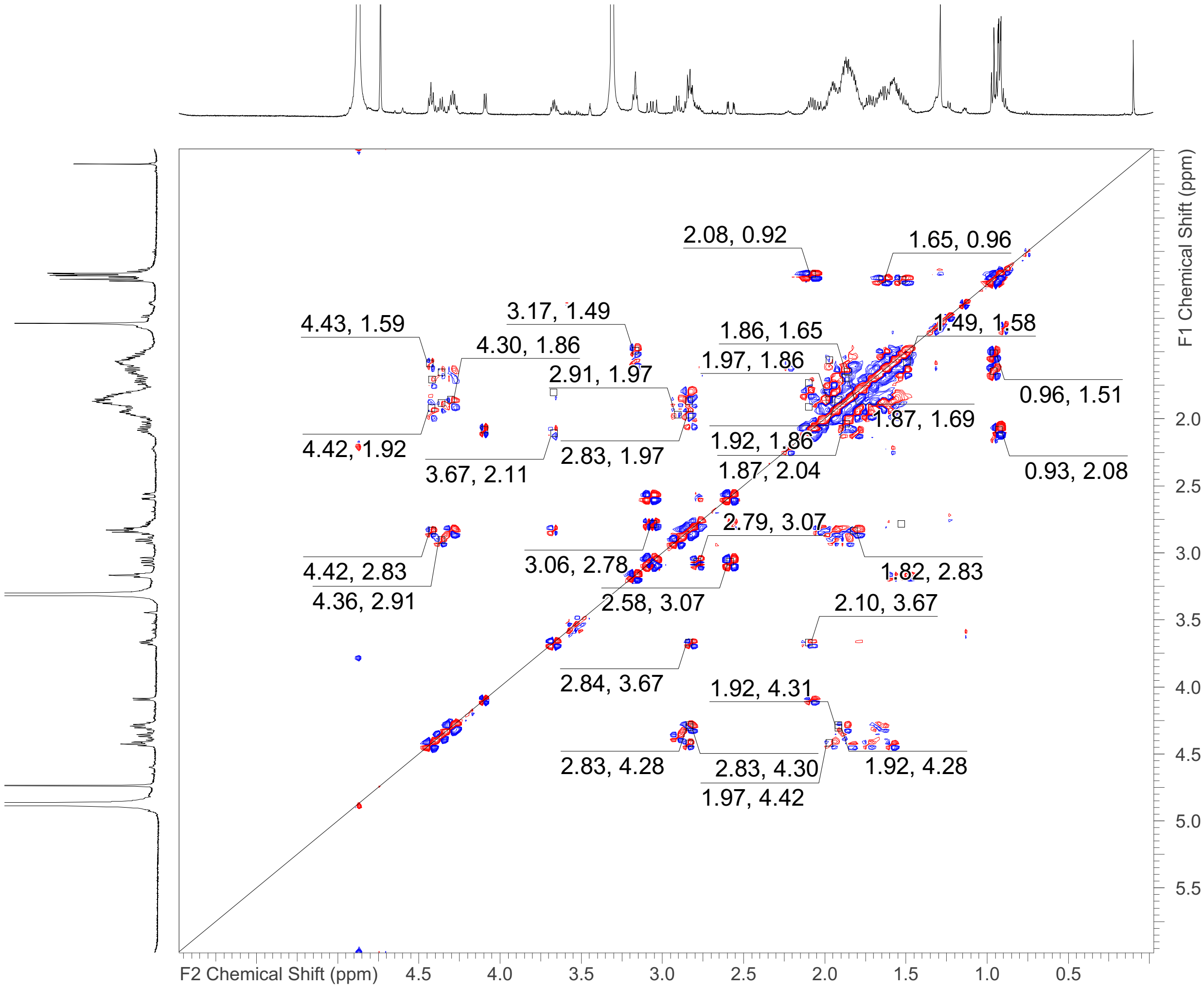

Supplementary Figure 58 COSY-spectrum of myxopentacin A in MeOD-d_4 at 500 MHz.

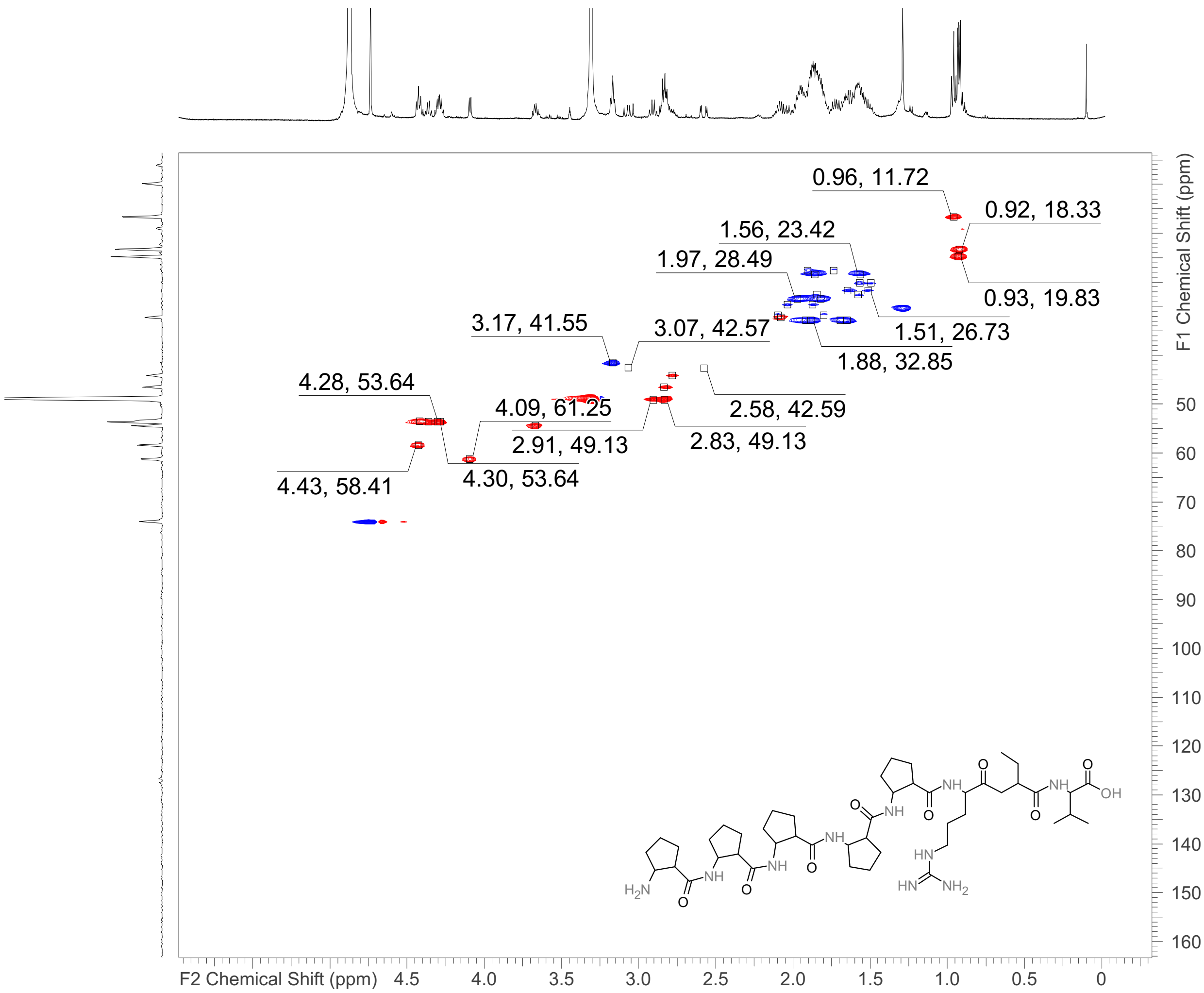

Supplementary Figure 59 HSQC-spectrum of myxopentacin A in MeOD-d_4 at 500 MHz/125 MHz.

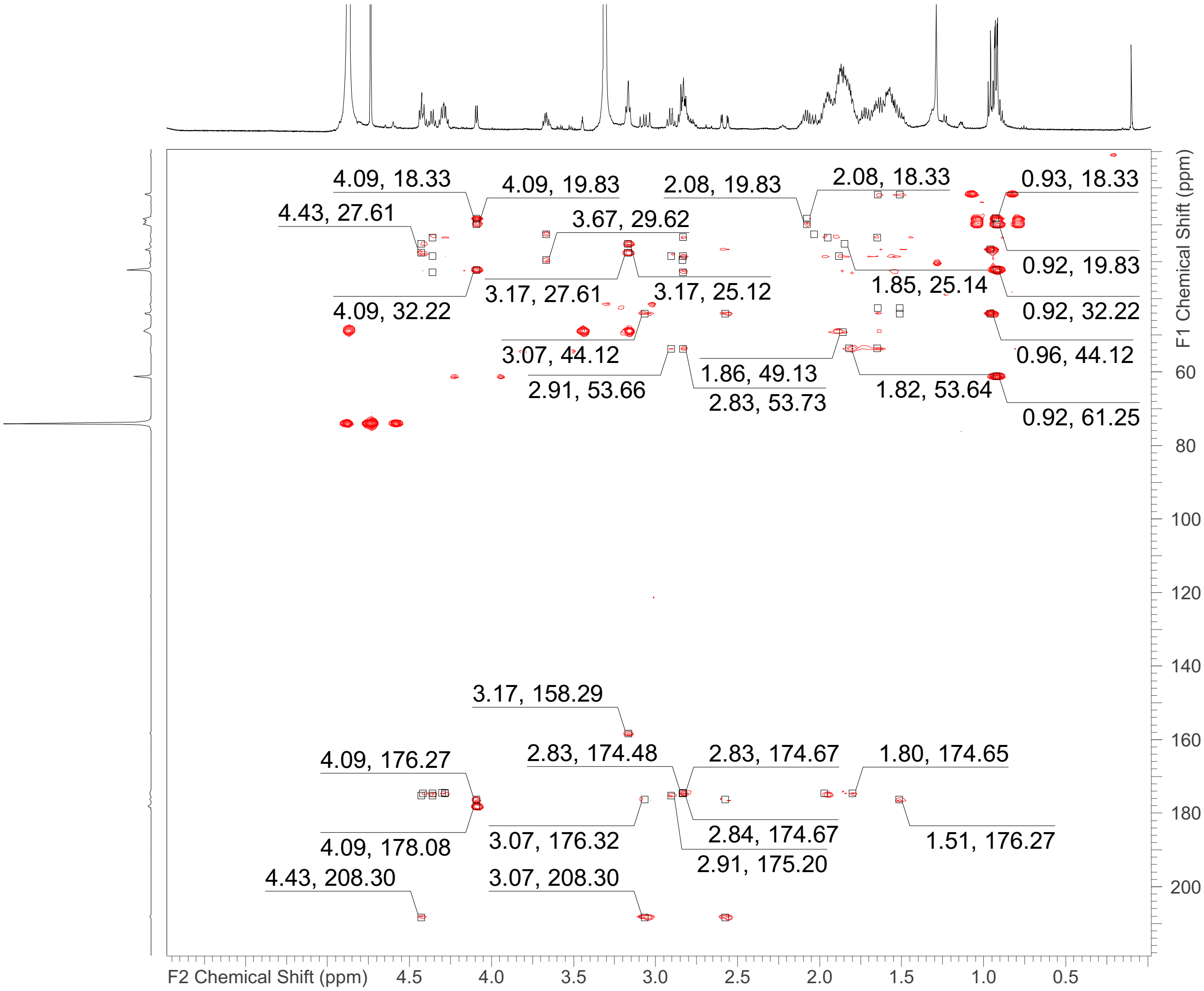

Supplementary Figure 60 HMBC-spectrum of myxopentacin A in MeOD-d_4 at 500 MHz/125 MHz.

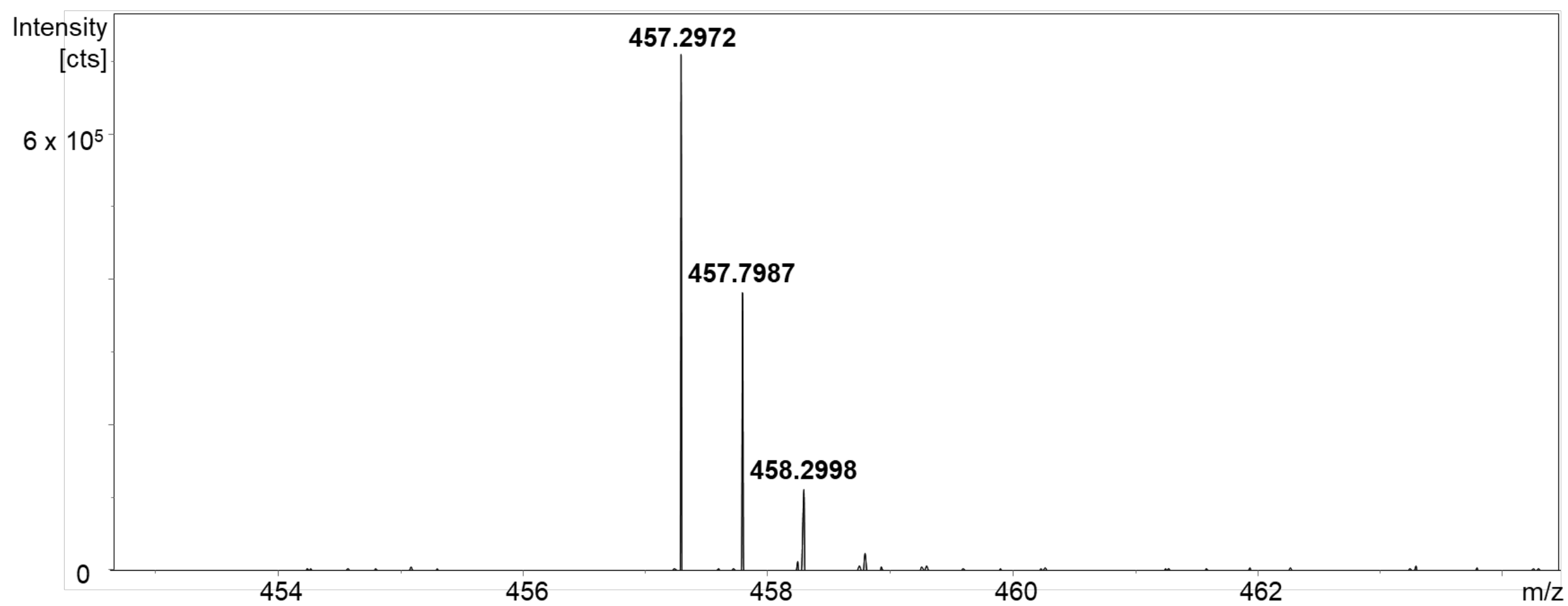

Supplementary Figure 61 MS spectrum for myxopentacin A.

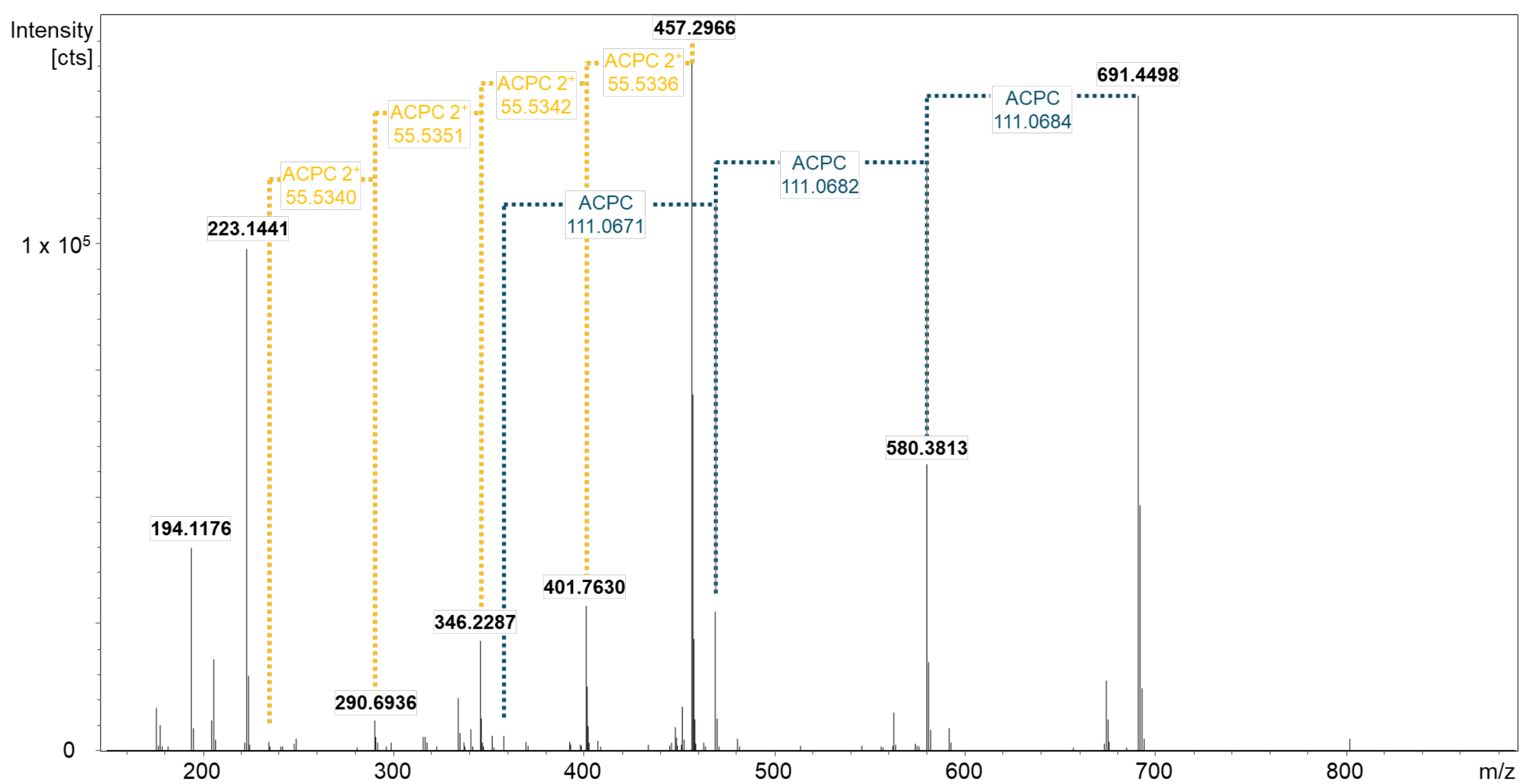

Supplementary Figure 62 MS^2 fragment spectrum for myxopentacin A showing characteristic ACPC losses in both the $[M+H]^+$ (blue) and $[M+2H]^{2+}$ (yellow) series supporting the NMR-assigned structure.

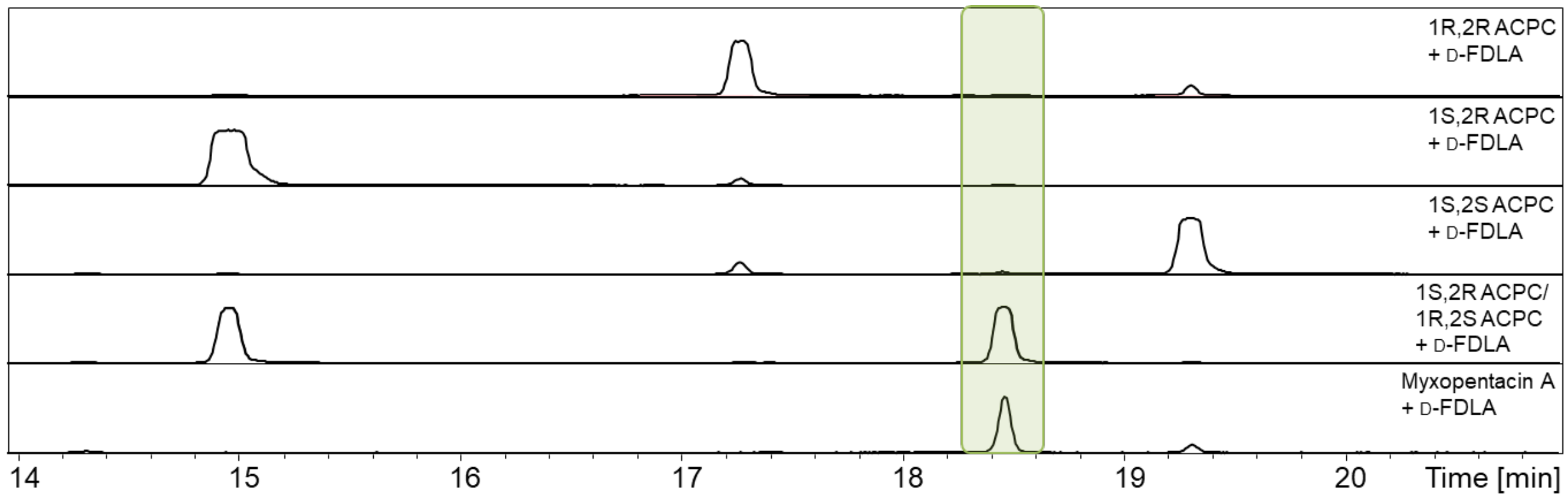

Supplementary Figure 63 Marfey's derivatization of reference ACPC (cispentacin) with D-FDLA and L-FDLA and myxopentacin A with D-FDLA for retention time comparison resulted in assignment of 1R, 2S ACPC incorporation in myxopentacin A.

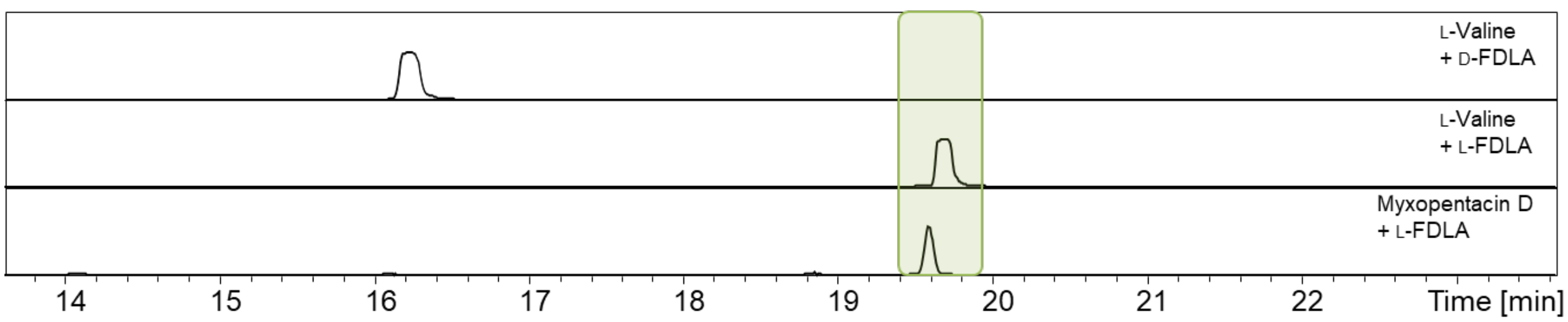

Supplementary Figure 64 Marfey's derivatization of reference L-valine with D-FDLA and L-FDLA and myxopentacin A with with L-FDLA for retention time comparison resulted in assignment of L-valine incorporation in myxopentacin A.

Supplementary Note 10 Biosynthesis of myxopentacin.

Making use of the NMR-based myxopentacin A structure, the minimal boundaries of the *mxp*BGC in MCy9003 were defined to span 52.2 kb that encode 21 genes named *mxpA*-*mxpU* (Supplementary Figure 65A, Supplementary Table 17). Beyond *mxpA-H*, the cispentacin cassette, several additional assignments can be made (Supplementary Figure 65, Supplementary Table 17). MxpA encodes a two modular NRPS with the NRPS A domain code (DPWFLGAVFK) of M01 aligning well with the observed valine incorporation (best match = valine; PARASECT[28] prediction 0.861). The NRPS A domain code of M01 (DPWFVGGVFK) suggests incorporation of an aliphatic amino acid as well with the three best hits valine, leucine and isoleucine (PARASECT prediction 0.845, 0.825, 0.787, respectively) aligning with the observed aminobutyric acid incorporation (Supplementary Figure 65B). Adjacent to *mxp*B-H, the cispentacin cassette, are *mxpJ*, *mxpK* and *mxpL*, which show significant homologies to genes in the ketomemicin pathway[29]. MxpJ shares homology (50% aa identity) with KtmA, the aldolase catalyzing an aldol reaction concomitant with decarboxylation starting from malonyl-CoA and phenylpyruvate during ketomemicin formation. We therefore expect MxpJ to catalyze a similar reaction on the dipeptide generated by MxpA after conversion of the terminal amine to a keto-function (Supplementary Figure 65B), but were unable to pinpoint the enzyme performing the required transamination reaction. MxpL shares homology (62% aa identity) with KtmC, the dehydratase reducing -OH group generated by the aldolase reaction and is therefore expected to perform a similar reaction on the building block generated by MxpJ in myxopentacin biosynthesis. *MxpB* encodes six NRPS modules aligning well with the observed incorporation of five ACPC and one arginine building block in myxopentacin A (Supplementary Figure 65C). However, the function of the encoded PKS module (M03) remains elusive. The NRPS A domain code of M04 and M06 (DVWHVSLVDK) both have serine as a best matching hit in PARASECT (1.000) aligning with the fact that serine and ACPC both show hydrophilic substituents in β-position. Interestingly, the NRPS A domain codes in M05, M07 and M08 differ from M04 and M06 (DILQLGLVWK, DAWGQAFIDK and DAFFLGFTYK, respectively) without any good matches of their predicted specificities to the observed ACPC incorporation. The NRPS A domain code of M09 (DAEDIGTVVK) suggests incorporation of an alkaline amino acid with best hits being lysine, ornithine or arginine (PARASECT prediction 0.938, 0.827, 0.797, respectively) aligning well with the observed arginine incorporation. Lastly, MxpK shares homology (59% aa identity) with KtmB, a PLP-dependent amino acid C-acyltransferase catalyzing the Claisen-type condensation between phenylalanine and benzylfumaryl-CoA in ketomemicin and expected to perform a similar reaction on the two building blocks generated from MxpA + MxpJ + MxpL and MxpU + MxpK to form the final myxopentacin A.

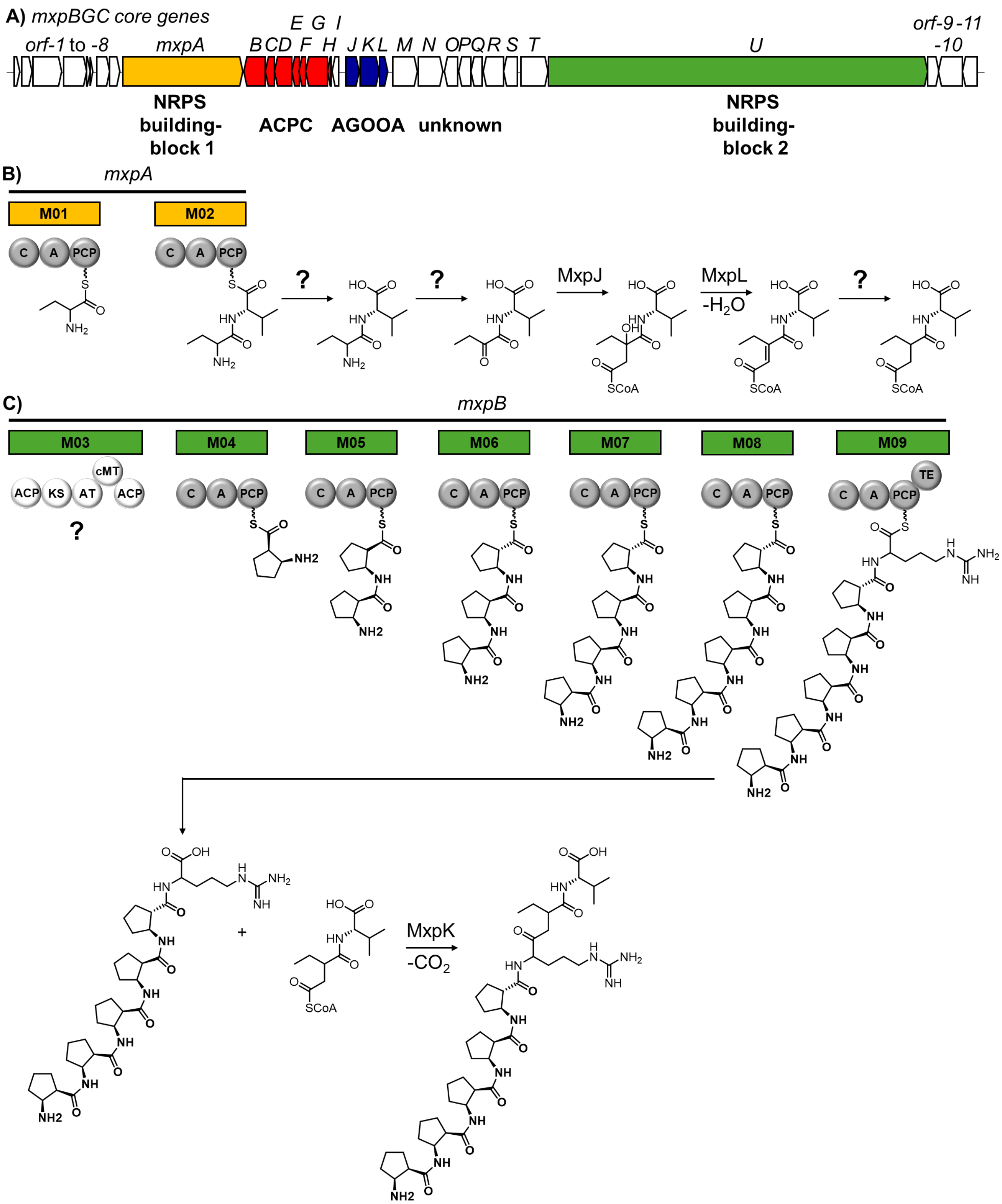

Supplementary Figure 65 *mxp* BGC from *Myxococcus* MCy9003 with proposed functions of the enzymes encoded by the NRPS core and some of the accompanying genes. **A** Besides the cispentacin cassette used for genome mining (*mxpB-H*), the minimal *mxp*BGC includes two NRPS cassettes (*mxpA*, yellow and *mxpU* green) as well as a three gene cassette (*mxpJ-L*) likely involved in formation of the AGOOA building block observed in myxopentacin A. White = Genes encoding proteins with unknown function. **B** The C-terminal end of myxopentacin A is likely initiated by the two modules of *mxpA*, with subsequent elongation by MxpJ and L. **C** The six modules of *mxpU* are predicted to form the cispentacin chain with a final arginine elongation before release from the assembly line and coupling to the building block shown in **B**.

Supplementary Table 17 Genes detected in the *mxp*BGC from *Myxococcus* MCy9003 with their closest protein homologues.

Name	Size [AA]	Proposed function/ homologue	Organism	Accession number of closest protein homologue	Sequence identity [%]
mxpA	2593	NRPS, Gramicidin S synthase 2	*Brevibacillus brevis*	P0C064.2	37%
mxpB	469	2-succinyl-benzoate-CoA ligase	*Bacillus licheniformis*	Q65FT5.1	24%
mxpC	194	Phosphoribosylformimino-5-aminoimidazole carboxamide ribotide isomerase	*Rippkaea orientalis*	B7K3A9.1	26%
mxpD	374	Beta-ketoacyl-ACP synthase	*Mycobacterium tuberculosi*	H8ESN0.2	42%
mxpE	174	Coronafacic acid dehydratase	*Pseudomonas savastanoi*	P72238.1	53%
mxpF	148	Acyl-CoA thioesterase YbgC	*Haemophilus influenzae*	P44679.1	29%
mxpG	465	Ornithine aminotransferase	*Brevibacillus brevis*	C0ZBR4.1	29%
mxpH	89	D-alanyl carrier protein	*Streptococcus sanguinis*	A3CR85.1	40%
mxpI	153	Cys-tRNA(Pro)/Cys-tRNA(Cys) deacylase YbaK	*Haemophilus influenzae*	P45202.2	31%
mxpJ	282	Citrate lyase subunit beta	*Klebsiella pneumoniae*	P17725.2	32%
mxpK	410	8-amino-7-oxononanoate synthase	*Marinomonas sp.*	A6W0Y0.1	27%
mxpL	178	Mesaconyl-CoA hydratase	*Haloarcula marismortui*	Q5V464.2	30%
mxpM	529	4-amino-L-phenylalanyl-[CmlP-peptidyl-carrier-protein] 3-hydroxylase	*Streptomyces venezuela*	F2RB80.1	38%
mxpN	534	4-amino-L-phenylalanyl-[CmlP-peptidyl-carrier-protein] 3-hydroxylase	*Streptomyces venezuelae*	F2RB80.1	32%
mxpO	293	CAAX prenyl protease 2	*Methanococcus maripaludis*	Q6LZY8.1	27%

mxpP	272	Aspartyl/asparaginyl beta-hydroxylase	*Homo sapiens*	Q12797.3	40%
mxpQ	243	Methyltransferase sdnD	*Sordaria araneosa*	A0A1B4XBG9.1	26%
mxpR	435	Macrolide efflux protein A	*Streptococcus pyogenes*	P95827.1	26%
mxpS	305	2-oxoglutarate and iron-dependent oxygenase JMJD	*Gallus gallus*	Q5ZHV5.1	24%
mxpT	592	Medium-chain-fatty-acid-[acyl-carrier-protein] ligase JamA	*Moorena producens*	Q6E7K9.1	50%
mxpU	8206	NRPS, Tyrocidine synthase 3	*Brevibacillus parabrevis*	O30409.1	37%

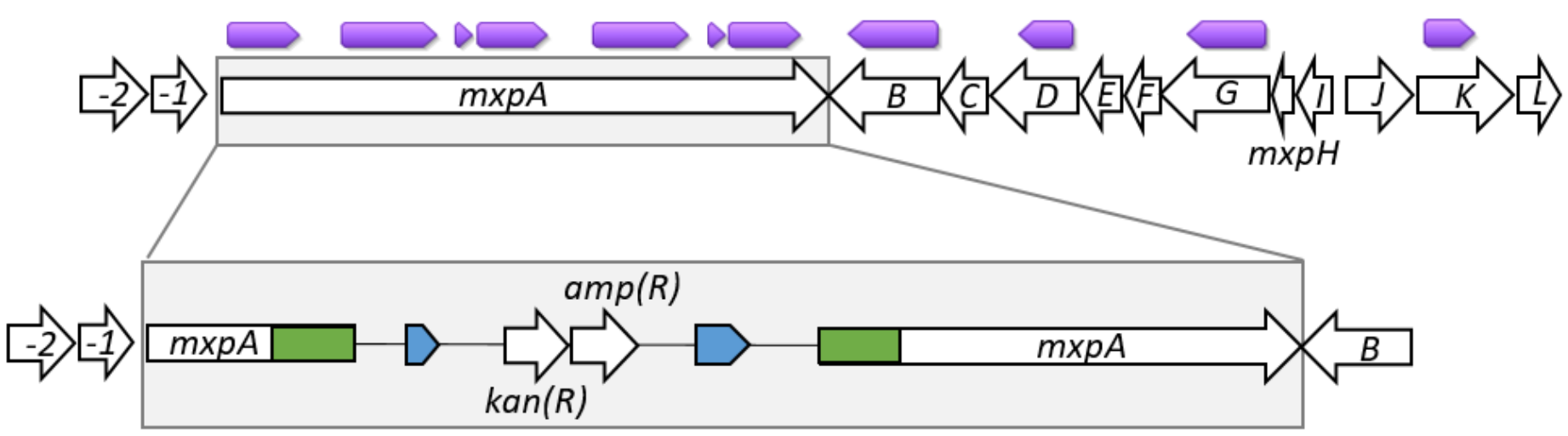

Supplementary Figure 66 Inactivation of mxpA. The plasmid pCR2.1-001inact39Ao1 was inserted into mxpA via homologous recombination using a PCR amplified fragment (001inact39A) from MCy9003 genomic DNA using 001-inact-nrps39A-F and 001-inact-nrps39A-R primers (see Supplementary Table 18). The orientation of the homology region in a plasmid was determined using PCR21R in combination with 001-inact-nrps39A-F and 001-inact-nrps39A-R primer (Supplementary Table 18). For confirmation of the homology region in the plasmid, the primer G001-inact39Ao1 (Supplementary Table 18) aligning in the vicinity of the homology region was used. Homology arms shown in green, origins of replication in blue and selection markers for ampicillin and kanamycin labelled as *kan(R)* and *amp(R).* Catalytic domains for the genes mxpA-mxgL are shown in purple.

Supplementary Table 18 Plasmids and oligonucleotides used in this study.

Plasmid name	Details	Reference
pCR2.1-TOPO	ori(pUC), ori(f1), kanamycin resistance (kanR), ampicillin resistance (ampR)	TOPO cloning kit
pCR2.1-001inact39A	1 kb homology from the first A domain of mxpA gene cloned into pCR2.1 in forward orientation	This study
pSBtn5Kan	ori(pUC), ori(f1), kanR, ampR, P*tn5* – for gene activation by single homologous recombination, homology arm is inserted through *Nde*I and *Eco*RI restriction sites	Panter et al.[30]
pSBtn5Kan001nrps12	1.2 kb fragment from the start of MCy9003_22370 gene cloned into pSBtn5Kan	This study
**Primer**	**5'-Sequence-3'**	**Construct/Usage**
001-inact-nrps39A-F	GTGTCGGGCGCTCGGTC	pCR2.1 001inact39Ao1
001-inact-nrps39A-R	CACGGATCTTCACCTGGAGGTC	
PCR21R	ATAGGGCGAATTGGGCCCT	Reverse primer on the backbone of pCR2.1 vector
G001-inact39Ao1	CCATCTCCAGCGCGATGAC	Aligning on the genome located in front of pCR2.1-000inact39Ao1 plasmid
001nrps12-NdeI-F	ATATA<u>CATATG</u>GTGCCGGTGCCCC	pSBtn5Kan001nrps12
001nrps12-EcoRI-R	ATATA<u>GAATTC</u>GAGAGGTCGTTCCTCGC CACAC	
g001-12F	CTCCCACTCGACATGAA	Alignment on the genome, outside of 001nrps12 homology
g001-12R	GTCGAAGACATCGGTGTTGTA	
M13-21F	TGTAAAACGACGGCCAGT	For general plasmid integration verifications
M13-24R	CGGATAACAATTTCACACAGG	

*Restriction enzyme sites are underlined

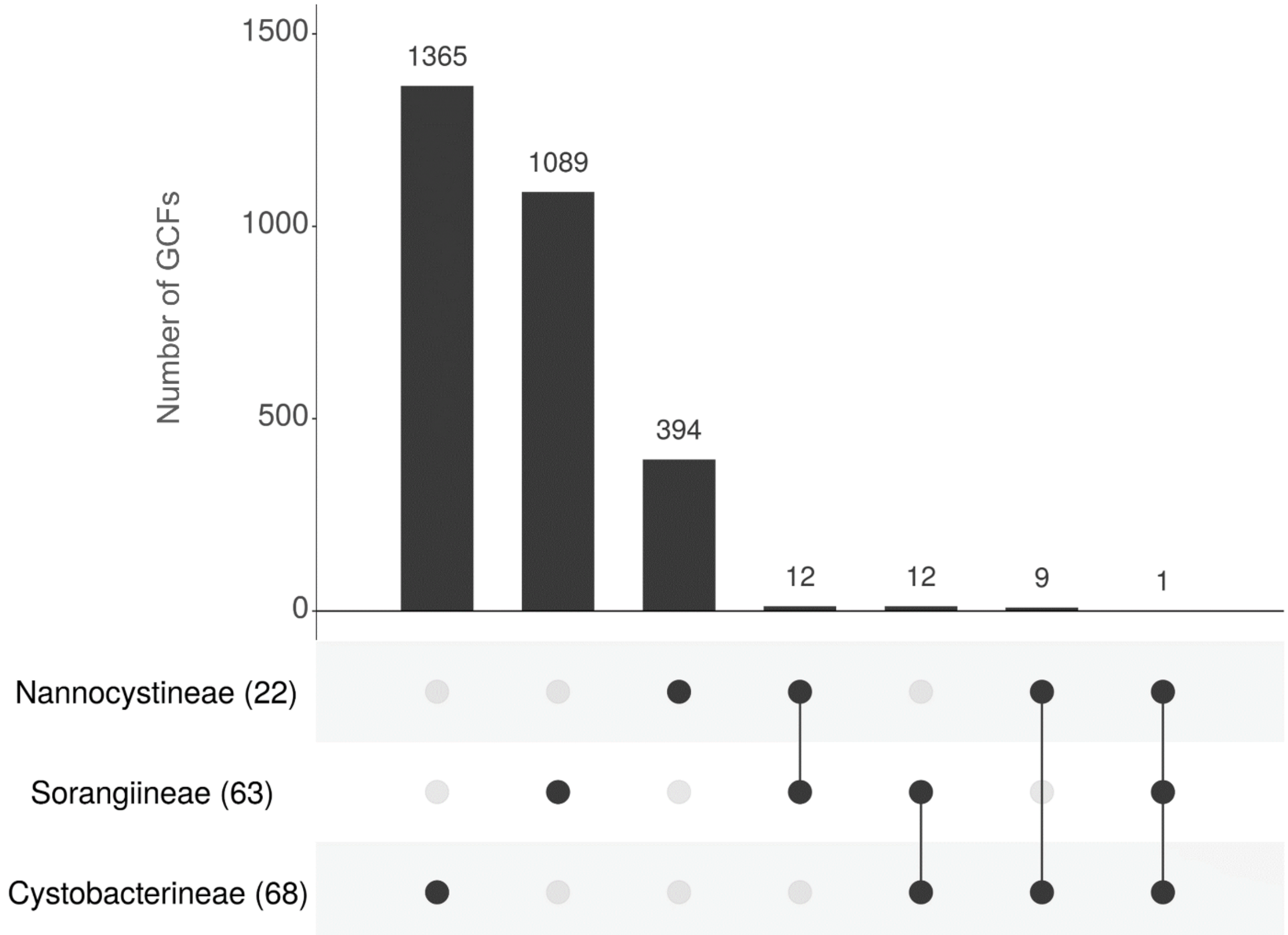

Supplementary Figure 67 Upset plot showing the number of unique and shared GCFs between different sub-orders belonging to *Myxococcota* phylum.

Supplementary Table 19 BioProject, BioSample and Assembly accessions of strains submitted at NCBI

Strain	Alias	BioProject	BioSample	Assembly
Byc1	Byc1	PRJNA1405458	SAMN54742344	JBXDQN000000000
Byc2	SBBy001	PRJNA1405458	SAMN54742345	JBXDQM000000000
Cbb2	SBCb32	PRJNA1405458	SAMN54742346	JBWQDA000000000
Cbfe18	Cb029	PRJNA1405458	SAMN54742347	JBWQCZ000000000
Cbm10	Cbm10	PRJNA1405458	SAMN54742348	JBWQCY000000000
Ccc1071	Ccc1071	PRJNA1405458	SAMN54742349	JBWQCX000000000
Cma13	Cma13	PRJNA1405458	SAMN54742350	JBWQCW000000000
Cmp51	SBCm020	PRJNA1405458	SAMN54742351	JBXDQL000000000
DSM105741	Soce375	PRJNA1405458	SAMN54742352	JBWQCV000000000
DSM105767	Soce362	PRJNA1405458	SAMN54742353	JBWQCU000000000
DSM105769	Soce1828	PRJNA1405458	SAMN54742354	JBWQCT000000000
DSM14627	Soce1871	PRJNA1405458	SAMN54742355	JBWQCS000000000
DSM14710	SBCb025	PRJNA1405458	SAMN54742356	JBWQCR000000000
DSM14712	Sbcb031	PRJNA1405458	SAMN54742357	JBWQCQ000000000
DSM14718	Cbv34	PRJNA1405458	SAMN54742358	JBWQCP000000000
DSM14724	NOCB2	PRJNA1405458	SAMN54742359	JBWQCO000000000
DSM14727	SBCb026	PRJNA1405458	SAMN54742360	JBXDQP000000000
DSM14751	SBCb027	PRJNA1405458	SAMN54742361	JBWQCN000000000
DSM14875	SBPl001	PRJNA1405458	SAMN54742362	JBWQCM000000000
DSM14877	SBMe006	PRJNA1405458	SAMN54742363	JBWQCL000000000
DSM15217	DSM15217	PRJNA1405458	SAMN54742364	JBWQCK000000000
DSM21377	DSM21377	PRJNA1405458	SAMN54742365	JBWQCJ000000000
DSM53252	Soce176	PRJNA1405458	SAMN54742366	JBWQCI000000000
Hym3	Hym3	PRJNA1405458	SAMN54742367	JBWQCH000000000
MCy10583	SBCy006	PRJNA1405458	SAMN54742368	JBWQCG000000000
MCy10595	Sbcy010	PRJNA1405458	SAMN54742369	JBWQCF000000000
MCy10622	SBCy015	PRJNA1405458	SAMN54742370	JBXDQK000000000
MCy10635	SBCy017	PRJNA1405458	SAMN54742371	JBWQCE000000000
MCy10636	SBCy018	PRJNA1405458	SAMN54742372	JBWQCD000000000
MCy10644	Sbcy024	PRJNA1405458	SAMN54742373	JBWQCC000000000
MCy10650	Sbcy028	PRJNA1405458	SAMN54742374	JBWQCB000000000
MCy10943	SBCy046	PRJNA1405458	SAMN54742375	JBWQCA000000000
MCy10944	SBCy047	PRJNA1405458	SAMN54742376	JBWQBZ000000000
MCy11274	SBCy050	PRJNA1405458	SAMN54742377	JBWQBY000000000
MCy11668	SBAg003	PRJNA1405458	SAMN54742378	JBWQBX000000000
MCy11987	SBCy132	PRJNA1405458	SAMN54742379	JBWQBW000000000
MCy12023	SBAn005	PRJNA1405458	SAMN54742380	JBWQBV000000000
MCy12024	Sbcy101	PRJNA1405458	SAMN54742381	JBWQBU000000000
MCy12025	Sbcy131	PRJNA1405458	SAMN54742382	JBWQBT000000000
MCy12037	Sbcy157	PRJNA1405458	SAMN54742383	JBWQBS000000000
MCy12117	Sbcy167	PRJNA1405458	SAMN54742384	JBWQBR000000000
MCy12312	SBCy272	PRJNA1405458	SAMN54742385	JBWQBQ000000000
MCy12336	SBCy276	PRJNA1405458	SAMN54742386	JBWQBP000000000
MCy12490	SBCy326	PRJNA1405458	SAMN54742387	JBWQBO000000000
MCy12497	SBAg021	PRJNA1405458	SAMN54742388	JBWQBN000000000
MCy12563	Sbcy335	PRJNA1405458	SAMN54742389	JBWQBM000000000

MCy12575	SBAg023	PRJNA1405458	SAMN54742390	JBWQBL000000000
MCy12611	SBCc345	PRJNA1405458	SAMN54742391	JBWQBK000000000
MCy12684	SBAr054	PRJNA1405458	SAMN54742392	JBWQBJ000000000
MCy12689	SBAr059	PRJNA1405458	SAMN54742393	JBWQBI000000000
MCy12715	Sbcy387	PRJNA1405458	SAMN54742394	JBWQBH000000000
MCy12744	SBCy471	PRJNA1405458	SAMN54742395	JBWQBG000000000
MCy12793	SBCy461	PRJNA1405458	SAMN54742396	JBWQBF000000000
MCy12919	Sbcy509	PRJNA1405458	SAMN54742397	JBWQBE000000000
MCy12977	SBCy513	PRJNA1405458	SAMN54742398	JBWQBD000000000
MCy13058	SBCy547	PRJNA1405458	SAMN54742399	JBWQBC000000000
MCy13113	SBAr066	PRJNA1405458	SAMN54742400	JBWQBB000000000
MCy13173	SBCy606	PRJNA1405458	SAMN54742401	JBWQBA000000000
MCy13239	SBCy574	PRJNA1405458	SAMN54742402	JBWQAZ000000000
MCy13275	Sbcy693	PRJNA1405458	SAMN54742403	JBWQAY000000000
MCy13299	SBCy255	PRJNA1405458	SAMN54742404	JBWQAX000000000
MCy13300	Sbcy256	PRJNA1405458	SAMN54742405	JBWQAW000000000
MCy13314	Sbcc431	PRJNA1405458	SAMN54742406	JBWQAV000000000
MCy13507	SBMx457	PRJNA1405458	SAMN54742407	JBWQAU000000000
MCy1366	MCy1366	PRJNA1405458	SAMN54742408	JBWQAT000000000
MCy14112	SBCy730	PRJNA1405458	SAMN54742409	JBWQAS000000000
MCy14154	SBSr100	PRJNA1405458	SAMN54742410	JBWQAR000000000
MCy14232	Sbcy750	PRJNA1405458	SAMN54742411	JBWQAQ000000000
MCy14233	Sbcy751	PRJNA1405458	SAMN54742412	JBWQAP000000000
MCy9000	SBAn001	PRJNA1405458	SAMN54742413	JBWQAO000000000
MCy9003	SBAr001	PRJNA1405458	SAMN54742414	JBWQAN000000000
MCy9104	Sbcb007	PRJNA1405458	SAMN54742415	JBWQAM000000000
MCy9111	Sbcb014	PRJNA1405458	SAMN54742416	JBWQAL000000000
MCy9112	Sbcb015	PRJNA1405458	SAMN54742417	JBWQAK000000000
MCy9143	SBMe001	PRJNA1405458	SAMN54742418	JBWQAJ000000000
MCy9144	SBMe002	PRJNA1405458	SAMN54742419	JBWQAI000000000
MNa10564	SBNc005	PRJNA1405458	SAMN54742420	JBWQAH000000000
MNa10565	SBNc006	PRJNA1405458	SAMN54742421	JBWQAG000000000
MNa10566	SBNc007	PRJNA1405458	SAMN54742422	JBWQAF000000000
MNa10571	SBNc012	PRJNA1405458	SAMN54742423	JBWQAE000000000
MNa10572	SBNc013	PRJNA1405458	SAMN54742424	JBWQAD000000000
MNa10576	SBNc016	PRJNA1405458	SAMN54742425	JBWQAC000000000
MNa10588	SBSr024	PRJNA1405458	SAMN54742426	JBWQAB000000000
MNa10913	MNa10913	PRJNA1405458	SAMN54742427	JBWQAA000000000
MNa11354	SBNc018	PRJNA1405458	SAMN54742428	JBWPZZ000000000
MNa11579	SBNc019	PRJNA1405458	SAMN54742429	JBWPZY000000000
MNa11734	MNa11734	PRJNA1405458	SAMN54742430	JBWPZX000000000
MNa12152	SBNa048	PRJNA1405458	SAMN54742431	JBWPZW000000000
MNa12472	SBNc020	PRJNA1405458	SAMN54742432	JBWPZV000000000
MNa13079	SBNc031	PRJNA1405458	SAMN54742433	JBWPZU000000000
MNa13422	SBNa055	PRJNA1405458	SAMN54742434	JBWPZT000000000
MNa9300	SBNa001	PRJNA1405458	SAMN54742435	JBWPZS000000000
MSr10579	SBAe005	PRJNA1405458	SAMN54742436	JBWPZR000000000
MSr10681	SBSr030	PRJNA1405458	SAMN54742437	JBWPZQ000000000
MSr11462	MSr11462	PRJNA1405458	SAMN54742438	JBWPZP000000000
MSr11498	SBCm030	PRJNA1405458	SAMN54742439	JBWPZO000000000

MSr11506	SBSr060	PRJNA1405458	SAMN54742440	JBWPZN000000000
MSr11624	SBSr073	PRJNA1405458	SAMN54742441	JBWPZM000000000
MSr11763	SBSa002	PRJNA1405458	SAMN54742442	JBWPZL000000000
MSr12020	SBSr124	PRJNA1405458	SAMN54742443	JBWPZK000000000
MSr12620	SBMi007	PRJNA1405458	SAMN54742444	JBXDQJ000000000
MSr12847	SBSr235	PRJNA1405458	SAMN54742445	JBWPZJ000000000
MSr12968	SBSa005	PRJNA1405458	SAMN54742446	JBWPZI000000000
MSr12969	SBSa006	PRJNA1405458	SAMN54742447	JBWPZH000000000
MSr13017	SBSr261	PRJNA1405458	SAMN54742448	JBWPZG000000000
MSr13228	SBSr262	PRJNA1405458	SAMN54742449	JBWPZF000000000
MSr13276	SBSr267	PRJNA1405458	SAMN54742450	JBWPZE000000000
MSr13296	SBSr140	PRJNA1405458	SAMN54742451	JBWPZD000000000
MSr13304	SBSr221	PRJNA1405458	SAMN54742452	JBWPZC000000000
MSr13305	SBSr222	PRJNA1405458	SAMN54742453	JBWPZB000000000
MSr13327	SBSr277	PRJNA1405458	SAMN54742454	JBWPZA000000000
MSr13329	SBSr279	PRJNA1405458	SAMN54742455	JBWPYZ000000000
MSr13331	SBSr281	PRJNA1405458	SAMN54742456	JBWPYY000000000
MSr13458	SBSr283	PRJNA1405458	SAMN54742457	JBWPYX000000000
MSr13459	SBSr284	PRJNA1405458	SAMN54742458	JBWPYW000000000
MSr13460	SBSr285	PRJNA1405458	SAMN54742459	JBWPYV000000000
MSr14235	SBSr103	PRJNA1405458	SAMN54742460	JBWPYU000000000
MSr14236	SBSr113	PRJNA1405458	SAMN54742461	JBWPYT000000000
MSr14237	SBAe005or	PRJNA1405458	SAMN54742462	JBWPYS000000000
MSr9030	SBCm007	PRJNA1405458	SAMN54742463	JBWPYR000000000
MSr9032	SBCm009	PRJNA1405458	SAMN54742464	JBXDQI000000000
MSr9139	SBSr007	PRJNA1405458	SAMN54742465	JBWPYQ000000000
MSr9331	SBSr003	PRJNA1405458	SAMN54742483	JBWPYP000000000
MSr9332	SBSr004	PRJNA1405458	SAMN54742466	JBWQDB000000000
MSr9506	SBSr017	PRJNA1405458	SAMN54742467	JBWPYO000000000
MSr9521	SBSr021	PRJNA1405458	SAMN54742468	JBWPYN000000000
MSr9528	SBSr022	PRJNA1405458	SAMN54742469	JBWPYM000000000
MSr9556	SBMi003	PRJNA1405458	SAMN54742470	JBWPYL000000000
Mxf50	Mxf50	PRJNA1405458	SAMN54742471	JBWPYK000000000
Nae487	Nae487	PRJNA1405458	SAMN54742472	JBWPYJ000000000
NOSO3	NOSO-3	PRJNA1405458	SAMN54742473	JBWPYI000000000
NOSO5	NOSO5	PRJNA1405458	SAMN54742474	JBWPYH000000000
NOSO6	NOSO-6	PRJNA1405458	SAMN54742475	JBWPYG000000000
Pde77	Pde77	PRJNA1405458	SAMN54742476	JBWPYF000000000
Plt4	SBJa001	PRJNA1405458	SAMN54742477	JBWPYE000000000
Plvt1	Plvt1	PRJNA1405458	SAMN54742478	JBWPYD000000000
Plvt3	Pl_vt3	PRJNA1405458	SAMN54742479	JBWPYC000000000
SBKo001	SBKo001T	PRJNA1405458	SAMN54742480	JBWPYB000000000
SBNa008	SBNa008	PRJNA1405458	SAMN54742481	JBXDQH000000000
SBSr002	SBSr002	PRJNA1405458	SAMN54742482	JBWPYA000000000
Sga15	Sga15	PRJNA1405458	SAMN54742484	JBWPXZ000000000
Sga32	Sga32	PRJNA1405458	SAMN54742485	JBWPXY000000000
Soce1000	Soce1000	PRJNA1405458	SAMN54742486	JBWPXX000000000
Soce1078	Soce1078	PRJNA1405458	SAMN54742487	JBWPXW000000000
Soce1525	Soce1525	PRJNA1405458	SAMN54742488	JBXDQG000000000
Soce269	Soce269	PRJNA1405458	SAMN54742489	JBXDQF000000000

Soce321	Soce321	PRJNA1405458	SAMN54742490	JBWPXV000000000
Soce341	Soce341	PRJNA1405458	SAMN54742491	JBWPXU000000000
Soce426	Soce426	PRJNA1405458	SAMN54742492	JBWPXT000000000
Soce429	Soce429	PRJNA1405458	SAMN54742493	JBXDQE000000000
Soce448	Soce448	PRJNA1405458	SAMN54742494	JBWPXS000000000
Soce62	Soce62	PRJNA1405458	SAMN54742495	JBWPXR000000000
Soce887	Soce887	PRJNA1405458	SAMN54742496	JBWPXQ000000000
Syt2	SBCm019	PRJNA1405458	SAMN54742497	JBWPXP000000000

Supplementary References

1. Das, J. *et al.* Genomic insights into novel predatory myxobacteria isolated from human feces. *Microbiol. Spectr.* **13**, e02147-24 (2025). https://doi.org/10.1128/spectrum.02147-24
2. LINK, H. Observationes in ordines plantarum naturales. Dissertatio I. *Mag Ges Naturf Freunde Berl.* **3**, 3–42 (1809)
3. McCURDY, H. D. Studies on the Taxonomy of the Myxobacterales: IV. Melittangium. *Int. J. Syst. Evol. Microbiol.* **21**, 50–54 (1971). https://doi.org/10.1099/00207713-21-1-50
4. Blin, K. *et al.* antiSMASH 8.0: extended gene cluster detection capabilities and analyses of chemistry, enzymology, and regulation. *Nucleic Acids Res.* **53**, W32–W38 (2025). https://doi.org/10.1093/nar/gkaf334
5. Stachelhaus, T., Mootz, H. D. & Marahiel, M. A. The specificity-conferring code of adenylation domains in nonribosomal peptide synthetases. *Chem. Biol.* **6**, 493–505 (1999). https://doi.org/10.1016/S1074-5521(99)80082-9
6. Felnagle, E. A. *et al.* Nonribosomal Peptide Synthetases Involved in the Production of Medically Relevant Natural Products. *Mol. Pharm.* **5**, 191–211 (2008). https://doi.org/10.1021/mp700137g
7. Peng, H., Schmiederer, J., Chen, X., Panagiotou, G. & Kries, H. Controlling Substrate- and Stereospecificity of Condensation Domains in Nonribosomal Peptide Synthetases. *ACS Chem. Biol.* **19**, 599–606 (2024). https://doi.org/10.1021/acschembio.3c00678
8. Jansen, R. *et al.* Antibiotika aus Gleitenden Bakterien, XXXVII. Sorangicin A, ein hochwirksames Antibiotikum mit neuartiger Makrolid-Polyether-Struktur aus Sorangium cellulosum, So ce12: Spektroskopische Strukturaufklärung, Kristall- und Lösungsstruktur. *Liebigs Ann. Chem.* **1989**, 111–119 (1989). https://doi.org/10.1002/jlac.198919890124
9. Jansen, R., Höfle, G., Irschik, H., Reichenbach, H. & Wray, V. Antibiotika aus Gleitenden Bakterien, XXXVIII. Natürliche Strukturvarianten von Sorangicin A aus Sorangium cellulosum, So ce12. *Liebigs Ann. Chem.* **1989**, 213–222 (1989). https://doi.org/10.1002/jlac.198919890142
10. Garcia, R. *et al.* Discovery of the Pendulisporaceae: An extremotolerant myxobacterial family with distinct sporulation behavior and prolific specialized metabolism. *Chem* **10**, 2518–2537 (2024). https://doi.org/10.1016/j.chempr.2024.04.019
11. Irschik, H. *et al.* Analysis of the Sorangicin Gene Cluster Reinforces the Utility of a Combined Phylogenetic/Retrobiosynthetic Analysis for Deciphering Natural Product Assembly by trans-AT PKS. *ChemBioChem* **11**, 1840–1849 (2010). https://doi.org/10.1002/cbic.201000313
12. Helfrich, E. J. N. *et al.* Automated structure prediction of trans-acyltransferase polyketide synthase products. *Nat. Chem. Biol.* **15**, 813–821 (2019). https://doi.org/10.1038/s41589-019-0313-7
13. Helfrich, E. J. N. & Piel, J. Biosynthesis of polyketides by trans-AT polyketide synthases. *Nat. Prod. Rep.* **33**, 231–316 (2016). https://doi.org/10.1039/C5NP00125K
14. Seemann, T. barrnap 1.10.5: Rapid microbial RNA annotation (2026). https://github.com/tseemann/barrnap
15. Shen, W., Sipos, B. & Zhao, L. SeqKit2: A Swiss army knife for sequence and alignment processing. *iMeta* **3**, e191 (2024). https://doi.org/10.1002/imt2.191

16. Camacho, C. *et al.* BLAST+: architecture and applications. *BMC Bioinformatics* **10**, 421 (2009). https://doi.org/10.1186/1471-2105-10-421
17. Jain, C., Rodriguez-R, L. M., Phillippy, A. M., Konstantinidis, K. T. & Aluru, S. High throughput ANI analysis of 90K prokaryotic genomes reveals clear species boundaries. *Nat. Commun.* **9**, 5114 (2018). https://doi.org/10.1038/s41467-018-07641-9
18. Navarro-Muñoz, J. C. *et al.* A computational framework to explore large-scale biosynthetic diversity. *Nat. Chem. Biol.* **16**, 60–68 (2020). https://doi.org/10.1038/s41589-019-0400-9
19. Kautsar, S. A., van der Hooft, J. J. J., de Ridder, D. & Medema, M. H. BiG-SLiCE: A highly scalable tool maps the diversity of 1.2 million biosynthetic gene clusters. *GigaScience* **10**, giaa154 (2021). https://doi.org/10.1093/gigascience/giaa154
20. Terlouw, B. R. *et al.* MIBiG 3.0: a community-driven effort to annotate experimentally validated biosynthetic gene clusters. *Nucleic Acids Res.* **51**, D603–D610 (2023). https://doi.org/10.1093/nar/gkac1049
21. RDKit. https://www.rdkit.org/
22. Pedregosa, F. *et al.* Scikit-learn: Machine Learning in Python. *Mach. Learn. PYTHON*
23. Blin, K., Shaw, S., Medema, M. H. & Weber, T. The antiSMASH database version 4: additional genomes and BGCs, new sequence-based searches and more. *Nucleic Acids Res.* **52**, D586–D589 (2024). https://doi.org/10.1093/nar/gkad984
24. Geneious | Bioinformatics Software for Sequence Data Analysis. *Geneious* https://www.geneious.com/
25. Hiltemann, S. D. *et al.* Galaxy mothur Toolset (GmT): a user-friendly application for 16S rRNA gene sequencing analysis using mothur. *GigaScience* **8**, giy166 (2019). https://doi.org/10.1093/gigascience/giy166
26. Yilmaz, P. *et al.* The SILVA and "All-species Living Tree Project (LTP)" taxonomic frameworks. *Nucleic Acids Res.* **42**, D643–D648 (2014). https://doi.org/10.1093/nar/gkt1209
27. Pruesse, E., Peplies, J. & Glöckner, F. O. SINA: Accurate high-throughput multiple sequence alignment of ribosomal RNA genes. *Bioinformatics* **28**, 1823–1829 (2012). https://doi.org/10.1093/bioinformatics/bts252
28. Terlouw, B. R. *et al.* PARAS: high-accuracy machine-learning of substrate specificities in nonribosomal peptide synthetases. Preprint at https://doi.org/10.1101/2025.01.08.631717 (2025)
29. Kawata, J., Naoe, T., Ogasawara, Y. & Dairi, T. Biosynthesis of the Carbonylmethylene Structure Found in the Ketomemicin Class of Pseudotripeptides. *Angew. Chem.* **129**, 2058–2061 (2017). https://doi.org/10.1002/ange.201611005
30. Panter, F., Krug, D., Baumann, S. & Müller, R. Self-resistance guided genome mining uncovers new topoisomerase inhibitors from myxobacteria. *Chem. Sci.* **9**, 4898–4908 (2018). https://doi.org/10.1039/C8SC01325J
